# Police stops to reduce crime: A systematic review and meta‐analysis

**DOI:** 10.1002/cl2.1302

**Published:** 2023-01-10

**Authors:** Kevin Petersen, David Weisburd, Sydney Fay, Elizabeth Eggins, Lorraine Mazerolle

**Affiliations:** ^1^ Department of Criminology, Law and Society George Mason University Fairfax Virginia USA; ^2^ Institute of Criminology, Faculty of Law Hebrew University Jerusalem Israel; ^3^ School of Applied Psychology Griffith University Brisbane Australia; ^4^ School of Social Sciences University of Queensland, St Lucia Campus St Lucia Australia

## Abstract

**Background:**

Police‐initiated pedestrian stops have been one of the most widely used crime prevention tactics in modern policing. Proponents have long considered police stops to be an indispensable component of crime prevention efforts, with many holding them responsible for the significant reductions in violent crime observed across major US cities in recent decades. Critics, however, have taken issue with the overuse of pedestrian stops, linking them to worsening mental and physical health, attitudes toward the police, and elevated delinquent behavior for individuals directly subject to them. To date, there has been no systematic review or meta‐analysis on the effects of these interventions on crime and individual‐level outcomes.

**Objectives:**

To synthesize the existing evaluation research regarding the impact of police‐initiated pedestrian stops on crime and disorder, mental and physical health, individual attitudes toward the police, self‐reported crime/delinquency, violence in police‐citizen encounters, and police misbehavior.

**Search Methods:**

We used the Global Policing Database, a repository of all experimental and quasi‐experimental evaluations of policing interventions conducted since 1950, to search for published and unpublished evaluations of pedestrian stop interventions through December of 2019. This overarching search was supplemented by additional searches of academic databases, gray literature sources, and correspondence with subject‐matter experts to capture eligible studies through December 2021.

**Selection Criteria:**

Eligibility was limited to studies that included a treatment group of people or places experiencing pedestrian stops and a control group of people or places not experiencing pedestrian stops (or experiencing a lower dosage of pedestrian stops). Studies were required to use an experimental or quasi‐experimental design and evaluate the intervention using an outcome of area‐level crime and disorder, mental or physical health, individual or community‐level attitudes toward the police, or self‐reported crime/delinquency.

**Data Collection and Analysis:**

We adopted standard methodological procedures expected by the Campbell Collaboration. Eligible studies were grouped by conceptually similar outcomes and then analyzed separately using random effects models with restricted maximum likelihood estimation. Treatment effects were represented using relative incident rate ratios, odds ratios, and Hedges' *g* effect sizes, depending on the unit of analysis and outcome measure. We also conducted sensitivity analyses for several outcome measures using robust variance estimation, with standard errors clustered by each unique study/sample. Risk of bias was assessed using items adapted from the Cochrane randomized and non‐randomized risk of bias tools.

**Results:**

Our systematic search strategies identified 40 eligible studies corresponding to 58 effect sizes across six outcome groupings, representing 90,904 people and 20,876 places. Police‐initiated pedestrian stop interventions were associated with a statistically significant 13% (95% confidence interval [CI]: −16%, −9%, *p* < 0.001) reduction in crime for treatment areas relative to control areas. These interventions also led to a diffusion of crime control benefits, with a statistically significant 7% (95% CI: −9%, −4%, *p* < 0.001) reduction in crime for treatment displacement areas relative to control areas. However, pedestrian stops were also associated with a broad range of negative individual‐level effects. Individuals experiencing police stops were associated with a statistically significant 46% (95% CI: 24%, 72%, *p* < 0.001) increase in the odds of a mental health issue and a 36% (95% CI: 14%, 62%, *p* < 0.001) increase in the odds of a physical health issue, relative to control. Individuals experiencing police stops also reported significantly more negative attitudes toward the police (*g* = −0.38, 95% CI: −0.59, −0.17, *p* < 0.001) and significantly higher levels of self‐reported crime/delinquency (*g* = 0.30, 95% CI: 0.12, 0.48, *p* < 0.001), equating to changes of 18.6% and 15%, respectively. No eligible studies were identified measuring violence in police‐citizen encounters or officer misbehavior. While eligible studies were often considered to be at moderate to high risk of bias toward control groups, no significant differences based on methodological rigor were observed. Moderator analyses also indicated that the negative individual‐level effects of pedestrian stops may be more pronounced for youth, and that significant differences in effect sizes may exist between US and European studies. However, these moderator analyses were limited by a small number of studies in each comparison, and we were unable to compare the effects of police stops across racial groupings.

**Authors' Conclusions:**

While our findings point to favorable effects of pedestrian stop interventions on place‐based crime and displacement outcomes, evidence of negative individual‐level effects makes it difficult to recommend the use of these tactics over alternative policing interventions. Recent systematic reviews of hot spots policing and problem‐oriented policing approaches indicate a more robust evidence‐base and generally larger crime reduction effects than those presented here, often without the associated backfire effects on individual health, attitudes, and behavior. Future research should examine whether police agencies can mitigate the negative effects of pedestrian stops through a focus on officer behavior during these encounters.

## 1 PLAIN LANGUAGE SUMMARY

### 1.1 Police stops are associated with reductions in crime but also a broad range of negative individual‐level outcomes

Police stop interventions produce meaningful and significant reductions in crime without evidence of spatial displacement. However, people subject to stops are associated with significantly less desirable mental and physical health outcomes, attitudes toward police, and self‐reported crime/delinquency. For some outcome measures, the negative effects of pedestrian stops are considerably more pronounced for youth, though the data did not permit a comparison of individual effects by race.

### 1.2 What is this review about?

Police stops have become one of the most controversial yet widely‐used crime prevention strategies in modern policing. This intervention involves the police‐initiated stop of an individual (or group of individuals) on the street, for the purpose of investigation and/or questioning. Police stops have been commonly used as a tactic to combat violent and gun‐related crime.

The current review assesses the effect of police stops (used interchangeably here with “pedestrian stops”) on both place‐based and person‐based outcomes, including crime, spatial displacement, mental health, physical health, attitudes toward the police, and self‐reported crime/delinquency.

**What is the aim of this review?**

This Campbell systematic review examines the effects of police‐initiated pedestrian stops on both place‐based and person‐based outcomes. It synthesizes results from 40 studies across six outcome groupings. Studies were predominately conducted in the USA.

### 1.3 What studies are included?

Forty studies published between 1970‐2021 are included in this review. Eligibility was limited to experimental and quasi‐experimental studies with a treatment group of people or places that experienced police stops and a control group of people or places that did not experience police stops (or experienced a lower dosage of stops).

Studies focusing only on police‐initiated traffic stops were excluded from this review. Only one eligible study was a randomized controlled trial, 33 studies were conducted in the USA, and seven were conducted in Europe.

### 1.4 What are the main findings of this review?

Police stop interventions lead to significant reductions in area‐level crime with evidence of a diffusion of crime control benefits to nearby areas. However, methodological difficulties limit the strength of the causal inferences derived from these studies; further research is needed.

Individuals stopped by police are associated with significantly higher odds of both mental and physical health issues, significantly more negative attitudes toward the police, and elevated levels of self‐reported crime/delinquency. The impact of a direct stop experience on mental health issues is also considerably larger for youth, compared to adults.

Despite this finding, place‐based studies incorporating community surveys suggest that stop interventions do not impact community‐level attitudes toward the police, and thus the negative effects of these interventions may be limited to the individuals directly experiencing them.

The findings of this review should be interpreted with caution, however, as only one randomized experiment assessing crime prevention outcomes was identified, and person‐based studies were often unable to establish temporal ordering between the treatment and outcome measures.

### 1.5 What do the findings of this review mean?

Policing efforts focused on high‐volume pedestrian stops are likely to reduce crime but may do so at the cost of negative health outcomes, negative attitudes toward the police, and higher levels of delinquency for individuals subject to the intervention. Given the net‐widening effects of pedestrian stops (i.e., low proportions of stops lead to arrests or weapon seizures), these interventions may produce more harm than good. Police agencies should carefully weigh the potential benefits and harms associated with these interventions.

Furthermore, recent reviews on tactics such as hot spots policing and problem‐oriented policing have demonstrated larger reductions in crime without similar backfire effects. The evidence‐base for these tactics is also of considerably higher methodological rigor, generating stronger conclusions regarding program effectiveness. While it is possible that police agencies can mitigate the negative effects of pedestrian stops through a focus on improving officer conduct during police‐citizen encounters, this review is unable to provide evidence of this effect.

### 1.6 How up‐to‐date is this review?

The authors of this review employed search strategies intended to capture studies through December 2021.

## 2 BACKGROUND

### 2.1 The problem, condition, or issue

The use of pedestrian stops has been one of the most common yet controversial proactive strategies in modern policing (Weisburd & Majmundar, 2018). The pedestrian stop (also known as stop and frisk, *Terry* stops, street pops, stop and search, street stops, etc.) is often defined as the process by which “officers stop, and potentially question and search, people in the communities they are patrolling” (Lachman et al., 2012, p. 1). These tactics have been a staple in policing for generations, but they gained legitimacy with the landmark US Supreme Court decision in *Terry v. Ohio* (1968)—which allows police officers discretion to conduct an investigatory stop of an individual given reasonable suspicion that the individual has committed a crime or is in the process of committing a crime, and discretion to frisk (or pat‐down) the individual given reasonable suspicion that they are carrying a weapon (see Jones‐Brown et al., 2010).

Often termed “stop, question, and frisk (SQF)” (Rosenfeld & Fornango, 2014, p. 96), evidence suggests that many US police departments began using pedestrian stops widely as a proactive policing strategy in the 1990s and early 2000s (Gelman et al., 2007; White & Fradella, 2016). In New York City alone, recorded SQFs increased from 160,851 in 2003 to 685,000 in 2011 (Weisburd et al., 2016), and similar increases have been noted in other US cities such as Philadelphia and Los Angeles (Jones‐Brown et al., 2010; Saul, 2016). Police “stop and search” (McCandless et al., 2016, p. 2) powers have also been noted in the UK, where targeted pedestrian stops have been used as a strategy to reduce knife crime (Tiratelli et al., 2018), and in other European countries such as Bulgaria, Hungary, and Spain, often for the purpose of conducting identity checks related to criminal investigations (Miller et al., 2008). In this context, pedestrian stops have been used as primary components in various proactive policing interventions, including crackdowns (Sherman, 1990), efforts to reduce illegal gun carrying (Koper & Mayo‐Wilson, 2006), directed patrol interventions (Ratcliffe et al., 2011), and hot spots policing interventions (Braga et al., 2019).

While advocates have considered pedestrian stops to be a contributing factor to decreasing levels of crime in American cities (Baker & Goldstein, 2012), critics have pointed to the low success rates (i.e., low proportions of stops that lead to arrest or weapon seizure) and racial disparity associated with these strategies as evidence that such tactics represent an illegal and unjust use of police power (Fagan & Davies, 2000; Gelman et al., 2007; Rosenfeld & Fornango, 2014). Racial and ethnic profiling has also been a concern on an international level, with researchers noting racially disparate stop rates in several European countries, without clear evidence that these strategies have produced meaningful crime reductions (McCandless et al., 2016; Miller et al., 2008; Tiratelli et al., 2018). Additionally, academic and social discourse has highlighted the potential deleterious effects of pedestrian stops on outcomes such as mental and physical health (see Geller et al., 2014; McFarland et al., 2019), attitudes toward the police (see Harris & Jones, 2020; Rosenbaum et al., 2005; Tyler et al., 2014), and even future delinquency and offending (Wiley & Esbensen, 2016; Wiley et al., 2013). In other words, though the goal of pedestrian stops may be to produce a general deterrent effect, the intervention may also produce latent backfire effects for the individuals directly subjected to them.

Despite such challenges, practitioners still view pedestrian stops as an important element of proactive crime prevention efforts (D'Onfrio, 2019; Terkel, 2013), making an understanding of their effects on crime, individuals, and the larger community increasingly important. Studying the crime reduction effects of pedestrian stop tactics has been difficult, however, given that stops have been used as components of numerous different interventions and have been evaluated using a variety of different techniques (see Koper & Mayo‐Wilson, 2006; MacDonald et al., 2016; Rosenfeld & Fornango, 2014; Sherman, 1990; Smith & Purtell, 2008; Weisburd et al., 2016). Thus, the current work attempts to fill this gap by conducting a systematic review and meta‐analysis on the impact of pedestrian stops as a proactive policing strategy for reducing crime. Additionally, we seek to examine the effects of pedestrian stops on both the individuals and communities subjected to these strategies.

### 2.2 The intervention

Pedestrian stops involve the police‐initiated stop of an individual (or group of individuals) on the street for the purpose of investigation and/or questioning (Lachman et al., 2012). In most cases, the officer must have reasonable suspicion that a person is involved in criminal activity for a stop to occur, and based on the level of suspicion, a frisk or search of the person may be conducted. However, in certain contexts stops may be conducted without suspicion or the threshold for reasonable suspicion may vary. In the UK, the Criminal Justice and Public Order Act of 1994 permits suspicion‐less stops in high‐risk areas with approval from an authorizing officer (Lennon, 2013, 2015). Police officers in the UK and other European countries are also permitted to conduct suspicion‐less stops of people in authorized areas as a proactive counter‐terrorism measure (Lennon, 2013). Similarly, the US Supreme Court has ruled that the amount of crime in a given area can be used as a factor in an officer's determination of reasonable suspicion (Gelman et al., 2007; *Illinois v. Wardlow*). Thus, it is important to note that while pedestrian stops are often reactive in nature, in that they require prior indication of suspicious behavior or criminal activity, they may also be used proactively. In this regard, it is important to distinguish between pedestrian stops at the individual level and pedestrian stops as employed in proactive policing interventions. Proactive policing involves “policing strategies that have as one of their goals the prevention or reduction of crime and disorder and that are not reactive in terms of focusing primarily on uncovering ongoing crime or on investigating or responding to crimes once they have occurred” (Weisburd & Majmundar, 2018, p. 1). Thus, while pedestrian stops conducted in response to observed or reported criminal behavior are reactive in nature, using pedestrian stops as part of a coordinated effort to deter or prevent crime is consistent with the tenets of proactive policing.

Pedestrian stops may be employed as distinct proactive policing strategies or used as components of larger interventions such as short‐term police crackdowns (Sherman, 1990), directed patrol presence (McGarrell et al., 2002; Ratcliffe et al., 2011), or hot spots policing (Weisburd et al., 2014). While pedestrian stops have primarily been implemented as a tactic to reduce violent and/or weapon‐related crime (Koper & Mayo‐Wilson, 2006; Ratcliffe et al., 2011; Sherman et al., 1995), they have also been used to target other crime/disorder problems (e.g., drug‐related crime, see Geller & Fagan, 2010; Levine & Small, 2008). Additionally, natural variation in the use of pedestrian stops across geographic areas and/or police jurisdictions means that certain individuals are exceedingly likely to be subject to stops, while others are not (see Fagan & Davies, 2000). This draws attention to the importance of both the individual and community‐level elements of the intervention. Pedestrian stops represent a policing tactic acutely targeted at specific people, despite the intent to produce larger community and area‐level reductions in crime and disorder. The current review includes any policing intervention employing pedestrian stops as a primary component, regardless of what (if any) specific crime/disorder outcome is being targeted. Here, the term “policing intervention” refers to both specific programmatic approaches targeted at particular areas (e.g., hot spots or hot neighborhoods), as well as natural variation or the generalized use of pedestrian stops as a crime prevention approach (similar to the use of preventive patrols to reduce crime in a city). Thus, the current review examines both place‐based and individual‐level impacts of the intervention.

### 2.3 How the intervention might work

It has often been argued that offenders weigh the potential costs and benefits associated with a criminal act. Accordingly, individuals may be deterred from committing crime in situations where the potential costs of crime outweigh the potential benefits (Beccaria, 1986; Bentham, 1988; Durlauf & Nagin, 2011; Nagin, 2013). Pedestrian stops may deter crime by increasing these perceived costs, and likewise the perceived certainty of apprehension if a crime is committed (Lachman et al., 2012). In other words, people who have been personally stopped by the police may alter their behavior or avoid the area where the stop occurred to mitigate their risk of punishment, while people who become vicariously aware of the pedestrian stop intervention may pre‐emptively do the same (Rosenfeld & Fornango, 2014). If pedestrian stops result in the seizure of weapons or other items that are used to commit crime, they may also produce an incapacitation effect by preventing access to the tools needed to commit criminal acts (see Sherman et al., 1995). Alternatively, it is possible that pedestrian stop strategies deter crime merely through increasing police presence in high‐crime areas. In this context, the deterrent effect is not necessarily related to the strategy itself, but rather to the increased police visibility in the area.

It is key in any policing program to disentangle the impacts of specific policing strategies on both the individuals targeted and the communities in which they are applied. Advocates of pedestrian stops focus on the benefits of reduced crime in the community (D'Onfrio, 2019; Terkel, 2013). However, other research suggests that pedestrian stops are often perceived as unfair/unlawful, producing backfire effects on community attitudes toward the police (Miller et al., 2000; Tyler et al., 2014). That is, police‐initiated stops may reduce feelings of police legitimacy among the individuals stopped or the communities in which stops are implemented. Rooted in this is a deep‐seated distrust of policing and a history of perceived oppression within high‐crime minority communities (see Braga et al., 2019). Depending on the nature of the interaction, individuals may feel that they are being stopped without proper cause and/or that their personal freedom is being unjustly restricted, leading to a reduction in attitudes favorable to the police (see Baćak & Apel, 2021; Harris & Jones, 2020; Tyler et al., 2014). For instance, research has suggested that in New York City, Black individuals are over six times more likely to be stopped by police than White individuals, and that the rate of success during these stops (operationalized as the rate of drug/weapon seizures or arrests) is often less than 3% for seizures and 7% for arrests (see Geller & Fagan, 2010; Gelman et al., 2007; Jones‐Brown et al., 2010). Thus, the vast majority of police stops appear to be conducted against disadvantaged populations that are neither committing an arrestable offense, carrying weapons, or carrying contraband.

There is also evidence to suggest that pedestrian stops can have deleterious effects on individuals' mental and physical health. Stops are often perceived as traumatic, invasive, and stressful, linking them to worsening anxiety, trauma, depression, sleep behavior, and physical functioning (Baćak & Apel, 2020; Geller et al., 2014; Hirschtick et al., 2020; Testa et al., 2021). In addition, pedestrian stops may be conducted in a rough manner, leading to the use‐of‐force that results in physical injury to the individual stopped (Brunson & Weitzer, 2009; Levine & Small, 2008). If these experiences happen in large numbers, vicarious knowledge of such incidents may further impact community perceptions of the police (Miller & D'Souza, 2015). These deleterious effects may also extend to behavioral patterns. Labeling theorists suggest that the imposition of a criminal sanction leads to the internalization of a deviant identity, socialization with deviant peers, and even defiance toward conventional society (Lemert, 1951; Sherman, 1993; Paternoster & Iovanni, 1989). Under this framework, contact with the criminal justice system only serves to worsen future behavior (Schur, 1973), and thus aggressive police stops may elevate individual‐level delinquent/criminal offending (see Lee et al., 2017; Wiley & Esbensen, 2016; Wiley et al., 2013).

Concern regarding the negative latent effects of pedestrian stops is particularly salient among certain sub‐populations of people. Adolescent youth are in a critical developmental period and may be particularly susceptible to stressful/traumatic events and deviant labeling (Geller, 2017; Jackson et al., 2021; Wiley & Esbensen, 2016). In addition, racial minorities are disproportionately exposed to proactive policing tactics such as pedestrian stops (Braga et al., 2019). Given a history of mistreatment and abuse at the hands of the police, these experiences may lead to elevated levels of stress and further compound pre‐existing beliefs about racial stereotyping (see Baćak & Nowotny, 2020; Geller, 2017; Wheelock et al., 2019). Thus, while pedestrian stops have a clear theoretical linkage to area‐level crime reduction benefits, they also have equally clear linkages to deleterious community and individual‐level outcomes.

### 2.4 Why it is important to do the review

Proactive policing tactics play an important role in crime prevention (Skogan & Frydl, 2004; Telep & Weisburd, 2012; Weisburd & Eck, 2004; Weisburd & Majmundar, 2018). However, the effects of proactive interventions vary greatly by the type of intervention and the manner in which the intervention is applied. Some tactics raise critical questions about the impacts of policing on the communities that they serve and the individuals subject to the intervention (Braga et al., 2019; Tyler et al., 2014).

Police have long felt that pedestrian stops can have an important general and specific deterrent value in preventing crime. Research evidence supporting this view began to develop in the 1990s with evaluations of police crackdowns (Sherman, 1990). There is evidence that many cities across the US were using pedestrian stops as a key crime prevention tool (Gelman et al., 2007; White & Fradella, 2016), and indeed the use of pedestrian stops has often correlated with decreasing crime in major US cities (Weisburd et al., 2014). But a rigorous assessment of the crime prevention outcomes associated with pedestrian stops has not been developed to date. A key contribution of our review is the attempt to identify whether pedestrian stops reduce crime, and if so to identify the size of that impact. Given controversies about the use of pedestrian stops as a crime prevention strategy, it is important to understand how much benefit (if any) it provides for public safety.

In recent years, pedestrian stop tactics have come under increasing legal scrutiny. For example, a federal district court ruling in *Floyd v. City of New York* (2013) found the New York City Police Department's (NYPD) use of SQF unconstitutional on the basis of racial disparity. Similar lawsuits have been brought against other US police departments during the past decade (American Civil Liberties Union, 2010), and the perceived abuse of stop and search powers has led to riots and legal challenges in several European countries as well (Bradford, 2017; Lennon & Murray, 2018; Murray et al., 2021).

Due to these concerns, pedestrian stop tactics have become extremely controversial, and recent years have seen the use of such stops decrease substantially in major cities such as New York and Philadelphia (McNeil, 2020; Weisburd et al., 2016), as well as in European countries such as England and Scotland (Lennon & Murray, 2018; Tiratelli et al., 2018). There has even been a growing call among many to do away with pedestrian stop tactics entirely (see Baker & Goldstein, 2012). Yet, existing reviews have often failed to find evidence of negative impacts on community evaluations of the police—though negative effects on people who are stopped has a stronger evidence base (e.g., see Weisburd & Majmundar, 2018). Thus, it is increasingly important to determine if pedestrian stops, developed to reduce crime, produce negative consequences for the individuals and communities affected by them. To date, no review has systematically assessed these outcomes or simultaneously considered them alongside each other. Such a review is critical for informed crime prevention policy that weighs all potential costs and benefits.

## 3 OBJECTIVES

Given that pedestrian stop tactics have garnered controversy and concern over their potential effects on crime (see MacDonald et al., 2016; Rosenfeld & Fornango, 2014; Weisburd et al., 2016), the community (see Baker & Goldstein, 2012; Gelman et al., 2007; Miller et al., 2000; Tyler et al., 2014) and the individuals subject to them (see Geller et al., 2014; Geller, 2017; McFarland et al., 2019; Wiley et al., 2013), the main objective of this review is to synthesize the impact of pedestrian stops across each of these areas. Specifically, this review seeks to assess the following questions:

- What are the effects of pedestrian stop interventions on area‐level crime and disorder?
- What are the effects of pedestrian stop interventions on individual and community‐level attitudes toward the police?
- What are the effects of pedestrian stops on individual mental and physical health outcomes?
- What are the effects of pedestrian stops on self‐reported crime and/or delinquency?
- What are the effects of pedestrian stops on violence in police‐citizen encounters and officer misbehavior?

Our secondary objective, proposed at the time of protocol publication (Weisburd et al., 2021), was to examine whether the effects of police‐initiated pedestrian stops vary according to the following moderating factors: research design, country, size of geographic area, crime type of focus, and racial composition. Based on the eligible studies identified, we were able to assesses the degree to which heterogeneity in effect sizes might be explained by research design (e.g., matched vs. unmatched designs) and characteristics of the sample (e.g., youth vs. non‐youth samples, size of the geographic area targeted).^1^

## 4 METHODS

### 4.1 Criteria for considering studies for this review

#### 4.1.1 Types of studies

For studies to be considered eligible for this review the evaluation was required to include a treatment group that received a pedestrian stops intervention and a separate comparison group that did not receive a pedestrian stops intervention. Here, the treatment group could be comprised of either geographic areas or individuals, and eligible treatments could include proactive policing interventions, natural variation in the use of pedestrian stops across areas, or natural variation in the prevalence of police stops across individuals. In other words, we included comparisons of areas and individuals that differed naturally in their exposure to police stops, regardless of whether these differences were the result of any planned policing intervention. Eligible comparison conditions could include any group of areas or people that were not exposed to a pedestrian stops intervention or were exposed to a lower dosage of the intervention. For geographic studies, comparison conditions generally involved standard police practices, and for individual‐level studies comparison conditions were generally comprised of individuals who had not directly experienced police stops. Studies were included regardless of their publication status.

Both randomized and quasi‐experimental research designs were considered eligible for inclusion (Campbell & Stanley, 1966; Cook & Campbell, 1979; Shadish et al., 2002). This inclusion threshold was adapted from the inclusion criterion in the Global Policing Database (GPD) protocol (Higginson et al., 2015, pp. 47–48), which was the primary search source for this review. From the GPD, we included the following types of designs:

- Randomized controlled trials (RCTs)
- Matched control group designs with or without pre‐intervention baseline measures (propensity or statistically matched)
- Unmatched control group designs with pre‐intervention measures (difference‐in‐difference analysis)
- Unmatched control group designs with pre‐post intervention measures which allow for difference‐in‐difference analyses
- Unmatched control group designs without pre‐intervention measures where the control group has face validity
- Raw unadjusted correlational designs where the variation in the level of the intervention is compared to the variation in the level of the outcome

Thus, this review includes weaker quasi‐experimental studies with “unmatched” control groups; for example, studies that compared a target area or group to the remainder of a jurisdiction or population. Accordingly, any evaluation of pedestrian stops that included a comparison group or area that did not receive the intervention was considered eligible so long as it met our other inclusion criteria. However, we distinguish between matched and unmatched designs in a subsequent moderator analysis (Section 5.4).

#### 4.1.2 Types of participants

Given our interest in examining the impacts of pedestrian stops on crime, the community, and the individuals subject to these stops, this review includes the following populations:

- Law enforcement officers (including any particular race, ethnicity, gender)
- Citizens (including citizens who are the subjects of pedestrian stops or live in areas subject to stop interventions; and including any race, ethnicity, gender)
- Places (including micro places such as street segments, clusters of addresses, police beats; meso‐places such as neighborhoods and communities; or macro‐places such as entire jurisdictions).

#### 4.1.3 Types of interventions

Studies that evaluated interventions in which police‐initiated pedestrian stops of individuals or groups of individuals (for the purpose of questioning, investigation, and/or frisking and searching) were carried out as a major component of a policing intervention were considered eligible for this review. As previously noted, the term “intervention” included natural variation in general policing approaches throughout a jurisdiction and/or natural variation in exposure to police stops among samples of individuals. That is, any comparison of people or places with differential exposure to pedestrian stops was considered an intervention for the purposes of this review. It is important to note here that our focus was on pedestrian stops, and as such, we excluded studies that were solely or primarily focused on traffic stops. More specifically, our interest was in isolating interventions consistent with the concept of SQF, which is traditionally associated with pedestrian stops (see Jones‐Brown et al., 2010; Lachman et al., 2012). However, we did include studies in which both pedestrian and traffic stops were used, given the often‐overlapping nature of these forms of policing, and so long as pedestrian stops remained a major component of the intervention.

We did not attempt to distinguish between the individual motivations behind pedestrian stops or determine whether stops were used reactively or proactively (i.e., whether stops were in response to observed criminal behavior), but rather focused on the intent of the program in which pedestrian stops were a component. This review was not limited to interventions targeting specific types of crime or disorder (e.g., weapon and drug‐related crime), or any specific type of overarching policing tactic (e.g., hot spots policing, crackdowns, directed patrol, etc.). However, we did exclude studies employing pedestrian stops in a minor capacity relative to other policing tactics (as the effects of the stop component would be difficult to isolate from the other components of the policing intervention).

#### 4.1.4 Types of outcome measures

This review included the following outcome measures. All outcomes were considered primary, and eligible studies were required to report at least one of these measures for inclusion:

- Crime and disorder (including displacement)
- Incidents of violence in police‐citizen encounters
- Officer misbehavior
- Fear of crime
- Attitudes toward or perceptions of the police (e.g., legitimacy, satisfaction, trust, effectiveness)
- Mental health issues
- Physical health issues

Crime/disorder and displacement outcomes were considered eligible if measured using official data (e.g., incident and arrest data, calls for service, crime rates), unofficial crime data (e.g., crime reported by civilians, self‐report delinquency via questionnaires or surveys), and systematic social observations of crime. All types of crime and/or disorder were included in this review (e.g., property, drug, violent crime).

We anticipated that incidents of violence in police‐citizen encounters would be measured through police use‐of‐force reports (Weisburd et al., 2021). We planned to be as discrete as possible, including capturing use‐of‐force that results from suspect resistance and varying levels of force when possible. We also note that this outcome is not necessarily a measure of unjustified use‐of force, and thus distinguished this outcome from officer misbehavior. We anticipated that officer misbehavior would be measured through formal citizen complaints or community surveys reporting on police abuse or violence.

We included studies where fear of crime and attitudes towards police were measured using questionnaires or surveys at the community‐level or taken from individuals who directly experienced police stops, as well as those who did not.

For mental and physical health issues, we included studies that measured these outcomes via self‐reports taken from individuals with direct police stop experience or via official data (e.g., injury data from hospitals), and we included data measured at both the individual‐ and community‐levels of analysis. For the purposes of this review, mental health issues were defined as symptoms or diagnoses related to an established mental health condition or a “clinically significant behavioral or psychological syndrome or pattern that occurs in an individual” (Stein et al., 2010, p. 1760), such as anxiety, post‐traumatic stress disorder (PTSD), suicidality, depression, etc. Physical health issues concerned any characteristic or condition that could directly impact or have implications for physical functioning, such as self‐reported physical health, sleep problems, and/or functional limitations (see e.g., Baćak & Apel, 2020; Testa et al., 2021).

#### 4.1.5 Duration of follow‐up

Eligible studies were not restricted to any particular follow‐up period. At the geographic level, stop interventions are likely to produce short‐term deterrent effects (Sherman, 1990; Weisburd et al., 2016), though the impacts on individuals directly experiencing stops may be long term (see Dennison & Finkeldey, 2021; Wiley et al., 2013). In the protocol for this review (Weisburd et al., 2021), we planned to synthesize studies by length of follow‐up period (<6 months, 6–12 months, >1 year). However, this approach needed to be adapted due to the nature of included studies and is described in the results section.

#### 4.1.6 Types of settings

No restrictions were placed on geographic region, racial, ethnic, or demographic makeup, or written language. We used Google Translate to conduct title and abstract screening for any non‐English language studies, as well as for the main text of any non‐English language articles that required full‐text review.

### 4.2 Search methods for identification of studies

#### 4.2.1 Electronic searches

Our systematic search strategies were led by the GPD research team at the University of Queensland (Elizabeth Eggins and Lorraine Mazerolle) and Queensland University of Technology (Angela Higginson). The GPD is a web‐based and searchable database designed to capture all published and unpublished experimental and quasi‐experimental evaluations of policing interventions conducted since 1950 (http://www.gpd.uq.edu.au). There are no restrictions on the type of policing technique, the type of outcome measure, or the language of the research (Higginson et al., 2015). The GPD is compiled using systematic search and screening techniques, which are reported in Higginson et al. (2015) and summarized in Supporting Information: Appendices A and B. Broadly, the GPD search protocol includes an extensive range of search locations to ensure that both published and unpublished research is captured across criminology and allied disciplines.

To capture eligible studies, we used the following terms related to pedestrian stops to search the GPD corpus of full‐text documents that have been screened as reporting on a quantitative impact evaluation of a policing intervention. Search terms were limited to the title and abstract fields and included studies published between January 1970 and December 2019. This timeframe was chosen based on evidence that police departments began using pedestrian stops as elements of proactive policing interventions toward the end of the 20th Century (White & Fradella, 2016):

- stop*
- SQF
- frisk*
- search*
- “street pop*”
- “street check*”
- “street‐check*”

To extend the timeframe of the GPD search, we conducted an additional search for studies published between January 2020 and December 2021. This search included the same parameters and keywords as those used in the GPD search and utilized the following databases (see Supporting Information: Appendix C):

- Criminal Justice Abstracts (EBSCO)
- National Criminal Justice Reference Service Abstracts (EBSCO)
- SocINDEX (EBSCO)
- Criminal Justice Database (ProQuest)
- Sociology Database (ProQuest)
- Sociological Abstracts (ProQuest)
- PAIS Index (ProQuest)
- Policy File Index (ProQuest)
- ProQuest Dissertations and Theses

#### 4.2.2 Searching other resources

We used several additional strategies to supplement the approaches described above. First, we searched additional databases from Japan, Korea, the Middle East, and Europe by consulting subject guides through the Duke University Library. Specifically, we searched the following databases using keywords related to policing and pedestrian stops consistent with those described in our main search strategies:

- CiNii Articles
- DBpia
- Index Islamicus
- Middle Eastern and Central Asian Studies
- Historical Abstracts

Second, and similar to recent reviews using the GPD (Hinkle et al., 2020; Lum et al., 2020; Mazerolle et al., 2020), we performed hand searches of published volumes of leading journals in criminology from 2019 to 2021 to identify any studies that had yet to be indexed in electronic databases. Third, we conducted forward citation searches using Google Scholar and reference harvesting of prior reviews on related topics (Braga et al., 2019; Koper & Mayo‐Wilson, 2012). Finally, after completing all searches, we e‐mailed our list of eligible studies to the lead authors of these articles to identify any research that the above searches may have missed.^2^

### 4.3 Data collection and analysis

#### 4.3.1 Selection of studies

All search results were first screened on title and abstract content to determine potential relevance to pedestrian stops. As an initial step, two screeners (Petersen and Fay) reviewed the same subset of 25 titles/abstracts to establish inter‐rater reliability. Afterwards, the remaining results were double screened by both authors. All abstracts were reviewed using *Abstrackr*, which is a free online tool designed for abstract screening in systematic reviews (Wallace et al., 2012). We then retrieved a full‐text copy of all results marked as potentially relevant during title/abstract review. These results were also double screened by both reviewers. Any discrepancies in eligibility determinations or studies identified as “on the fence” were discussed among the entire research team before reaching consensus.

#### 4.3.2 Data extraction and management

Eligible studies were double coded by authors KP and SF using the coding sheet in Appendix D. Our coding protocol captured various items related to:

a. Reference information (title, authors, publication etc.)
b. Nature and description of site selection, group, targeted outcome etc.
c. Nature and description of selection of comparison group or period
d. The unit of analysis
e. The sample size
f. Methodological type (RCT, quasi‐experiment, matched vs. unmatched designs)
g. A description of the pedestrian stop intervention
h. Dosage intensity and type
i. Implementation difficulties
j. The statistical test(s) used
k. Reports of statistical significance (if any)
l. Effect size/power (if any)
m. The conclusions drawn by the authors

The research team met frequently to discuss coding items and any discrepancies in coding were discussed among all review authors before coming to a final coding decision. *EpiData Software* (https://www.epidata.dk/index.htm) was used to digitize coding forms and facilitate data entry.

#### 4.3.3 Assessment of risk of bias in included studies

Six items adapted from the Cochrane randomized and non‐randomized risk of bias tools (Sterne et al., 2016; Sterne et al., 2019) were used to assess the potential for bias across all studies included in our meta‐analysis.^3^ We merged and adapted these items to provide a uniform assessment of risk of bias across all included studies, and because we did not consider many of the baseline questions to be relevant to this body of research. Our modified items included: (A) Whether assignment to groups was random, (B) Whether there were baseline differences between groups that were unaccounted for by the analysis, (C) Whether an appropriate analysis was used to control for any potential confounding variables, (D) Whether there were any failures in the implementation of the intervention that were likely to affect the results, (E) Whether there was reason to expect bias in the data used to evaluate the intervention, and (F) Whether the researchers were able to establish proper temporal ordering between the treatment and the outcome. Randomization was a dichotomous response (No/Yes), but all other questions were rated as either “No,” “Probably no,” “Probably yes,” “Yes,” or “No information.” It is important to note here that these ratings, while assessed in duplicate, do involve an inherent element of subjectivity. Additionally, these ratings correspond only to our outcomes of interest and the analyses from which we were able to calculate an effect size. At times, these analyses are not the primary ones reported by study authors or the primary purposes of the article.

Nonequivalence between groups (item B) was coded “probably yes” or “yes” if there was evidence of important baseline differences between groups that were not controlled for statistically. Otherwise, this item was coded as “probably no.” The appropriateness of the statistical analysis (item C) was coded as “probably yes” for quasi‐experimental studies using multiple regression or ANCOVA models, and “yes” for quasi‐experimental studies using strong statistical matching procedures (e.g., propensity score matching). Quasi‐experimental studies that did not control for confounding factors were rated as “probably no” or “no” for this measure. For experimental studies, the appropriateness of the analysis was coded as “yes” so long as a statistical significance test was used that did not appear to violate any necessary distributional assumptions (e.g., normality, independence). Implementation failures and data missingness (items D and E) were coded as “no” if there was high program fidelity and no evidence of missing data. Similarly, this measure was coded as “probably no” if there was no evidence that implementation issues or data missingness favored one group over the other. Finally, the ability of researchers to establish temporal ordering (item F) was coded as “no” or “probably no” for cross‐sectional studies (i.e., cross‐sectional surveys), and “probably yes” for longitudinal studies. Only longitudinal studies that could definitively separate the intervention and the outcome in time were coded as “yes” on this measure.

At the study‐level, place‐based quasi‐experiments reporting evidence of uncontrolled baseline differences between groups were rated as “high risk” of bias. Quasi‐experimental studies that reported either no evidence of baseline differences between groups or that statistically controlled for baseline differences were rated as “some concerns”. Only place‐based studies using random assignment were rated as “low risk” of bias, so long as the authors did not report evidence of significant issues with the assignment process, analysis, or program implementation.

For person‐based studies, any study coded as “No” or “Probably no” on our temporal ordering measure was rated as “high risk” of bias (i.e., cross‐sectional studies). Studies coded as either “Yes” or “Probably yes” on our temporal ordering measure were rated as “some concerns,” so long as these studies used analytic methods that controlled for possible confounding variables (i.e., longitudinal studies using multiple regression analyses). Only longitudinal studies using strong statistical matching techniques (e.g., propensity score matching) with clear separation of treatment and outcome measures across time were rated as “low risk” of bias for person‐based studies.

#### 4.3.4 Measures of treatment effect

The protocol for this review outlines the anticipated approach for effect size calculations based on the expected nature of the outcome measurements (Weisburd et al., 2021). This section provides a precise outline of our effect size calculations based on the studies included in the review.

Measures of treatment effect varied considerably across outcome groupings. For eligible place‐based studies, effect sizes were calculated using logged relative incident rate ratios (RIRR). These studies predominately reported count data for treatment and control groups during pre‐ and post‐intervention periods (or during post‐intervention periods alone). Given that Cohen's *d* effect sizes are sensitive to the way in which counts are divided across time and space, Wilson (2022) suggests the use of the RIRR for place‐based studies. The RIRR is a difference‐in‐difference effect size that can be expressed using the following equation:

$$\ln\left(\text{RIRR}\right)=\ln\left[\frac{{(x}_{11}/t_{11})(x_{00}/t_{00})}{{(x}_{01}/t_{01})(x_{10}/t_{10})}\right],$$

where the first subscript denotes treatment (1) or control (0) groups and the second subscript denotes post‐intervention (1) or pre‐intervention (0) time periods. The *t*_i_ terms represent the sampling frames and drop out of the equation when the samples are equal or constant across time periods (see Wilson, 2022). Assuming a lack of overdispersion in the outcome measure, the variance of the logged RIRR is calculated using the pre/post counts for each group and time period as follows:

$$v_{\ln(\mathrm{RIRR})}=\frac{1}{x_{11}}+\frac{1}{x_{10}}+\frac{1}{x_{01}}+\frac{1}{x_{00}}.$$

However, given that overdispersion is common in count data (see MacDonald & Lattimore, 2010), an adjustment to the variance is often necessary. Wilson (2022) recommends the following correction for over‐dispersion based on the quasi‐Poisson model:

$$\Phi =\left(\frac{1}{\Sigma_{n_{k}}-4}\right)\Sigma \frac{s_{k}^{2}\left(n_{k}-1\right)}{{\bar{X}}_{k}},$$

where ${\overset{ˉ}{X}}_{k}$ is the average count for treatment and control areas across both pre‐ and post‐intervention time periods, $S_{k}$ is the standard deviation for each average count, and $n_{k}$ is the number of counts (contributing to the mean) for both treatment and control groups across pre‐ and post‐intervention periods. If the Φ value is greater than one, then the variance is multiplied by the Φ value to adjust for overdispersion. Unfortunately, the necessary data to correct for overdispersion was only available in a subset of our eligible place‐based studies. To adjust the variance for the remaining effect sizes, we simply used the mean value of Φ across the studies that presented sufficient data to calculate it.

For most eligible studies of crime and displacement, we were able to calculate an RIRR using reported means or counts. Several studies, however, required alternate methods to obtain an effect size. Two studies reported regression coefficients from count‐based models (MacDonald et al., 2016; McCandless et al., 2016), allowing us to use the logged incident rate ratio and standard error reported directly in the regression model. These regression coefficients also provided estimates that were adjusted for various confounding factors or forms of non‐independence that were possible within the data. One study used a linear probability model to assess the mean difference in probability of a crime occurring for treatment areas/times compared to control areas/times (Weisburd et al., 2016). Here, we used the regression coefficient and the intercept of the regression model to construct a risk ratio. Given that risk ratios can be considered censored counts (see Wilson, 2022), we synthesized this effect size with studies reporting count data. Finally, one study required the use of a digitizing software to obtain numeric data from a line graph comparing treatment and control areas (Murray, 2014). To accomplish this, we used *Engauge Digitizer*, which has been recommended and used in recent meta‐analyses (see No et al., 2018; Tantry et al., 2021).^4^

Mental and physical health outcomes were most frequently reported as dichotomous measures, often using some form of logistic regression. As such, we synthesized these studies using logged odds ratios (ORs). We note here that risk ratios may have been preferable given their ease of interpretation (Weisburd et al., 2022), but we did not often have the requisite data to convert reported ORs into risk ratios. In most cases, we coded ORs directly from logistic regression models and calculated the standard error of the logged OR using the reported 95% confidence interval (CI) (Dennison & Finkeldey, 2021; Hirschtick, 2017; Hirschtick et al., 2020; Jackson, Testa, Vaughn, & Semenza, 2020; Jackson et al., 2021; Lewis & Wu, 2021; Sundaresh et al., 2020; Testa et al., 2021).^5^ However, a subset of eligible studies reported mental health outcomes using continuous or ordinal measurements (Baćak & Apel, 2020; Geller, 2017; Geller et al., 2014; McFarland et al., 2019). For these studies we calculated Hedges' g effect sizes and converted them to logged ORs using the Cox logit method, which multiplies the standardized mean difference by 1.65 and divides the variance by 0.367 (see Sánchez‐Meca et al., 2003; Wilson, 2017).

Individual attitudes toward the police and self‐reported crime/delinquency were generally operationalized as scaled or continuous measurements. We synthesized these studies using Hedges' *g* effect sizes, which represents the standardized mean difference between groups (Hedges, 1981). In many cases, *g* values were calculated from standardized or unstandardized linear regression coefficients (Baćak & Apel, 2021; Rosenbaum et al., 2005; Slocum et al., 2016; Swaner & Brisman, 2014; Wheelock et al., 2019), or path coefficients from structural equation models (Lee et al., 2017; Murray et al., 2021). Other studies reported means and standard deviations (Wiley & Esbensen, 2016; Wiley et al., 2013), *t*‐tests (Tyler et al., 2014), or ordinal frequency distributions from which *g* values could be calculated (Friedman et al., 2004). A small subset of studies examining attitudes toward the police reported ORs or dichotomous frequencies/proportions (Harris & Jones, 2020; Singer, 2013). For these studies, we calculated logged ORs and converted them to Hedges' *g* estimates. Once again, this conversion was done using the Cox logit method, which divides the logged OR by 1.65 and the variance of the logged OR by 1.65^2^ (see Sánchez‐Meca et al., 2003; Wilson, 2017).

All effect sizes were calculated using functions manually built in R statistical software (R Core Team, 2022) based on equations listed in Lum et al. (2020) and Wilson (2017). When applicable, effect sizes were cross‐checked against results from David Wilson's effect size calculator (https://www.campbellcollaboration.org/escalc/html/EffectSizeCalculator-Home.php).

#### 4.3.5 Unit of analysis issues

The unit of analysis for this review was the research study, defined here as each unique or statistically independent sample from which outcomes were drawn. In our main analyses, each study/sample was included only once per outcome grouping. However, we did encounter situations in which a single study reported multiple outcomes from within the same outcome grouping, or where multiple studies reported similar outcomes taken from the same sample of subjects. In these situations, we employed a selection rule to maintain statistical independence between effect sizes included in the same meta‐analytic model. For studies of crime and displacement, we were able to calculate an aggregate effect size for all studies included in the meta‐analysis. Given that these effect sizes utilized all available information, no further selection rule was required. For studies measuring other outcomes, we first attempted to select or calculate the most general/aggregate effect size possible. This led to the selection of general measures such as self‐reported mental or physical health over more specific measures such as sleep problems or functional limitations (Baćak & Apel, 2020; Testa et al., 2021). Where such a selection was not clearly possible, we prioritized the most valid effect size as determined by our risk of bias ratings. For example, a number of studies analyzed similar outcomes taken from the same longitudinal cohort surveys (see e.g., Geller, 2017; Slocum et al., 2016; Turney, 2021; Wiley et al., 2013; Wiley & Esbensen, 2016). In these situations, we selected the effect size determined by coders as being the best causal estimate, or the estimate that did the best job of establishing the elements of causality. In general, this criterion prioritized the selection of well‐matched or adjusted estimates over unmatched or unadjusted estimates.

At times, however, our selection of effect sizes was subjective or arbitrary. To ensure that these selections did not bias the results of our review, we conducted sensitivity analyses that incorporated all calculated effect sizes for each study/sample. These analyses were conducted using robust variance estimation (RVE), which is a method capable of analyzing statistically dependent data structures in meta‐analysis (see Tanner‐Smith et al., 2016). In the RVE model, the weight of each effect size is no longer directly related to its variance. Assuming a correlated data structure, the effect size weights in RVE models become the product of the average effect size within each grouping unit and the number of effect sizes nested within that grouping unit (see Tanner‐Smith et al., 2016). Thus, the weight of each effect size within a study or sample will display an inverse relationship with the number of effect sizes nested within that study or sample. Additionally, all effect sizes within a grouping unit will receive the same weight. This method avoids potential issues associated with the over‐representation of a sample or study due to the inclusion of multiple effect sizes. For our analyses, we assumed a correlated data structure and clustered standard errors by each unique sample (for a similar approach see Wilson et al., 2021).

#### 4.3.6 Dealing with missing data

When studies that were otherwise eligible did not report the necessary data to calculate an effect size, we attempted to contact study authors. Ultimately, we were unable to calculate an effect size for only one eligible study that otherwise would have been included in a meta‐analytic model (Alderden et al., 2011). We review the narrative results of this study and all other eligible studies not included in our meta‐analysis in subsequent sections.

#### 4.3.7 Assessment of heterogeneity

We assessed heterogeneity in effect sizes estimates using the *Q* statistic, *I* ^2^ values, and *τ* ^2^ values. Here, the *Q* statistic represents the statistical significance of the between‐study variance (i.e., whether there is more variance than would be expected from sampling error alone), the *I* ^2^ value represents the percentage of total variance attributable to variance between studies, and the *τ* ^2^ value represents the magnitude of the random‐effects variance component (see Borenstein et al., 2010; Higgins & Thompson, 2002). Additionally, we explored between‐study heterogeneity using various moderator analyses (see Section 4.3.10).

#### 4.3.8 Assessment of reporting biases

Three methods were used to assess the potential for reporting bias. First, we conducted moderator analyses comparing the mean effect sizes for published and unpublished studies. Second, we generated funnel plots with trim‐and‐fill analyses to identify any asymmetries in effect size estimates across standard error values and to impute missing values if needed (Duval & Tweedie, 2000). Finally, we conducted Egger's regression tests to assess the linear relationship between standard error and effect size magnitude (Egger et al., 1997).

#### 4.3.9 Data synthesis

Data synthesis for this review involved standard inverse‐variance weighted meta‐analysis. A separate model was estimated for each unique outcome construct and all outcomes were analyzed using random effects models. The random effects variance component (*τ* ^2^) for each model was derived using restricted maximum likelihood estimation. These primary analyses were conducted in R statistical software using the *metafor* package (Viechtbauer, 2010). Sensitivity models incorporating all calculated effect sizes were estimated using the robu() function found in the *robumeta* package in R statistical software (Fisher & Tipton, 2015).

#### 4.3.10 Subgroup analysis and investigation of heterogeneity

Per the protocol for this review (Weisburd et al., 2021), we investigated heterogeneity across effect size estimates using a variety of additional moderator analyses. Due to the characteristics of our eligible studies and the data that was frequently reported, the moderators used for each outcome grouping differ from those listed in the protocol. For place‐based studies these moderators included:

- Research design (“matched” vs. “unmatched” designs)
- Geographic size (micro place vs. neighborhood/police beat vs. district/precinct vs. entire city)
- Geographic location (US vs. Europe)

For studies assessing mental health outcomes, these moderators included:

- Research design (“adjusted” vs. “unadjusted” estimates)
- Sample demographics (youth sample vs. adult sample)
- Geographic location (US vs. Europe)

For studies assessing individual attitudes toward the police, these moderators included:

- Research design (“adjusted” vs. “unadjusted” estimates)
- Sample demographics (youth sample vs. adult sample)
- Geographic location (US vs. Europe)

We did not employ moderator analyses for physical health outcomes or self‐reported crime/delinquency, given the small number of studies included in these models.

#### 4.3.11 Sensitivity analysis

In addition to the RVE models previously described (see Section 4.3.5), several sensitivity analyses were conducted. One study measuring attitudes toward the police produced a large effect size that was an apparent outlier in the forest plot for this outcome (Singer, 2013). As such, we re‐estimated this model excluding this effect size from the analysis. Additionally, several studies reported measures of police stops that compared only individuals who experienced unfair, false, or dissatisfying stops to those without direct stop experience (Baćak & Apel, 2021; Baćak & Nowotny, 2020; Dennison & Finkeldey, 2021; Lee et al., 2017; Testa et al., 2021). Given the potential for the qualitative nature of the stop experience to be an important moderating factor (see Harris & Jones, 2020; Mazerolle et al., 2013; Slocum et al., 2016), we re‐estimated all applicable models while excluding these studies from the analysis.^6^

### 4.4 Deviations from protocol

In the protocol for this review (Weisburd et al., 2021), we indicated that we would explore differences in effect sizes by racial/ethnic composition and by crime type of focus (e.g., violent vs. drug crime). Unfortunately, too few studies for any specific outcome measure provided separate effect size estimates for racial or ethnic categories. More commonly presented was the demographic and ethnic composition of treatment and control groups in terms of group proportions or percentages. We considered using these data to construct a measure of relative racial difference for treatment groups compared to control groups for each study, and then employing this measure as a continuous independent variable in a meta‐regression. However, in nearly all cases, researchers controlled for the effect of race/ethnicity during their analyses. Thus, using racial composition as a moderator to explain effect sizes that are already adjusted for the effect of race and ethnicity may fail to find a significant relationship for artificial reasons. In addition, few studies within any given outcome grouping provided information on the racial composition of both treatment and control groups, and there was often little variability in these racial compositions, with treatment samples primarily represented by individuals that belonged to a minority group. Regarding the crime type of analysis, all eligible studies presented either a single measure of crime, an aggregate measure of violent crime, or an overall aggregate measure of crime. In other words, there was little consistent variation in terms of the types of crime analyzed (e.g., few studies measured property crime or disorder).

The initial inclusion criteria for this review suggested that eligible interventions must be targeted at a geographic area. However, we identified a considerable number of studies measuring the effect of pedestrian stops on individuals. These studies do not often focus on police intent or provide information suggestive of any specific geographic policing intervention, and thus we expanded our inclusion criteria from what was originally described to include these studies. Finally, there were several outcome measures mentioned in the initial protocol that we were unable to analyze due to a lack of eligible studies (violence in police‐citizen encounters, officer misbehavior, fear of crime, etc.).^7^ Studies measuring community attitudes toward the police (i.e., attitudes of individuals residing in targeted areas who were not directly subject to a police stop) were rare and there was considerable variation in the specific measures used across studies. As such, we were unable to consistently generate appropriate effect size estimates and chose instead to review these results narratively. However, results of meta‐analytic models are presented for all other listed outcomes.

Our protocol also stated that risk of bias ratings would be determined using the Cochrane risk of bias tools (J. A. Sterne et al., 2016; J. A. C. Sterne et al., 2019). Although the items we used to assess risk of bias were adapted from these tools, we did not attempt to utilize them in their entirety or strictly follow the logic laid out by these tools. While this may present concern over replicability, deviations from this approach were necessary to tailor our items to the issues most relevant to this body of research. We detail the logic of our risk of bias ratings in Section 4.3.3.

## 5 RESULTS

### 5.1 Description of studies

#### 5.1.1 Results of the search

**Figure 1:**
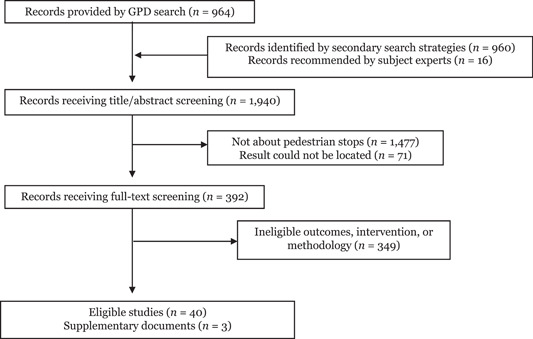
PRISMA flowchart for search results

As seen in Figure 1, our systematic search strategies yielded a total of 1,940 results published between 1970 and 2021. Of these, 964 were provided by the GPD, 960 were identified by our secondary search strategies, and 16 were recommended by subject matter experts.^8^ After screening out titles/abstracts that were clearly not evaluations of pedestrian stops, we were left with 392 results. Full‐text screening of these results yielded 40 eligible studies and 3 supplementary reports associated with these studies. Descriptive statistics for our eligible studies are displayed in Table 1.

**Table 1:** Aggregate study characteristics

Characteristic	*N*	%
Country/Region		
United States	33	82.5
Europe	7	17.5
Unit of analysis		
Individuals	29	72.5
Geographic areas	11	27.5
Outcome^a^		
Crime	10	25.0
Mental health	10	25.0
Attitudes toward the police	9	22.5
Self‐reported crime/delinquency	6	15.0
Physical health	5	12.5
Spatial displacement	4	10.0
Other	4	10.0
Research design		
Quasi‐experimental (unmatched)	29	72.5
Quasi‐experimental (matched)	10	25.0
Experimental	1	2.5
Publication type		
Journal article	33	82.5
Government report	4	10.0
Book chapter	1	2.5
Doctoral dissertation	1	2.5
Unpublished working paper	1	2.5

^a^ Outcomes are not mutually exclusive and do not sum to 100. Frequencies are based on the number of reports/publications and do not necessarily represent unique sample by outcome combinations.

#### 5.1.2 Description of included studies

The vast majority of included studies were conducted in the United States, though a handful of studies took place in Europe. Non‐US studies generally occurred in the United Kingdom (five of seven non‐US studies), and two studies involved respondents from multiple European countries including the United Kingdom (Baćak & Apel, 2020; Baćak & Apel, 2021). Nearly three‐quarters of our eligible studies used individuals as the unit of analysis rather than geographic areas. The most common outcomes included crime and disorder, mental health, and attitudes toward the police. While over three‐fourths of our eligible studies were published in peer‐reviewed journals, the methodological rigor of these studies was relatively weak overall, with the majority being classified as unmatched quasi‐experimental designs. Finally, it is important to note here that our search did not yield 40 statistically independent studies. Instead, four survey samples were associated with 15 separate studies, yielding a total of 29 unique samples.

Of the 10 eligible studies measuring crime and disorder outcomes, nine were included in our meta‐analysis (see Table 2).^9^ This collection of studies contained a mixture of proactive policing interventions and retrospective evaluations of natural variation in the use of pedestrian stops. For example, five studies assessed the impact of interventions explicitly manipulating pedestrian stops, sometimes in the context of more general proactive policing interventions within specific areas (Boydstun, 1975; Cohen & Ludwig, 2003; McGarrell et al., 2002; Ratcliffe et al., 2011; Sherman & Rogan, 1995). In the Kansas City gun experiment (Sherman & Rogan, 1995), directed patrols were assigned to one police beat with high baseline levels of violent crime. Officers worked overtime shifts attempting to detect and seize firearms through pedestrian and traffic stops. Similar tactics were used in the Indianapolis directed patrol experiment (McGarrell et al., 2002), where officers in the North district employed a targeted deterrence approach using selective pedestrian and vehicle stops to seize illegal weapons and drugs. Pittsburgh's firearm suppression patrol (FSP) program (Cohen & Ludwig, 2003) assigned an additional patrol team to two high crime patrol zones two nights per week, instructing officers to initiate contacts with pedestrians in public areas through “stop‐and‐talk” (p. 221) activities. The Philadelphia foot patrol experiment (Ratcliffe et al., 2011) was designed to increase police visibility in select high crime police beats, with considerable discretion regarding policing style left to the officers. While the intervention did not focus only on pedestrian stops it resulted in an increase of over 60% in pedestrian stops for treatment areas relative to baseline. Finally, the San Diego field interrogation study completely suspended the use of pedestrian stops in one police beat, while maintaining stops in another, to test the effect of the tactic on crime and community attitudes (Boydstun, 1975). Both the San Diego field interrogation study and the Indianapolis directed patrol experiment also contained multiple intervention arms. In the San Diego study, a separate treatment area received specialized training intended to reduce friction with citizens during stops. In the Indianapolis study, the East target areas (rather than the North target areas) used a less selective approach to gun crime enforcement that was more focused on the broad application of traffic stops. In this review we do not include or discuss the impact of the specialized field training that occurred in San Diego or the East target area intervention in Indianapolis.

**Table 2:** Individual study characteristics

Study name	Location	Unit of analysis	Outcome(s)	Design/Analysis^a^	Sample^b^
Alderden et al. (2011)^c^	Chicago (IL)	Places	Crime	Quasi‐experiment (Multiple regression)	281 police beats
Baćak and Apel (2020)	26 European countries	People	Mental and physical health	Quasi‐experiment (Multiple regression)	51,340 adults (European Social Survey)
Baćak and Apel (2021)	26 European countries	People	Attitudes toward police	Quasi‐experiment (Multiple regression)	51,340 adults (European Social Survey)
Baćak and Nowotny (2020)	United States (Nationally representative survey)	People	Mental health	Quasi‐experiment (Multiple regression)	7747 adults (Add Health Survey)
Boydstun (1975)	San Diego (CA)	Places	Crime	Quasi‐experiment (Matched/similar control areas)	3 police beats
Cohen and Ludwig (2003)	Pittsburgh (PA)	Places	Crime	Quasi‐experiment (Difference‐in‐difference‐in‐differences analysis)	6 police zones
Dennison and Finkeldey (2021)	US (Nationally representative survey)	People	Mental health and self‐reported delinquency	Quasi‐experiment (Propensity matching)	11.785 adults (Add Health Survey)
Friedman et al. (2004)	Chicago (IL)	People	Attitudes toward police	Quasi‐experiment (Unadjusted bivariate analysis)	891 youth high school students
Geller (2017)	20 large US cities	People	Mental health	Quasi‐experiment (Multiple regression with propensity score weighting)	3036 youth (Fragile Families and Child Wellbeing Survey)
Geller et al. (2014)	New York City (NY)	People	Mental health	Quasi‐experiment (Multiple regression with propensity score weighting)	1261 adult men
Harris and Jones (2020)	20 large US cities	People	Attitudes toward police	Quasi‐experiment (Multiple regression)	3444 youth (Fragile Families and Child Wellbeing Survey)
Hirschtick et al. (2020)	Chicago (IL)	People	Mental health	Quasi‐experiment (Multiple regression)	1543 adults
Hofer et al. (2020)^c^	20 large US cities	People	Other	Quasi‐experiment (Analysis of covariance)	2406 youth (Fragile Families and Child Wellbeing Survey)
Jackson, Testa, and Vaughn (2020)^c^	20 large US cities	People	Other	Quasi‐experiment (Multiple regression)	3444 youth (Fragile Families and Child Wellbeing Survey)
Jackson, Testa, Vaughn, and Semenza (2020)	20 large US cities	People	Physical health	Quasi‐experiment (Multiple regression)	3444 youth (Fragile Families and Child Wellbeing Survey)
Jackson et al. (2021)	United Kingdom	People	Mental health	Quasi‐experiment (Multiple regression)	10,345 youth
Kochel and Nouri (2021)^c^	Midwestern US city (anonymous)	People	Other	Quasi‐experiment (Multiple regression)	820 adults
Lee et al. (2017)	Pennsylvania (not specified further)	People	Self‐reported crime/delinquency	Quasi‐experiment (SEM with covariates)	357 adults
Lerman and Weaver (2014)^c^	New York City (NY)	Places	Other	Quasi‐experimental (Matched census block groups)	Unclear
Lewis and Wu (2021)	Anonymous university in southern US	People	Mental health	Quasi‐experimental (Multiple regression)	301 university students
MacDonald et al. (2016)	New York City (NY)	Places	Crime and displacement	Quasi‐experimental (Multiple regression)	8091 census block groups
McCandless et al. (2016)	London (UK)	Places	Crime	Quasi‐experimental (Multiple regression)	32 London boroughs
McFarland et al. (2019)	20 large US cities	People	Physical health	Quasi‐experimental (Multiple regression with propensity score weighting)	3435 youth (Fragile Families and Child Wellbeing Survey)
McGarrell et al. (2002)	Indianapolis (IN)	Places	Crime and displacement	Quasi‐experimental (Matched/similar control areas)	6 police beats
Murray (2014)	Scotland (UK)	Places	Crime	Quasi‐experimental (Comparison high vs. low stop rate police forces)	2 police forces
Murray et al. (2021)	Birmingham and Sheffield (England), Edinburgh and Glasgow (Scotland)	People	Attitudes toward police and self‐reported crime/delinquency	Quasi‐experimental (SEM with covariates)	1918 youth
Ratcliffe et al. (2011)	Philadelphia (PA)	Places	Crime and displacement	Experimental (Block randomization)	120 police foot beats
Rosenbaum et al. (2005)	Chicago (IL)	People	Attitudes toward police	Quasi‐experimental (Multiple regression)	505 adults
Sherman and Rogan (1995)	Kansas City (MO)	Places	Crime and displacement	Quasi‐experimental (Matched/similar control areas)	2 police beats
Singer (2013)	London (U.K.)	People	Attitudes toward police	Quasi‐experiment (Bivariate analyses stratified by race)	2464 males aged 15–34
Slocum et al. (2016)	7 US cities	People	Self‐reported crime/delinquency	Quasi‐experiment (SEM with covariates)	2919 youth (National Evaluation of the Gang Resistance Education and Training program)
Sundaresh et al. (2020)	US (Nationally representative survey)	People	Mental and physical health	Quasi‐experiment (Multiple regression)	2815 adults
Swaner and Brisman (2014)	New York City (NY)	People	Attitudes toward police	Quasi‐experiment (Multiple regression)	133 youth
Testa et al. (2021)	US (Nationally representative survey)	People	Physical health	Quasi‐experiment (Multiple regression)	12,057 adults (Add Health Survey)
Turney (2021)	20 large US cities	People	Mental health	Quasi‐experiment (Multiple regression)	3437 youth (Fragile Families and Child Wellbeing survey)
Tyler et al. (2014)	New York City (NY)	People	Attitudes toward police	Quasi‐experiment (Unadjusted bivariate analysis)	1261 adult males
Weisburd et al. (2016)	New York City (NY)	Places	Crime	Quasi‐experiment (Multiple regression with instrumental variable)	12,617 street segments
Wheelock et al. (2019)	Milwaukee (WI)	People	Attitudes toward police	Quasi‐experiment (Multiple regression)	1405 adults
Wiley and Esbensen (2013)	7 US cities	People	Self‐reported crime/delinquency	Quasi‐experiment (Propensity matching)	2614 youth (National Evaluation of the Gang Resistance Education and Training program)
Wiley et al. (2013)	7 US cities	People	Self‐reported crime/delinquency	Quasi‐experiment (Propensity matching)	2127 youth (National Evaluation of the Gang Resistance Education and Training program)

^a^ Where applicable, these determinations correspond to the estimates used to calculate an effect size, which may differ from the main analyses.

^b^ Represents total number of places/people involved in study and not necessarily sample size used to calculate effect size. Sample sizes may change depending on outcome or comparison used.

^c^ Not included in meta‐analysis.

Several other studies examined the impact of pedestrian stops on crime and disorder through exploitation of natural variation in the use of pedestrian stops by police forces across time and space (MacDonald et al., 2016; McCandless et al., 2016; Murray, 2014; Weisburd et al., 2016). Two studies evaluated the use of pedestrian stops at targeted areas in New York City during the early 21st Century (i.e., Operation Impact). MacDonald et al. (2016) compared monthly crime counts for census block groups within impact zones to monthly crime counts for census block groups in other areas of the city, and Weisburd et al. (2016) evaluated the probability of a crime occurring for areas/weeks in which a pedestrian stop occurred to areas/weeks in which a stop did not occur. McCandless et al. (2016) evaluated the impact of Operation BLUNT (a stop and search initiative used to combat knife crime in London) by comparing monthly crime counts for boroughs that were more heavily targeted by the initiative to those that received less attention. Murray (2014) charted levels of violent crime for two police forces in Scotland both before and after diverging trends in stop patterns began to emerge between the two. Across nearly all geographic studies of crime and disorder, researchers reported aggregate crime outcomes or aggregate violent/gun crime outcomes. Follow‐up durations were generally 1 year or less, though two studies provided multiple years of follow‐up data (McCandless et al., 2016; Murray, 2014). In almost all cases, the outcome evaluation occurred while the intervention was still active, and given the relatively small variance in follow‐up durations, we do not conduct separate analyses of results based on follow‐up length.

Treatment conditions in individual‐level studies were operationalized in numerous ways, though all included some comparison of individuals with direct stop experience to those without direct stop experience. Studies deriving from the Fragile Families and Child Wellbeing survey (FFCWS) measured treatment by asking youth whether they had ever been stopped by police “while on the street, at school, in a car, or some other place” (Jackson, Testa, & Vaughn, 2020, p. 753), and youth in the National Evaluation of the Gang Resistance Education and Training program (GREAT) were asked how many times in the past 6 months they had been stopped by the police for questioning (though this variable was generally dichotomized). In the FFCWS, this measure was often taken during the year 15 wave (i.e., when respondents were roughly 15 years old), and the GREAT survey administered this item during the second/third waves of data collection, when the youth were generally 12 years of age or older (see Wiley & Esbensen, 2016). Adults in the National Longitudinal Study of Adolescent to Adult Health survey (Add Health) were asked about police stop experience at two time points. During wave III (when respondents were 18–26 years old), they were asked whether they had ever been stopped or detained by police (excluding minor traffic violations), and at wave V (when respondents were 34–43 years old) they were asked if they had ever been “unfairly stopped, searched, or questioned by police” (Dennison & Finkeldey, 2021, p. 263). Studies using the Add Health data varied in the measure of police stops that they used, with Testa et al. (2021) using the wave V measure, Baćak and Nowotny using the wave III measure, and Dennison and Finkeldey (2021) providing estimates from both measures. Respondents in the European Social Survey (ESS) were asked whether police in their country had approached them, stopped them, or made contact with them for any reason during the past two years (Baćak & Apel, 2020, 2021). The ESS was cross‐sectional, and respondents were, on average, in their late 40s. Other measures of treatment included simply asked respondents whether and how many times they had been stopped by police (e.g., during their lifetime or the past 12 months, see Hirschtick et al., 2020; Lewis & Wu, 2021; Singer, 2013), whether they had ever been “falsely stopped” (Lee et al., 2017, p. 101) by police, whether they had ever been “stopped and searched by a police officer” (Murray et al., 2021, p. 268), or whether they had been subject to police‐initiated/involuntary contact (Rosenbaum et al., 2005; Wheelock et al., 2019).

Ten individual‐level studies measured mental health outcomes. These outcomes commonly included anxiety (Geller, 2017; Geller et al., 2014), depression (Baćak & Nowotny, 2020; Hirschtick et al., 2020; Turney, 2021), suicidality (Dennison & Finkeldey, 2021; Jackson et al., 2021) and PTSD symptoms (Geller, 2017; Hirschtick et al., 2020; Lewis & Wu, 2021). All such studies used self‐reported questionnaire or interview surveys, often incorporating items from validated medical instruments. Similar procedures were used across the five studies measuring physical health outcomes, which included self‐reported poor health (Baćak & Apel, 2020; McFarland et al., 2019) and sleep problems (Jackson, Testa, Vaughn, & Semenza, 2020; Testa et al., 2021). Attitudes toward the police and self‐reported crime/delinquency were also measured using self‐report surveys and interviews. Common outcomes for attitudes toward the police included scaled or ordinal measures of police legitimacy (Baćak & Apel, 2021; Murray et al., 2021; Tyler et al., 2014), respect (Friedman et al., 2004; Harris & Jones, 2020; Singer, 2013), trust (Friedman et al., 2004; Murray et al., 2021; Singer, 2013), satisfaction (Wheelock et al., 2019), and overall negative attitudes (Rosenbaum et al., 2005; Swaner & Brisman, 2014). For the six studies measuring self‐reported crime/delinquency, these measures were operationalized as composite counts or scales that included multiple forms of adolescent or adult criminality (Slocum et al., 2016; Wiley & Esbensen, 2016; Wiley et al., 2013), drug use (Dennison & Finkeldey, 2021), or general non‐compliance with the law (Murray et al., 2021). Mental/physical health outcomes and attitudes toward the police were generally measured as current or lifetime outcomes, while studies on self‐reported crime/delinquency measured behavior taking place within the prior 6–12 months.

As previously mentioned, there was considerable overlap in the surveys/samples used across person‐based studies. Seven studies analyzed respondents from the FFCWS (Geller, 2017; Harris & Jones, 2020; Hofer et al., 2020; Jackson, Testa, & Vaughn, 2020; Jackson, Testa, Vaughn, & Semenza, 2020; McFarland et al., 2019; Turney, 2021), three studies analyzed respondents from the Add Health survey (Baćak & Nowotny, 2020; Dennison & Finkeldey, 2021; Testa et al., 2021), three studies analyzed respondents from the GREAT survey (Slocum et al., 2016; Wiley & Esbensen, 2016; Wiley et al., 2013), and two studies analyzed respondents from the ESS (Baćak & Apel, 2020, 2021). The FFCWS, Add Health, and GREAT surveys are all longitudinal cohort surveys administered in the United States, while the ESS is a cross‐sectional survey of 26 European countries. Though the survey waves and total sample sizes analyzed across these studies differed, there is still considerable overlap between them. At times, studies using the same sample analyzed conceptually distinct outcomes, such as attitudes toward police (Harris & Jones, 2020) and mental health (Geller, 2017). However, at other times these outcomes were conceptually similar. For example, both Geller (2017) and Turney (2021) used the FFCWS to measure mental health outcomes (anxiety and depressive symptoms, respectively). In these situations, only one study was selected per model (see Section 4.3.5), and accordingly, our main analyses do not generally include the total number of studies for each outcome grouping reported in Table 1. For mental health outcomes, two samples were associated with four studies, resulting in a total of eight unique samples. For physical health outcomes, one sample was associated with two studies, resulting in a total of four unique samples. For self‐reported crime/delinquency, one sample was associated with three studies, resulting in a total of four unique samples. In addition, of the 14 studies that reported outcomes for youth samples, seven of these studies used the FFCWS survey and three used the GREAT survey, resulting in only six unique samples.

Finally, four studies met our inclusion criteria but were too conceptually dissimilar from the studies described above to include in our meta‐analysis. Two studies used self‐report surveys from the FFCWS to measure respondents' degree of legal cynicism (Hofer et al., 2020; Jackson, Testa, & Vaughn, 2020). Here, legal cynicism involved attitudes toward multiple aspects of the legal and criminal justice systems, rather than toward the police alone. Thus, while we considered legal cynicism to be an important outcome, we did not synthesize it with studies measuring attitudes toward the police. One study measured community members perceived sense of safety, comparing individuals who had been stopped by police in the past 6 months to those who had not (Kochel & Nouri, 2021). We considered this outcome analogous to fear of crime (defined as eligible in the protocol for this review), but we did not have enough conceptually similar outcomes to conduct a meta‐analysis. Additionally, one study measured civic engagement using 311 calls to the police by comparing precincts above and below the mean stop rate per capita (Lerman & Weaver, 2014). While 311 calls are not a measure of crime, they may represent a measure of citizen engagement with the legal system. However, the unit of analysis in this study was geographic and we lacked comparable outcomes from our other geographic studies. We review the results of the studies not included in our meta‐analysis in Section 5.5. Additionally, narrative summaries of all eligible studies can be seen in Supporting Information: Appendix E.

#### 5.1.3 Excluded studies

A number of studies published between 1970 and 2021 warranted further discussion during our screening processes but were ultimately determined to be ineligible. These studies were generally deemed ineligible based on measures of treatment that were either too broad (see Bradford, 2017; DeVylder, Frey, et al., 2017; DeVylder, Oh, et al., 2017; Kennedy et al., 2015; Lehrer & Lepage, 2020; McFarland et al., 2018; Rosenfeld et al., 2014; Sargeant et al., 2021; Villaveces et al., 2000) or too specific (Bryant et al., 2015; Ostrom & Whitaker, 1973). For example, we excluded studies that compared individuals who were searched by police to those who were not searched by police (rather than a more general measure of police stops), interventions solely or primarily involving traffic stops or citations, or interventions incorporating larger legislative, enforcement, or community‐based efforts.^10^ Three other studies were excluded based on methodology (Hoover et al., 2016; Jackson et al., 2021; Sewell & Jefferson, 2016). If a direct comparison between people or places experiencing more versus less stop activity could not be constructed, we did not include the study in this review. Of note, we also screened out studies that precluded binary comparisons of treatment and control groups for example studies that measured stops as a continuous or scaled independent variable (e.g., Del Toro et al., 2019; Rosenfeld & Fornango, 2014, 2017; Tiratelli et al., 2018). It was often not possible to calculate effect sizes from these studies or synthesize them with the studies considered eligible for this review.

Finally, several studies published after our 2021 deadline that would have otherwise met our eligibility criteria were recommended by subject matter experts. While we excluded these studies from our meta‐analysis and main results, we discuss the general findings of these studies and their implications for the results of our review in Section 5.5.

### 5.2 Risk of bias in included studies

Our risk of bias ratings for geographic crime and disorder studies can be seen in Table 3. Overall, we considered these studies to be at moderate risk of bias toward treatment. All studies evidenced temporal ordering. Only one study used random assignment (Ratcliffe et al., 2011), and only three others selected control areas based on their comparability to treatment areas (Boydstun, 1975; McGarrell et al., 2002; Sherman & Rogan, 1995). The remaining studies compared treatment areas to the remainder of a jurisdiction or sample not receiving treatment. Of these studies, Weisburd et al. (2016) used a strong instrumental variable approach to account for treatment endogeneity and to reduce potential bias. Often, treatment areas in non‐experimental studies were selected based on high baseline crime rates, increasing the risk of bias toward treatment. However, researchers generally controlled for these baseline differences using multiple regression and/or difference‐in‐difference analyses (see Cohen & Ludwig, 2003; MacDonald et al., 2016; McCandless et al., 2016). Only one study required the calculation of an effect size using unadjusted and unmatched data (Murray, 2014). This study produced an effect size that was largely null, however, and did not appear to be biased toward treatment. Several studies encountered minor issues with data collection or program implementation, such as the redrawing of area boundaries after the start of the intervention (Boydstun, 1975), alternative interventions taking places during the study evaluation period (McGarrell et al., 2002), minor treatment contamination (Ratcliffe et al., 2011), or the suspension of funding during the study period (Sherman & Rogan, 1995), but there was no evidence of any major issues with implementation or data accuracy that were likely to impact study findings.

**Table 3:** Risk of bias ratings for geographic studies

Study	Randomization^a^	Nonequivalence^b^	Appropriate analysis^c^	Implementation failures^d^	Data missingness^e^	Temporal ordering^f^	Rating
Boydstun (1975)	No	Probably no	Probably yes	Probably no	Probably no	Yes	Some concerns
Cohen and Ludwig (2003)	No	Probably no	Probably yes	No information	Probably no	Yes	Some concerns
MacDonald et al. (2016)	No	Probably no	Probably yes	No information	No	Yes	Some concerns
McCandless et al. (2016)	No	Probably no	Probably yes	No information	Probably no	Yes	Some concerns
McGarrell et al. (2002)	No	Probably no	Probably yes	Probably no	No	Yes	Some concerns
Murray (2014)	No	Probably yes	No	No information	Probably no	Yes	High risk
Ratcliffe et al. (2011)	Yes	Probably no	Yes	Probably no	No	Yes	Low risk
Sherman and Rogan (1995)	No	Probably no	Probably yes	Probably no	No	Yes	Some concerns
Weisburd et al. (2016)	No	Probably no	Probably yes	No information	Probably no	Yes	Some concerns

^a^ Was random allocation used?

^b^ Were there potential sources of nonequivalence that were unaccounted for?

^c^ Was an appropriate analysis used to control for confounding domains?

^d^ Were there failures in implementing the intervention that could have affected the outcome?

^e^ Is there reason to expect bias in the data used to analyze the intervention?

^f^ Can the study establish temporal ordering between the treatment and outcome?

Risk of bias ratings for person‐based studies can be seen in Table 4. Here, we do not include an item about implementation failures as there was generally little to no information about the intervention itself (i.e., the police stop). For outcomes involving attitudes toward the police, mental health, and physical health, we consider these studies to be at high risk of bias overall. None of the person‐based studies used random allocation. Many of these studies also identified significant baseline differences in the demographic composition of treatment and control groups, while several other studies did not provide descriptive information to compare the two groups. For example, many studies found that Black and male respondents were more likely to be stopped by police than White and female respondents (see e.g., Dennison & Finkeldey, 2021; Friedman et al., 2004; Geller, 2017; Singer, 2013; Wheelock et al., 2019). Given this, our ratings concerning the appropriateness of the statistical analysis were primarily concerned with the inclusion of these characteristics as covariates. Most person‐based studies analyzed outcomes using various forms of multiple regression that included control variables related to demographic, economic, and/or behavioral differences between groups (see Dennison & Finkeldey, 2021; Geller et al., 2014; Harris & Jones, 2020). However, a subset of studies measuring attitudes toward the police used unadjusted bivariate analyses, presenting considerably higher risk of bias toward control groups (see Friedman et al., 2004; Singer, 2013). There was also concern regarding attrition and/or nonresponse bias across all person‐based studies. However, there was generally no information presented to suggest that attrition or non‐response differed between individuals who were stopped by police and those who were not.

**Table 4:** Risk of bias ratings for person‐based studies

Study	Randomization^a^	Nonequivalence^b^	Appropriate analysis^c^	Data missingness^e^	Temporal ordering^f^	Rating
Baćak and Apel (2020)	No	No information	Probably yes	Probably no	No	High risk
Baćak and Apel (2021)	No	No information	Probably yes	Probably no	No	High risk
Baćak and Nowotny (2020)	No	Probably no	Probably yes	Probably no	No	High risk
Dennison and Finkeldey (2021)	No	Probably no	Yes	Probably no	Probably yes	Some concerns
Friedman et al. (2004)	No	Probably yes	No	Probably no	No	High risk
Geller (2017)	No	Probably no	Yes	Probably no	Probably yes	Some concerns
Geller et al. (2014)	No	No information	Yes	Probably no	No	High risk
Harris and Jones (2020)	No	No information	Probably yes	Probably no	No	High risk
Hirschtick et al. (2020)	No	No information	Probably yes	Probably no	No	High risk
Jackson et al. (2021)	No	Probably no	Probably yes	Probably no	Probably yes	Some concerns
Jackson, Testa, Vaughn, and Semenza (2020)	No	Probably no	Probably yes	Probably no	No	High risk
Lee et al. (2017)	No	No information	Probably yes	Probably no	Yes	Some concerns
Lewis and Wu (2021)	No	No information	Probably yes	Probably no	No	High risk
McFarland et al. (2019)	No	Probably no	Yes	Probably no	Probably yes	Some concerns
Murray et al. (2021)	No	Probably no	Probably yes	Probably no	No	High risk
Rosenbaum et al. (2005)	No	Probably no	Probably yes	Probably no	Yes	Some concerns
Singer (2013)	No	Probably yes	No	No	No	High risk
Slocum et al. (2016)	No	No information	Probably yes	Probably no	Yes	Some concerns
Sundaresh et al. (2020)	No	No information	Probably yes	Probably no	No	High risk
Swaner and Brisman (2014)	No	No information	Probably yes	No	No	High risk
Testa et al. (2021)	No	No information	Probably yes	Probably no	Probably no	High risk
Turney (2021)	No	No information	Probably yes	Probably no	Probably yes	Some concerns
Tyler et al. (2014)	No	No information	No	No	No	High risk
Wheelock et al. (2019)	No	Probably no	Probably yes	Probably no	No	High risk
Wiley and Esbensen (2016)	No	Probably no	Yes	Probably no	Yes	Low risk
Wiley et al. (2013)	No	Probably no	Yes	Probably no	Yes	Low risk

^a^ Was random allocation used?

^b^ Were there potential sources of nonequivalence that were unaccounted for?

^c^ Was an appropriate analysis used to control for confounding domains?

^e^ Is there reason to expect bias in the data used to analyze the intervention?

^f^ Can the study establish temporal ordering between the treatment and outcome?

The most pressing issue facing our collection of person‐based studies involved temporal ordering. Considering that the majority of studies analyzed cross‐sectional data or longitudinal data in which the independent and dependent variables were measured during the same wave of data collection, there was often no clear way to establish the order of these variables across time. While matching subjects on factors that may make them more or less likely to be stopped (or simply controlling for these factors via regression models) helps to reduce this concern, there remains a potential for reverse causality. That is, the presence of mental health issues or negative attitudes toward the police may lead to increased police stops, rather than vice versa. Only one study measuring attitudes toward the police incorporated both a pre‐ and post‐stop outcome measure (Rosenbaum et al., 2005). Additionally, a subset of studies on mental health attempted to address this limitation by incorporating baseline outcome measurements (i.e., measures of mental health taken at prior survey waves, see Dennison & Finkeldey, 2021; Geller, 2017; Turney, 2021). However, with large amounts of time elapsing between survey waves and a lack of knowledge as to when a respondent's police stop occurred, it remains possible that any changes from baseline mental health occurred before the stop experience.

Thus, while we consider studies that incorporate baseline measurements and strong propensity matching techniques to be the most appropriate analyses, we still consider these studies to have potential bias toward control groups. It is unclear how accurately researchers can control for or match groups on their propensity to be stopped by police. Despite several studies taking care to include an array of factors related to prior behavior, beliefs, personal and family characteristics, and neighborhood/area‐level influences on behavior (see e.g., Dennison & Finkeldey, 2021; Geller, 2017; Harris & Jones, 2020; Jackson et al., 2021; Wiley et al., 2013), it may be difficult to control for all salient components of an individual's routine activity patterns that influence their probability of coming into contact with a police officer. Of note, we do consider the risk of bias in studies measuring self‐reported crime/delinquency to be less severe. These studies used multiple waves of data, and by virtue of the outcome measure, were able to separate the independent and dependent variables into separate time periods. For example, Wiley et al. (2013) and Wiley and Esbensen (2016) matched respondents on their propensity to be stopped by police using covariates measured at time 1. They then measured stop experience in the past 6‐months at times 2 and 3, before measuring crime/delinquency outcomes for the 6‐month period following the police stop measure. Aside from these isolated examples, however, the strength of causal inferences across our person‐based studies is limited.

### 5.3 Effects of the intervention

In total, we analyzed 58 effect sizes across six outcome groupings (including sensitivity analyses), representing 90,904 people and 20,876 places. The summary effect sizes for each outcome can be seen in Table 5 along with 95% CIs and heterogeneity statistics. For logged RIRR and OR effect sizes, we present the anti‐logarithm of the summary effect size for ease of interpretation. Here, values greater than 1 indicate an increase in incidence (for RIRR values) or odds (for OR values), and values less than 1 indicate a decrease in incidence or odds for treatment groups relative to control groups. As shown in Table 5, our analyses detect significant relationships between pedestrian stops and all outcome measures, suggesting both intended and unintended effects of the intervention. In the following sections we present forest plots for each outcome and interpret our findings further.

**Table 5:** Summary effect sizes and related statistics

Outcome	Effect size	95% CI	*Q*	*I* ^2^	τ^2^	*k*
Crime and disorder	RIRR = 0.87***	0.84, 0.91	11.62	13.43%	0.001	9
Displacement	RIRR = 0.93***	0.91, 0.96	0.98	0.00%	0.00	4
Mental health issues	OR = 1.46***	1.24, 1.72	24.21***	78.07%	0.03	8
Physical health issues	OR = 1.36***	1.14, 1.62	19.74***	78.62%	0.02	4
Attitudes toward police	*g* = −0.38***	−0.59, −0.17	201.14***	97.54%	0.09	9
Self‐report crime/delinquency	*g* = 0.30***	0.12, 0.48	9.21*	73.17%	0.02	4

Abbreviations: CI, confidence interval; *I* ^2^, percentage of variability due to between‐study heterogeneity; *k*, number of effect sizes; OR, odds ratio; *Q*, test for heterogeneity; RIRR, relative incident rate ratios; *τ* ^2^ = random effects variance component.

* *p* < 0.05.

*** *p* < 0.001.

#### 5.3.1 Crime and displacement

**Figure 2:**
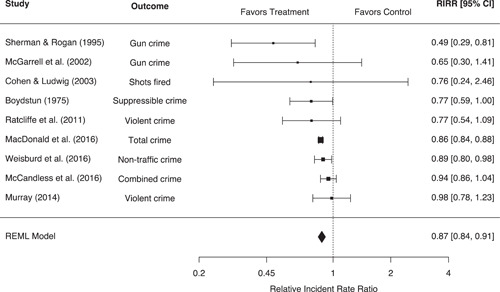
Crime effects for place‐based studies

**Figure 3:**
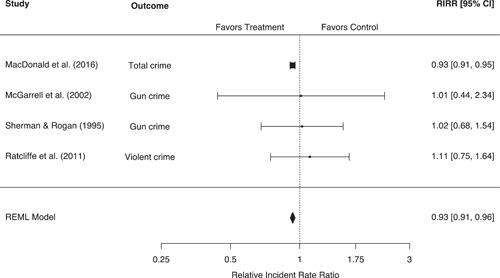
Displacement effects for place‐based studies

Figures 2 and 3 display effect sizes from nine eligible studies of crime/disorder and four studies of spatial displacement following pedestrian stop interventions. All such studies were place‐based and utilized official data sources (e.g., incident reports or calls for service). Effect sizes to the left of the reference line indicate reductions in crime/disorder for treatment areas relative to control areas, and thus are considered effects favorable to treatment. The size of the point estimates in the forest plots represent the weight that each study received in the analysis, which is inversely related to the variance of the effect size. The combined sample size for crime and displacement outcomes was 20,876 and 8,220 geographic areas, respectively.

As seen in Figure 2, pedestrian stops interventions were associated with a statistically significant reduction in crime of 13% (*p* < 0.001) for treatment areas relative to control areas. CIs for this outcome suggest a crime reduction effect ranging from 9% to 16%. There is also a notable lack of heterogeneity in these effect sizes. All effect sizes tend to favor treatment with overlapping CIs, and between‐study heterogeneity was not statistically significant, as indicated by the *Q* statistic.

Figure 3 displays effect sizes for studies measuring spatial displacement. Here, we followed the approach used by Telep et al. (2014) in comparing treatment catchment or buffer areas to control areas when catchment areas were not drawn around the control areas themselves. Results indicate a statistically significant diffusion of crime control benefits. Specifically, police stop interventions were associated with a 7% (*p* < 0.001) decrease in crime for treatment displacement areas relative to control areas, with CIs ranging from a 4% decrease in crime to a 9% decrease in crime. There was also a lack of significant or excess heterogeneity in this model, as indicated by the *Q* and τ^2^ statistics. It is important to note, however, that these results seem to be driven by one study receiving a large amount of weight in the analysis (MacDonald et al., 2016). In fact, three out of the four effect sizes favor displacement rather than diffusion. Given this, and the small number of studies, we urge caution in the interpretation of these findings.

#### 5.3.2 Mental and physical health

**Figure 4:**
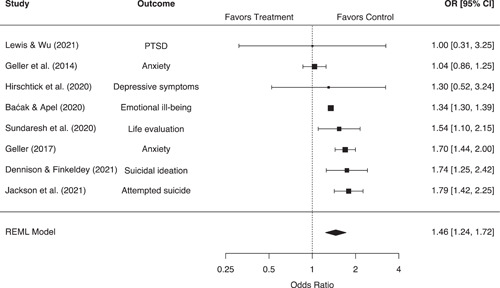
Mental health issues for person‐based studies

**Figure 5:**
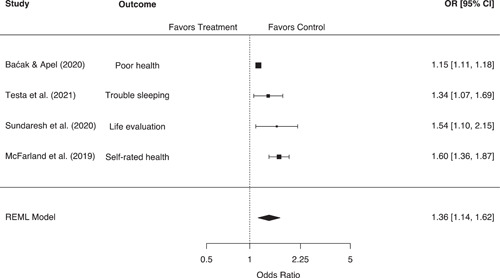
Physical health issues for person‐based studies

Pedestrian stops may be a stressful and traumatizing experience that has negative effects on subjects' mental and physical functioning. Figures 4 and 5 display effect sizes from eight studies measuring mental health issues and four studies measuring physical health issues. Here, effect sizes to the right of the reference line indicate increases in the odds of a mental health issue for treatment groups relative to control groups, and these effects are considered favorable to control groups. Sundaresh et al. (2020) was included in both mental and physical health models given that their outcome (life evaluation) incorporated measures of both mental and physical health. The effect size for this study was also reverse coded so that effects favorable to control moved in the same direction across all studies. The combined sample size for mental and physical health outcomes was 71,810 and 64,898, respectively.

The eight effect sizes shown in Figure 4 suggest that individuals stopped by police were associated with a statistically significant 46% (*p* < 0.001) increase in the odds of a mental health issue, with CIs ranging from a 24% increase to a 72% increase. All effect sizes favored control groups, though there was significant heterogeneity in effect sizes estimates, as roughly 78% of the total variance could be attributed to between‐study variance.

As seen in Figure 5, the four studies measuring physical health outcomes provided similar results. Overall, there was a statistically significant 36% (*p* < 0.001) increase in the odds of a physical health issue for treatment groups relative to control groups, and the CI for this outcome suggests that likely effects range from a 14‐62% increase. All four studies showed significant effects favoring control, though there remains statistically significant between‐study heterogeneity. Despite the strong and significant backfire effects indicated by these mental and physical health analyses, it is important to reiterate the inherent difficulties and potential biases involved in measuring these outcomes. Causal interpretations should be made cautiously.

#### 5.3.3 Attitudes toward the police

**Figure 6:**
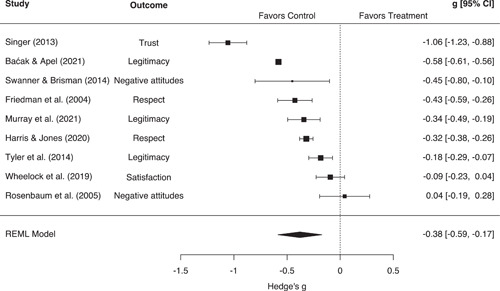
Attitudes toward the police for person‐based studies

Individuals subjected to pedestrian stops, particularly those that are perceived as false or unfair, may harbor resentment and negative future attitudes toward the police. Our nine eligible studies measuring attitudes toward the police are displayed in Figure 6. Hedges' *g* effect sizes were used for these outcomes given their often scaled or continuous nature. Thus, effect sizes to the left of the reference line indicate worsening attitudes toward the police and are defined as effects favorable to control. Several effect sizes were reverse coded to ensure that negative values corresponded to worsening attitudes for all studies (Rosenbaum et al., 2005; Swaner & Brisman, 2014). The combined sample size for this outcome was 41,423.

Results from Figure 6 indicate that pedestrians stops were associated with a statistically significant small to moderate decrease in attitudes favorable to the police (*g* = −0.38, 95% CI: −0.59, −0.17, *p* < 0.001). The classification of this effect size as small to moderate is based on the conventions suggested by Cohen (1992), however, outside of laboratory settings this effect may be considered rather large (Lipsey et al., 2012). Using the binomial effect size display to convert this effect into a percentage point difference suggests an 18.6% differential between control and treatment groups.^11^ Eight of nine effect sizes for this outcome favored control groups, however, there is also a very large degree of between study variance. Over 97% of the total heterogeneity in this model can be attributed to heterogeneity between studies, and one study (Singer, 2013) displayed an unusually large effect size (which we return to in our sensitivity analyses). Once again, while this evidence implies a strong backfire effect of pedestrian stops on attitudes toward the police, the risk of bias toward control groups across these studies is generally high. Additionally, this level of heterogeneity suggests a large degree of uncertainty as to the true mean effect size.

#### 5.3.4 Self‐reported crime/delinquency

**Figure 7:**
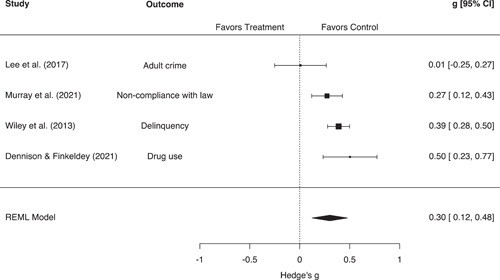
Self‐reported crime/delinquency for person‐based studies

If pedestrian stops result in the imposition of a formal label that leads to the exclusion of individuals from conventional bonds and activities, then we may also expect to see a backfire effect in terms of specific deterrence. Results from the four eligible studies comparing self‐reported crime/delinquency for individuals stopped by police to individuals not stopped by police are shown in Figure 7. Here, effects to the right of the no reference line indicate increases in self‐reported crime/delinquency for treatment groups relative to control groups, and thus are defined as effects favorable to control. The combined sample size for this outcome was 11,402.

Results from this analysis continue to suggest deleterious individual‐level effects of pedestrian stops. Specifically, there was a statistically significant increase in self‐reported crime/delinquency for treatment groups relative to control groups (*g* = 0.30, 95% CI: 0.12, 0.48, *p* < 0.001). Using the binomial effect size display to convert this effect into a percentage point difference suggests an approximate 15% differential between control and treatment groups. All four effect sizes reported here favored control, though there remains a statistically significant amount of between‐study heterogeneity.

#### 5.3.5 Violence in police‐citizen encounters

We did not locate any eligible studies providing measures of violence in police‐citizen encounters.

#### 5.3.6 Officer misbehavior

Only one eligible study provided a potential measure of officer misbehavior. The San Diego field interrogation experiment measured citizen complaints against the police both before and after the intervention, however, there were no complaints during either time period (Boydstun, 1975).

#### 5.3.7 Sensitivity analyses

We conducted several robustness checks to assess the sensitivity of our results to different specifications. As previously noted, our main models included a selection of one effect size per study/sample. At times, this selection of effect sizes could be considered arbitrary, which presents concern over the potential for these selections to bias our results. Thus, we conducted sensitivity analyses using RVE that incorporated all calculated effect sizes taken from each sample and outcome grouping. These models were only estimated for mental health outcomes, attitudes toward the police, and self‐reported crime/delinquency as these were the only outcomes for which difficult effect size selections were often required. For each model, standard errors were clustered by sample, resulting in eight unique clusters for mental health outcomes, nine clusters for attitudes toward the police, and four clusters for self‐reported crime/delinquency.

Results from our RVE models are displayed in Table 6. For mental health issues and attitudes toward the police, RVE models continued to suggest a statistically significant effect favorable to control groups. The mean effect size for mental health studies decreased slightly (from a 46% increase in our main specification to a 37% increase in the RVE model), while the mean effect size for attitudes toward the police increased slightly (from *g* = −0.38 in our main specification to *g* = −0.40 in the RVE model). Results for self‐reported crime/delinquency remained similar in magnitude (from *g* = 0.30 in our main specification to *g* = 0.26 in the RVE model), but these results were no longer significant at a 0.05 threshold. However, the degrees of freedom for this model were fewer than four, which is considered an unreliable sample size for RVE estimation (see Tanner‐Smith et al., 2016).

**Table 6:** Robust variance estimation models

Outcome	Effect size	95% CI	*p* Value	*I* ^2^	*τ* ^2^	*k*
Mental health	OR = 1.37**	1.14, 1.65	0.01	78.85%	0.03	19
Attitudes toward police	*g* = −0.40*	−0.71, −0.10	0.02	98.14%	0.18	14
Self‐report crime	*g* = 0.26^+^	−0.02, 0.54	0.06	80.67%	0.04	8

Abbreviations: CI, confidence interval; *I* ^2^, percentage of variability due to between‐study heterogeneity; *k*, number of effect sizes; OR, odds ratio; *τ* ^2^ = random effects variance component.

*p < 0.05.

**p < 0.01.

^+^ *p* < 0.10.

Our main model specification for attitudes toward the police also suggested the presence of an outlier (Singer, 2013). Thus, we reanalyzed this model while excluding this study from the analysis. Results continued to indicate a statistically significant decrease in attitudes toward the police for treatment groups relative to control groups (*g* = −0.30, 95% CI [−0.44, −0.16]). Moreover, this effect was similar in magnitude to that of the original specification (*g* = −0.38, 95% CI [−0.59, −0.17]). Finally, one study measuring attitudes toward the police (Baćak & Apel, 2021), two studies measuring mental health issues (Baćak & Nowotny, 2020; Dennison & Finkeldey, 2021), and one study measuring self‐reported crime/delinquency (Lee et al., 2017) used measures of police stops that were limited to unfair, false, or unsatisfactory stop experiences.^12^ Given that these experiences may have separate impacts on effect sizes, we re‐estimated each model while excluding these studies. Results of these models were nearly identical to those of our main specifications (not shown here).

### 5.4 Subgroup analyses

The examination of effect size moderators provides important context to the interpretation of meta‐analytic findings (see Johnson et al., 2015). As such, we explore several factors that may moderator treatment effects across each of our outcome groupings. While many systematic reviews of crime and justice interventions compare effect sizes for experimental and quasi‐experimental studies (e.g., Braga et al., 2019; Hinkle et al., 2020), we lacked a sufficient number of randomized experiments to conduct such an analysis. Thus, to assess the effect of risk of bias on study findings, we compare effect sizes for “matched” and “unmatched” designs (for crime/disorder and mental health outcomes) and for “adjusted” and “unadjusted” designs (for attitudes toward the police). Other moderators include the geographic size of the targeted areas (for crime/disorder outcomes), youth versus adult samples (for mental health outcomes and attitudes toward the police), and the geographic location of the study (for all outcomes). Moderator analyses are not conducted for spatial displacement, physical health, or self‐reported crime/delinquency given the small number of studies included in these models. Categorical moderator analyses were conducted using the analog to the ANOVA method (Lipsey & Wilson) and continuous moderator analyses were conducted using meta‐regression (Higgins et al., 2020).

#### 5.4.1 Research design

Studies with weaker methodological rigor have been shown to produce larger effect size estimates than those with stronger methodological rigor (Weisburd et al., 2001). To test the potential for methodological strength to impact our crime/disorder and mental health findings, we compared effect sizes for studies with matched versus unmatched designs. Here, “matched” does not necessarily indicate a statistical matching procedure, but rather any attempt to identify comparable control areas.^13^

Results of these moderator analyses can be seen in Table 7. For crime and disorder outcomes, unmatched designs were associated with a 10% decrease in crime for treatment areas relative to control areas, while matched designs were associated with a 19% decrease. This difference was non‐significant and both effect sizes remained statistically significant individually (as indicated by the 95% CIs). Of note, if we consider Weisburd et al. (2016) to be an unmatched design, the difference between matched and unmatched effect sizes increases in magnitude and becomes statistically significant. However, we find this distinction to be misleading as Weisburd et al. used an instrumental variable approach that is likely stronger than any of the non‐statistical matching procedures used in our other studies. For mental health outcomes, unmatched studies were associated with a 49% increase in the odds of a mental health issue for treatment groups relative to control groups, while matched designs were associated with a 43% increase. Once again, this difference was non‐significant and both effect sizes remained statistically significant individually, with 95% CIs greater than one.

**Table 7:** Matched versus unmatched comparison groups

Outcome	Level	*k*	Effect size	95% CI	*Q*_model_ (*p* value)
Crime	Unmatched	4	RIRR = 0.90	0.82, 0.995	1.80 (*p* = 0.13)
	Matched	5	RIRR = 0.81	0.71, 0.92	
Mental health	Unmatched	5	OR = 1.49	1.16, 1.90	0.04 (*p* = 0.83)
	Matched	3	OR = 1.43	1.10, 1.86	

*Note*: *Q*_model_ tests whether a significant amount of heterogeneity is explained by the moderator.

Abbreviations: CI, confidence interval; OR, odds ratio; RIRR, relative incident rate ratios.

No eligible studies for attitudes toward the police employed matching procedures. However, several studies provided only unadjusted bivariate data from which an effect size could be calculated (Friedman et al., 2004; Singer, 2013; Tyler et al., 2014). Thus, to assess risk of bias for these studies we compared effect sizes for adjusted and unadjusted estimates. Results from this analysis can be seen in Table 8. While adjusted effect sizes were notably smaller than unadjusted effect sizes, by an average of *g* = 0.26 (95% CI [−0.17, 0.68]), this difference was not statistically significant and both categories of studies remained significantly different from 0.

**Table 8:** Adjusted versus unadjusted estimates (attitudes toward the police)

Outcome	Level	*k*	Effect size	95% CI	*Q*_model_ (*p* value)
Attitudes toward police	Unadjusted	3	*g* = −0.55	−0.90, −0.20	1.36 (*p* = 0.24)
	Adjusted	6	*g* = −0.29	−0.54, −0.04	

*Note*: *Q*_model_ tests whether a significant amount of heterogeneity is explained by the moderator.

Abbreviation: CI, confidence interval.

#### 5.4.2 Size of geographic area

Weisburd et al. (2014) suggest that the use of pedestrian stops is often targeted at high crime microgeographic areas. If so, then the mere increase in police presence within hot spots of crime and disorder may be responsible for any observed crime reduction effect (see Braga et al., 2019). To test for this potential, we conducted a moderator analysis comparing effect sizes for studies targeting micro‐geographic areas, neighborhoods/police beats, police districts/precincts, and macro‐geographic areas (e.g., entire cities). Given the small number of studies within each of these categories, we treat geographic size as a continuous variable and estimate this moderator analysis as a meta‐regression. Results of this analysis are shown in Table 9. On average, increases in the size of the geographic area targeted led to decreases of between 3% and 4% in effect size estimates (i.e., larger areas received smaller crime reduction benefits), however, this linear effect was not statistically significant (RIRR = 1.04, 95% CI [0.977, 1.105]). Of note, the mean effect sizes for all groups other than macro‐geographic areas displayed CIs less than one, indicating statistical significance. However, we urge caution when interpreting these effects, given the small number of studies in each grouping.

**Table 9:** Size of geographic area (crime and disorder studies)

Outcome	Level	*k*	Effect size	95% CI	Regression coefficient (*p* value)
Geographic size	Micro	1	RIRR = 0.84	0.78, 0.90	0.04 (*p* = .23)
	Neighborhood	4	RIRR = 0.87	0.84, 0.90	
	District/precinct	3	RIRR = 0.90	0.84, 0.96	
	Macro	1	RIRR = 0.94	0.83, 1.06	

*Note*: *Q*_model_ tests whether a significant amount of heterogeneity is explained by the moderator.

Abbreviations: CI, confidence interval; RIRR, relative incident rate ratios.

#### 5.4.3 Youth versus adult samples

Concern regarding the deleterious impact of pedestrian stops is particularly relevant for adolescents, as these populations may be increasingly vulnerable to stressful/traumatic experiences and the imposition of formal labels (Geller, 2017; Jackson et al., 2021; Wiley & Esbensen, 2016). For mental health outcomes and attitudes toward the police, there was sufficient variation in the samples used to compare the effects of pedestrian stops for youth and adults. The results of this analysis can be seen in Table 10.

**Table 10:** Youth versus adult samples

Outcome	Level	*k*	Effect size	95% CI	*Q*_model_ (*p* value)
Mental health	Adult	6	OR = 1.32	1.13, 1.55	3.83+ (*p* = 0.05)
	Youth	2	OR = 1.74	1.39, 2.17	
Attitudes toward police	Adult	5	*g* = −0.38	−0.67, −0.08	0.00 (*p* = 0.99)
	Youth	4	*g* = −0.38	−0.72, −0.04	

*Note*: *Q*_model_ tests whether a significant amount of heterogeneity is explained by the moderator.

Abbreviations: CI, confidence interval; OR, odds ratios.

^+^ *p* < 0.10.

For mental health outcomes, youth samples were associated with a 74% increase in the odds of a mental health issue for treatment groups relative to control groups, while adult samples were associated with only a 32% increase. This difference was nearly statistically significant at the 0.05 level (*p* = 0.0504), suggesting that police stops may have particularly salient effects on the mental health of youth. For attitudes toward the police, there was essentially no difference in mean effect sizes between youth and adult samples (*g* = −0.002, 95% CI [−0.45, 0.44]).

#### 5.4.4 Geographic location

Per the protocol for this review, we also examined the difference in mean effect sizes by geographic location. Given that several studies used samples from multiple countries, we chose to compare studies conducted in the US and Europe. Table 11 displays the results from these analyses. For crime and disorder outcomes, US studies were associated with a statistically significant 9% larger decrease in crime relative to European studies. Individually, US studies were associated with a statistically significant 14% decrease in crime for treatment areas relative to control areas, while European studies were associated with a non‐significant 5% decrease in crime. For mental health outcomes, US studies were associated with a 42% increase in the odds of a mental health issue for treatment groups relative to control groups, while European studies were associated with a 52% increase in these odds. However, this difference was not statistically significant. Finally, for attitudes toward the police, US studies were associated with a significantly smaller mean effect size compared to European studies (mean difference of *g* = 0.42, 95% CI [0.08, 0.76]). While this suggests that pedestrian stops in European settings may impact attitudes toward the police significantly more than in US contexts, it is important to note that the mean effect sizes for both locations suggested statistically significant negative effects. Additionally, all moderator analyses were limited by a small number of studies.

**Table 11:** US versus European studies

Outcome	Level	*k*	Effect size	95% CI	*Q*_model_ (*p* value)
Crime	Europe	3	RIRR = 0.95	0.87, 1.04	4.67* (*p* = 0.03)
	US	7	RIRR = 0.86	0.84, 0.88	
Mental health	Europe	2	OR = 1.52	1.12, 2.06	0.11 (*p* = 0.74)
	US	6	OR = 1.42	1.14, 1.79	
Attitudes toward the police	Europe	3	*g* = −0.65	−0.92, −0.38	5.92* (*p* = 0.02)
	US	6	*g* = −0.23	−0.43, −0.03	

*Note*: *Q*_model_ tests whether a significant amount of heterogeneity is explained by the moderator.

Abbreviations: CI, confidence interval; OR, odds ratio; RIRR, relative incident rate ratios.

* *p* < 0.05.

### 5.5 Studies not included in meta‐analyses

While the primary objective of this review was to examine the impact of pedestrian stops on crime, the community, and the individuals subjected to stops, several relevant studies and outcomes could not be included in our meta‐analysis. Since the number of these studies was small, we opted to review their results narratively. Our findings overall are consistent with those of the studies meta‐analyzed. That is, pedestrian stops appear to negatively affect individual‐level attitudes toward the police and the legal system while simultaneously producing a general deterrent effect on crime and disorder. However, place‐based studies incorporating community surveys provide additional insight to suggest that the deleterious effects of pedestrian stops may be limited to those directly subject to the intervention, rather than the community more broadly.

#### 5.5.1 Eligible studies not included in meta‐analyses

Five studies published between 1970 and 2021 were identified as eligible for inclusion in this review but were not meta‐analyzed due to issues that prevented the calculation of an effect size or the lack of additional studies with conceptually similar outcome measures. Alderden et al. (2011) evaluated the implementation and efficacy of the Chicago Police Department's Deployment Operations Center process, which identified violent crime hot spots and guided leadership decisions on where to deploy officers to reduce violent crime, focusing on gang, drug, and gun crime. Specifically, the intervention employed directed patrols in which officers actively engaged citizens via street stops, traffic stops, and conducted aggressive ordinance enforcement. Fidelity checks indicated that the Chicago Police Department successfully implemented the Deployment Operations Center process as it was designed, and while results tended to favor a reduction in violent crime for DOC beats relative to non‐DOC beats, the intervention was not responsible for significant reductions in violent crime.

Two eligible studies using the same longitudinal survey sample included an outcome of legal cynicism (Hofer et al., 2020; Jackson, Testa, & Vaughn, 2020). Given that this outcome was operationalized as a composite measure representing attitudes toward the legal system more broadly, we considered it too conceptually distinct to synthesize with studies measuring attitudes toward the police. Both Hofer et al. (2020) and Jackson, Testa, and Vaughn (2020) used data from the age 15 assessment of the FFCWS. Hofer et al. compared levels of legal cynicism for youth who experienced vicarious police contact (defined as having witnessed police stops in the respondents' neighborhood or school) and/or direct police contact (defined as directly experiencing a police stop) to youth who reported never having experienced any form of police contact. Youth who had experienced direct or both direct and vicarious contact with police had higher levels of legal cynicism than youth who only experienced vicarious police contact. Situational factors, such as police using harsh language or frisking the youth during an encounter with police, were associated with higher levels of legal cynicism as well. Similar findings were reported by Jackson et al., suggesting that youth subject to direct police stops develop significantly higher levels of legal cynicism than those who do not directly experience stops. While low self‐control demonstrated a stronger relationship with legal cynicism in Jackson et al.'s study than stop experience itself, direct stop experience remained a significant predictor even with the inclusion of low self‐control as a covariate.

Two studies, conducted by Kochel and Nouri (2021) and Lerman and Weaver (2014), investigated disadvantaged community members' perceptions of the extent and nature of police contact, and how these perceptions impacted feelings of safety and community engagement, respectively. Kochel and Nouri found that residents in the high‐violence neighborhoods surveyed had the highest rate of police stop experience within the last six months, as well as more prevalent experiences with unfair police treatment. However, their analyses failed to find any significant effect of being stopped by police in the past 6 months on feelings of safety. Lerman and Weaver investigated how increased rates of stop‐and‐frisk activity in disadvantaged neighborhoods affected community members' civic engagement. Lerman and Weaver used nonemergency 311 calls as a proxy for engagement, comparing precincts above and below the mean stops per capita. Ultimately, Lerman and Weaver found that “high stop” precincts were associated with significantly more 311 requests, though this finding was attenuated by the proportion of stops that resulted in force.

#### 5.5.2 Community surveys from place‐based studies

Four place‐based studies included community surveys to assess the impact of police activity on community members as a secondary outcome measure. Unfortunately, there was little overlap in the measures used and the data reported across these studies, which prevented us from calculating an appropriate effect size in many cases. Results from these surveys generally demonstrated that community members were supportive of increased police activity that included stops, especially when the intervention was effective at reducing perceived crime in their neighborhoods. For example, Alderden et al. (2011) found that residents, especially middle‐class residents, who perceived a high level of disorder were more supportive of suppression‐oriented policing as it had been implemented by the Chicago Police Department's Deployment Operations Center. Alderden et al. also found that many residents still preferred traditional or community‐oriented policing and that higher levels of support for suppression‐oriented policing were associated with stronger perceptions of police legitimacy.

McGarrell et al. (2002) similarly found that community members surveyed following the Indianapolis Police Department's directed patrol strategy indicated acceptance of the intervention, given the crime reduction outcomes that it produced. Specifically, McGarrell et al. found that support for the Indianapolis Police Department was high overall and not significantly affected by the increase in police activities, that police‐community relations were not harmed as a result of the intervention, and that community members generally reported positive community‐level effects after the treatment period ended. These findings align with Sherman and Rogan's (1995) findings from the Kansas City gun experiment. Target area residents surveyed both before and after the intervention reported being more satisfied with their neighborhood, less fearful of crime, and perceived lower rates of disorder and drug crime compared to residents from the control area (Sherman & Rogan, 1995; see also Shaw, 1995). Finally, Boydstun (1975) also found little evidence to suggest a negative impact of pedestrian stops on community members' attitudes toward the police. Community members in both treatment and control areas felt that field interrogations were a legitimate policing activity and there were few significant changes in resident perceptions between pre‐ and post‐intervention surveys. However, respondents residing in areas where field interrogations were suspended did report significant increases in fear of crime that were not similarly observed for respondents in areas where field interrogations were uninterrupted.

#### 5.5.3 2022 studies

Several studies published after our search cut‐off date of 2021 were recommended to us by subject matter experts. While these studies are not formally included in our review, many of them would otherwise be considered eligible, and thus we considered it important to briefly review their findings. Overall, results from these studies are highly consistent with those produced by our review. Braakmann (2022) reported the results of an increase in stop activity after a 2019 murder in the United Kingdom, where Northumbria Police dramatically increased their level of stop and search operations in streets close to the site of the highly publicized murder. Braakmann (2022) used these natural variations to examine the effect of increased stop and search activity on crime, finding that property, weapon, violent, and drug crime were not significantly impacted by the increase in stop and search operations, but that there was a decline in anti‐social behavior, criminal damage, and public order offenses. Turney et al. (2022) and Testa et al. (2022) analyzed survey data from the FFCWS and Pathways to Desistance studies, respectively, with both finding that personal and vicarious police contact were associated with significant decreases in respondents' future orientation. Foster et al. (2022) used the FFCWS data to study the impact of police stops on attitudes toward the police, finding that direct experience with police stops was associated with significant reductions in respect and confidence in the police. These results varied by race, however, and there was stronger evidence of deleterious effects for Black and Hispanic youth compared to White youth. Lastly, Jackson and Testa (2022) found that police contact was associated with worsening sleep behaviors among respondents from the UK millennium cohort survey.

### 5.6 Publication bias

We tested for the presence of publication bias using several methods, including categorical moderator analyses based on publication status (published vs. unpublished), funnel plots with trim‐and‐fill analyses, and Egger's regression tests. Publication bias was not assessed for displacement, physical health, or self‐reported crime/delinquency, given the small number of studies for these outcomes.

For crime and disorder, there was no significant difference in mean effect sizes for published and unpublished studies (*Q*_model_ = 2.77, *p* = 0.096), though published studies were associated with a 7% larger crime reduction effect, relative to unpublished studies (RIRR = 0.93, 95% CI [0.85, 1.01]). For mental health outcomes, published studies were associated with a 17% smaller increase in the odds of a mental health issue (OR = 0.83, 95% CI [0.53, 1.29]), but this difference was also not statistically significant (*Q*_model_ = 0.67, *p* = .41). No moderator analysis based on publication status was conducted for attitudes toward the police as no eligible studies were unpublished.

**Figure 8:**
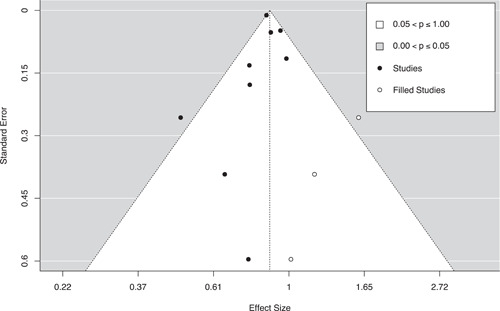
Funnel plot for crime and disorder outcomes

**Figure 9:**
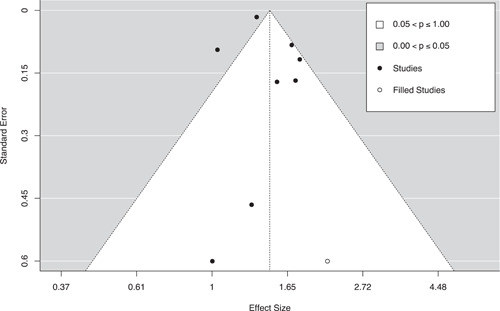
Funnel plot for mental health outcomes

Figure 8 displays the funnel plot and trim‐and‐fill analysis for crime and disorder outcomes. The funnel plot suggests an asymmetry toward the right side of the plot, and the trim‐and‐fill analysis imputed three effect sizes in this direction. However, after the imputation of these effect sizes, the mean effect remained statistically significant and highly similar in magnitude (RIRR = 0.88, 95% CI [0.84, 0.93]). The funnel plot for mental health issues is shown in Figure 9. Here, the trim‐and‐fill analysis detected a slight asymmetry and imputed one effect size on the right side of the plot. However, results with this effect size included suggest a similarly sized and statistically significant effect (OR = 1.47, 95% CI [1.25, 1.73]).

**Figure 10:**
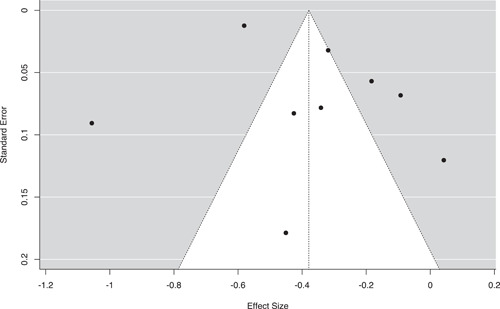
Funnel plot for attitudes toward police

Figure 10 displays the funnel plot for attitudes toward the police. While there is clearly significant variability in effect sizes across these studies, no significant asymmetry was detected by the trim‐and‐fill analysis. Egger's regression tests for crime and disorder outcomes (*t* = −0.56, *p* = 0.59), mental health outcomes (*t* = 0.74, *p* = 0.49), and attitudes toward the police (*t* = 1.61, *p* = 0.15) all failed to detect significant funnel plot asymmetry as well.

In sum, there is limited evidence of publication bias in our results. Any potential bias appears to be minor and not substantively meaningful for our overall results.

## 6 DISCUSSION

### 6.1 Summary of main results

The results of this systematic review and meta‐analysis point to both intended and unintended effects of pedestrian stop interventions. Analyzing 58 effect sizes across six discrete outcome groupings, we find that pedestrian stops lead to a reduction in crime at the geographic level but produce deleterious effects on the health, behavior, and attitudes of the individuals stopped by police. Our results can be summarized as follows: First, pedestrian stop interventions were associated with a statistically significant 13% decrease in crime for treatment areas relative to control. This effect was not accompanied by similar evidence of spatial displacement, and instead, we find a statistically significant diffusion of crime control benefits, with an average 7% decrease in crime for treatment displacement areas relative to control. Second, pedestrian stops were associated with a statistically significant 46% increase in the odds of a mental health issue for individuals stopped by police relative to those not stopped by police. These results extended to physical health issues as well, with treatment individuals demonstrating a 36% increase in the odds of a physical health issue relative to control individuals. Third, there was a significant effect of pedestrian stops on individual attitudes toward the police (*g* = −0.38). Specifically, individuals stopped by police were associated with significantly more negative attitudes than individuals not stopped by police (by a differential of approximately 18.6%). However, our narrative review of studies incorporating community surveys suggests that pedestrian stops do not negatively affect attitudes toward the police at the community‐level, indicating that these backfire effects may be limited to individuals directly subject to the intervention. Finally, pedestrian stops were associated with a statistically significant increase in self‐reported crime/delinquency (*g* = 0.30), with individuals experiencing direct police stops reporting a higher frequency of crime/delinquency, compared to those not experiencing direct police stops (by a differential of approximately 15%).

Additional analyses also point to several important effect size moderators. First, the negative effect of pedestrian stops on mental health outcomes are notably larger for studies analyzing youth samples. That is, studies measuring the mental health impacts of pedestrian stops on youth were associated with a 74% increase in the odds of a mental health issue while studies measuring similar impacts on adults were associated with only a 32% increase. We also find evidence to suggest that the crime reduction effects of pedestrian stops are significantly larger, and that the negative effects on attitudes toward the police are significantly smaller, for interventions occurring in the United States compared to Europe. US studies were associated with a 14% decrease in crime and a small but significant decrease in attitudes favorable to the police (*g* = −0.23), while European studies were associated with only a 5% decrease in crime and a moderate but significant decrease in attitudes favorable to the police (*g* = −0.65). However, it is important to note that these moderator analyses are subject to the same concerns regarding confounding variables as other forms of nonexperimental research (see Lipsey, 2003), and thus there may be unmeasured factors responsible for the significant differences in effect sizes observed between geographic locations. Finally, the size of the crime reduction effects for pedestrian stop interventions increased by a linear change of over 3% as the size of the targeted geographic areas decreased, but this effect was not statistically significant. This suggests that stop interventions targeted at micro‐geographic areas are likely to produce the strongest deterrent effects, however we lacked a sufficient number of studies to identify this as a statistically significant effect. On this note, it is important to point out that all of our moderator analyses were limited by small numbers of studies in respective groupings. Accordingly, there remains uncertainty as to the factors that moderate the impacts of pedestrian stop interventions, and we were unable to analyze several theoretically salient factors of interest (e.g., race/ethnicity, intrusiveness of police stops, etc.).

Taken together, our results suggest that pedestrian stops can be an effective crime control strategy, but one that comes with considerable drawbacks. Given the observed backfire effects in terms of individual health, attitudes, and behavior, it is not clear whether these interventions lead to any long‐term net gain or produce benefits that justify their non‐monetary costs. Our results also raise questions as to the mechanisms through which police stops may reduce crime. One common belief is that pedestrian stops produce a specific deterrent effect, or that individuals subject to a stop will alter their behavioral patterns to avoid future police interaction (see Rosenfeld & Fornango, 2014; Stafford & Warr, 1993). However, our finding of backfire effects on self‐reported crime/delinquency, coupled with area‐level decreases in crime, suggest that any deterrent effect associated with pedestrian stops may be more general in nature. Given that police stop interventions often involve increased police presence in high‐crime areas, these findings may also highlight the potential confounding effect of police stops with police presence toward the production of general deterrence. Despite this potential, we urge caution in the interpretation of our findings, particularly as they relate to person‐based studies. There is both a significant amount of heterogeneity in effect size estimates for many outcome measures, and considerable risk of bias toward control groups. Given the issues associated with establishing proper temporal ordering between pedestrian stops and person‐based outcomes and the difficulty involved with statistically controlling for an individual's likelihood of being stopped by the police, there remains a possibility of reverse causality. There was also an overall lack of random assignment in person‐based studies and only one experimental evaluation assessing place‐based crime outcomes, which greatly limits the potential to make strong causal inferences. Nonetheless, while there is a need for further research on the effects of pedestrian stops, the direction of effects across all outcome groupings is highly consistent.

### 6.2 Overall completeness and applicability of evidence

We conducted comprehensive search strategies intended to capture all studies published between 1970 and 2021 that met our eligibility criteria. Ultimately, only one eligible study that would have otherwise been included in our meta‐analysis was excluded due to insufficient data for an effect size calculation (Alderden et al., 2011). Thus, our results encompass a nearly complete representation of our population of eligible studies. Given the controversial nature of pedestrian stop interventions (White & Fradella, 2016) and the lack of existing meta‐analyses on the subject (see Koper & Mayo‐Wilson, 2006), our results are highly relevant and applicable to law enforcement agencies, public health agencies, advocacy groups, and policy organizations.

However, our search also identified areas in which the evidence base on pedestrian stops appears incomplete. First, only seven studies across six outcome groupings were conducted outside of the United States, and the vast majority of these studies were conducted in the United Kingdom. As such, there remains a lack of international research on the effects of pedestrian stops, and the findings of this review may have limited generalizability outside of the US and UK. Second, several of our analyzed outcomes contained a small number of studies. Specifically, only four unique samples measured both physical health issues and self‐reported crime/delinquency, calling for additional research on these outcomes. On a related note, 15 studies were associated with only four survey samples. While these surveys were often nationally representative and conducted using probability sampling methods, there is a possible dependency between outcomes taken from the same sample (i.e., individuals experiencing mental health issues may be more likely to experience physical health issues or negative attitudes toward the police, etc.). The completeness of this body of research may be increased through the incorporation of additional survey samples in future studies. There were also several outcomes specified in our initial protocol that we were unable to analyze due to a lack of eligible studies. Namely, there appears to be a lack of empirical knowledge concerning the impact of pedestrian stops on outcomes such as violence in police‐citizen encounters and officer misconduct. Finally, we did not include qualitative analyses in this review, and additional insight may be gained through synthesis of existing qualitative research.

### 6.3 Quality of the evidence

The overall quality of the evidence included in this review is low by conventional standards (see Weisburd et al., 2001) and the risk of bias toward control groups was deemed to be high for most outcome groupings. Only one eligible study used random allocation and the majority of remaining studies relied on multiple regression analyses to reduce the potential for selection bias. However, this approach is reliant on the ability to identify, observe, and measure all potentially confounding factors, and given this difficulty, the potential for omitted variable bias is an ever‐present concern (see Bushway & Apel, 2010; Weisburd et al., 2022). For place‐based studies of crime and disorder, roughly half of all included studies identified control areas based on considerations of comparability to treatment areas. Similarly, half of our included studies on self‐reported crime/delinquency employed propensity matching techniques to equate treatment and control individuals on their likelihood of being stopped by police. These groups of studies were also able to establish appropriate temporal ordering, either through the inclusion of pre‐ and post‐intervention measures or by separating measurements into discrete waves of data collection. Thus, for individual and place‐based studies of crime and delinquency, we considered the quality of evidence to be moderately high and risk of bias was not a significant concern.

However, a major quality concern for studies measuring health outcomes and attitudes toward the police is the lack of clear temporal ordering. Outcome variables in these studies (e.g., depression, poor health, police legitimacy) are generally measured during the same wave of data collection as personal experience with police stops. As such, it is often difficult to determine when health issues or negative attitudes toward the police developed and whether an individual's experience with pedestrian stops preceded the development of these conditions. Given that negative health conditions and attitudes toward the police may increase the likelihood that individuals come into contact with police in general (Thompson & Kahn, 2016), there is clear risk of bias toward control groups for these outcome measures. While stronger research designs controlling for baseline levels of mental or physical health and/or the inclusion of propensity score weighting (see Dennison & Finkeldey, 2021; Geller, 2017) report results that are highly consistent with those of our overall findings, there is considerable potential for the quality of existing evidence to impact the findings of this review.

### 6.4 Limitations and potential biases in the review process

We conducted a number of rigorous search strategies to capture a broad range of published and unpublished research. While there were no specific limitations in our review process, we encountered some issues that limited our ability to calculate effect sizes and assess certain outcomes that were specified in our initial protocol. First, we were unable to calculate an effect size for one eligible place‐based study measuring crime and disorder. Additionally, we were unable to meta‐analyze outcomes related to community surveys, given a lack of clear conceptual overlap in these outcomes and in the forms of data reported. Second, we did not identify eligible studies providing dedicated assessments of violence in police citizen encounters or officer misbehavior, and thus we are unable to speak to the effect of pedestrian stop interventions on these outcomes. Finally, we did not explicitly incorporate our risk of bias ratings into our meta‐analysis. However, these ratings largely overlapped with the methodological characteristics that we used during our moderator analyses.

### 6.5 Agreements and disagreements with other studies or reviews

A prior Campbell systematic review on efforts to reduce illegal possession and carrying of firearms found that directed patrol interventions focused on suppression of illegal gun carrying were effective at reducing gun crime (Koper & Mayo‐Wilson, 2012). Our findings provide similar conclusions, as many of the place‐based interventions included in this review employed pedestrian stops as a major component of targeted patrol efforts (see McGarrell et al., 2002; Ratcliffe et al., 2011; Sherman & Rogan, 1995; Weisburd et al., 2016). In fact, macro‐level interventions were the only place‐based studies that failed to demonstrate a statistically significant deterrent effect, though this finding is limited by the exceedingly small number of studies measuring the macro‐level effects of pedestrian stops.

The deterrent effect of pedestrian stops within targeted patrol efforts is also consistent with extant reviews of “hot spot” policing interventions (Braga et al., 2019, p. 1), though this finding brings into question the mechanism of effect in these interventions. That is, pedestrian stops may lead to a reduction in crime because they involve a targeted increase in police visibility within high crime areas rather than any deterrent effect produced by the stops themselves (see Weisburd et al., 2014). Unfortunately, we are unable to distinguish between these causal mechanisms in this review. A similar limitation was noted by the National Academy of Sciences (NAS) panel on proactive policing in their consensus review of the evidence on proactive policing interventions (see Weisburd & Majmundar, 2018; Weisburd et al., 2019). The NAS panel found strong evidence to suggest that high‐volume pedestrian stops produce a deterrent effect when targeted at places with violence or gun crime problems, but that these interventions were often confounded with hot spot policing practices. This finding was accompanied by more modest evidence of jurisdictional impacts, which were often of lower methodological quality. Design limitations prevented the NAS panel from making causal inferences regarding the community‐level impacts of pedestrian stop interventions, but they noted clear evidence of negative effects stemming from personal experiences with police stops. Our findings are highly consistent with those of the NAS panel, suggesting significant deterrent effects of pedestrian stops at micro and meso‐level geographic areas, accompanied by significant negative effects on personal attitudes, health, and behavior. However, our review extends these findings by providing a systematic search of studies and applying meta‐analytic techniques.

## 7 AUTHORS' CONCLUSIONS

### 7.1 Implications for practice and policy

The findings from this systematic review and meta‐analysis paint a complicated picture for practitioners and policymakers. On one hand, our results tend to support the long‐held belief among law enforcement agencies that pedestrian stops constitute an important crime prevention tool (see Baker & Goldstein, 2012). Particularly when targeted at specific high‐crime areas, pedestrian stop interventions are associated with significant and meaningful reductions in crime. In contrast, however, our results also support perspectives that are critical of pedestrian stops as a crime prevention tactic (see Fagan & Davies, 2000; Gelman et al., 2007). We find strong and significant evidence to suggest that being stopped by police is associated with worsening mental and physical health, attitudes toward the police, and even elevated levels of personal offending and delinquent behavior. Furthermore, we find preliminary evidence to suggest that the deleterious effects of pedestrian stops on mental health outcomes are particularly pronounced for youth, who are simultaneously more vulnerable to these encounters and at an increased risk of experiencing them (Geller, 2017). While the current review did not include measures of racial disparity, it is also well‐established that minority populations are more likely to experience these forms of police contact (Braga et al., 2019; Fagan & Davies, 2000; MacDonald & Braga, 2019; Ridgeway, 2007). Thus, the negative individual‐level impacts of pedestrian stops may be disproportionately concentrated within minority and/or disadvantaged populations, perhaps furthering pre‐existing socioeconomic disadvantage and deepening the divide between police and community members. Given these concerns, the central question for police agencies and policymakers is whether the positive effects produced by pedestrian stop interventions outweigh the negative effects, and whether agencies *should* use pedestrian stops, regardless of whether the intervention *is* effective.

In this regard, it is important to consider the findings of this review alongside those examining other proactive policing interventions. Recent reviews on hot spots policing and problem‐oriented policing (POP) have reported crime reduction effects that are larger in magnitude than those reported here, without similar backfire effects on individual and community outcomes (see Braga et al., 2019; Hinkle et al., 2020). For example, Braga and Weisburd (2020) found that hot spots policing interventions were associated with a 16% reduction in crime, and Hinkle et al. (2020) found that POP interventions were associated with a 33.8% reduction in crime, for treatment areas relative to control areas. These tactics are also characterized by a larger body of research with considerably stronger methodological rigor than those included in this review. Thus, law enforcement agencies seeking to employ proactive policing tactics to reduce crime and disorder should consider interventions involving increased police visibility alongside community engagement and problem‐solving efforts (see Braga et al., 2019). These tactics holds promise in maximizing crime prevention while simultaneously increasing communication and cooperation with community members.

From a policy perspective, there is also still uncertainty as to the mechanism through which pedestrian stops reduce crime and disorder. As the NAS panel on proactive policing noted, pedestrian stops are often confounded with the presence of directed patrol at high‐crime areas, and it is possible that hot spots policing is responsible for some if not most of the observed crime reductions. While several existing studies find evidence to suggest a deterrent effect of stops themselves (MacDonald et al., 2016; McGarrell et al., 2000; Sherman & Rogan, 1995), others find evidence to suggest that the primary deterrent mechanism may be increased police presence (Braakman, 2022). For example, both Sherman and Rogan (1995) and McGarrell et al. (2000) observed significant reductions in violent and gun‐related crime following an increase in police stops but did not observe similar reductions in other types of crime that would still be subject to a general deterrent effect of police presence. These results led Sherman and Rogan to “refute the hypothesis of general deterrence due to more visible patrol presence” (p. 688). MacDonald et al. (2016) found that the crime reduction effect of pedestrian stops in New York City was limited to probable cause stops, rather than stops conducted based on more general suspicion. This suggests that stops may have a unique crime reduction effect, but that the overuse of stops is unlikely to lead to a greater reduction in crime. More recently, Braakman (2022) concluded that the deterrent effect of pedestrian stops was likely due to an increase in police presence, finding a significant reduction in anti‐social behavior associated with pedestrian stops but no similar impact on violent crime. Thus, more research is needed on these mechanisms as it is unclear whether pedestrian stops produce a deterrent effect independent of police presence alone.

Law enforcement agencies should also consider the nature of the contact between police officers and citizens during pedestrian stops. While too few studies in our review provided comparisons between control conditions and police stops of varying intrusiveness/satisfaction levels, there is evidence to suggest that the quality of police contact may be as important as the contact itself (see Harris & Jones, 2020; Mazerolle et al., 2013; Tyler et al., 2014). Indeed, several of our eligible studies find that the intrusiveness associated with a police stop (Harris & Jones, 2020), satisfaction with police contact (Baćak & Apel, 2021; Slocum et al., 2016), and perceptions of respect and procedural justice (Friedman et al., 2004; Slocum et al., 2016) may mediate the effect of these stops on individual‐level outcomes. If so, it is possible that police agencies can mitigate the negative effects of pedestrian stop interventions through a focus on procedural justice during police‐citizen encounters, though we are not presently able to make such a conclusion. Support for this possibility comes from a recent three city randomized trial which provided intensive procedural justice training to officers assigned to a procedural justice hot spots condition (as contrasted with non‐trained officers in the standard hot spots condition). That study found positive impacts on resident views of police violence and harassment (Weisburd et al., 2022).

In sum, there are still important and understudied aspects of pedestrian stop interventions. However, current evidence indicates that the use of high‐volume pedestrian stops leads to both meaningful reductions in crime and a broad range of negative effects for the individuals subject to these stops.

### 7.2 Implications for research

There is a clear need for additional research on pedestrian stop interventions, particularly using experimental or strong quasi‐experimental methods. Future studies separating personal experience with pedestrian stops, attitudes toward the police, and mental/physical health issues into separate waves of data collection (and/or or employing pre‐ and post‐intervention outcome measurements) would go a long way toward establishing temporal ordering and strengthening any causal inferences related to personal attitudes and health outcomes. Additional use of propensity score matching techniques, specifically for studies examining attitudes toward the police, is also needed to limit the potential for selection bias. This is exceedingly true considering the lack of random allocation used in these studies and the feasibility issues that are likely involved in the experimental analysis of pedestrian stops at the individual‐level. Furthermore, there is an apparent lack of high‐quality research examining the effect of pedestrian stop interventions on violence and misbehavior in police‐citizen interactions. If high‐volume pedestrian stops lead to additional use‐of‐force incidents or citizen complaints, then the negative impacts of these interventions may be even broader than those presented in this review. In this regard, future efforts may benefit from including a synthesis of qualitative research that explores individuals' experiences and perceptions of police stops. Along with this, existing research has largely been limited to contexts within the United States and the United Kingdom. Given evidence that similar strategies are being used in other parts of the world (Miller et al., 2008), future research is needed in these settings. Additional research with youth samples is also needed, as our ability to assess the unique effects of police stops on this demographic was limited. Finally, additional studies separating the effect of pedestrian stops by racial/ethnic groupings and levels of satisfaction/procedural justice associated with the police stop itself are needed. Although there were too few studies of this nature in the current review to provide dedicated analyses, extant research and theory clearly indicate that race/ethnicity and the nature of police contact may be important moderating factors.

### ROLES AND RESPONSIBILITIES

• Content: Petersen, Weisburd, Fay
• Systematic review methods: Petersen, Weisburd, Fay, Eggins, Mazerolle
• Statistical analysis: Petersen, Weisburd
• Information retrieval: Petersen, Weisburd, Fay, Eggins, Mazerolle

## DECLARATIONS OF INTEREST

Petersen and Fay have not conducted evaluation research or published on the effectiveness of pedestrian stops. Neither author would be uncomfortable with any results produced by the review.

Weisburd has conducted a number of evaluations of hot spots policing and has written an article on SQFs showing some degree of effectiveness. He was also the Chair of the National Academy of Sciences panel on proactive policing, which suggested effectiveness of targeted pedestrian stops (though little impact for broadly focused policies). Weisburd would be comfortable with outcomes that run counter to these prior findings. He is a member of the Campbell Collaboration Crime and Justice Coordinating Group. To manage potential conflicts of interest, Weisburd will not be involved in the editorial or formal approval process for this protocol or the subsequent review.

Eggins has not been involved in any evaluations of police‐initiated pedestrian police‐stops. Eggins is an Editor for the Campbell Crime and Justice Coordinating Group (former Managing and Associate Editor) and so will not be involved in the editorial or formal approval process for this protocol or the subsequent review.

Mazerolle has conducted a number of evaluations of policing interventions, some of which have contained police stops as one component of the intervention. She is also a former Co‐chair of the Campbell Collaboration Crime and Justice Coordinating Group. To manage these potential conflicts of interest, Mazerolle will not be involved in the editorial or formal approval process for this protocol or the subsequent review, nor will she independently decide on study eligibility, code studies, or conduct statistical or risk of bias analyses.

## Supporting information

Supporting information.

## Footnotes

1 Here, we considered youth to consist of individuals under the age of 18.

2 Some of our email attempts were returned as undeliverable, and thus not all authors were successfully contacted.

3 Item A described in this section is adapted from item 1.1 of the Cochrane RoB2 instrument. Item B is adapted from item 1.3 of the Cochrane RoB2 instrument. Item C is adapted from item 1.4 of the Cochrane ROBINS‐I instrument. Item D is adapted from item 2.4 of the Cochrane RoB2 instrument. Item E is adapted from item 3.4 of the Cochrane RoB2 instrument. Item F was created by the authors of this review after determining its importance within this body of research.

4 See http://markummitchell.github.io/engauge-digitizer/

5 This calculation involves dividing the 95% confidence interval by the z‐value associated with 95% of a two‐tailed standard normal distribution as follows: $SE=\frac{{\mathrm{CI}}_{\mathrm{Upper}}-{\mathrm{CI}}_{\mathrm{Lower}}}{3.92}.$

6 Unfortunately, measures of the satisfaction or procedural justice associated with a police stop were not reported frequently or consistently enough within any given outcome grouping to permit a separate moderator or sub‐group analysis.

7 Note, however, that one study (Boydstun, 1975) reported the number of citizen complaints both before and after a pedestrian stops intervention, but no complaints were filed in either time period.

8 Studies that could not be located are considered awaiting classification and are listed in the reference section. Note, however, that many of these results were broken or incomplete references (particularly those in non‐English languages). Thus, we only list results for which we could locate a complete reference.

9 One eligible study lacked the data necessary to calculate an effect size (Alderden et al., 2011).

10 As an example, chapter 7 in Bradford (2017) contains an analysis comparing trust in the police among individuals with varying degrees of stop experience. However, most respondents in this sample experienced car stops rather than foot stops (nearly 80%). Thus, we excluded this analysis and others similar to it.

11 This conversion can be expressed as the following (see Lipsey & Wilson, 2001): $\frac{g}{\sqrt{g^{2}+4}}$.

12 Here we mean that we were not able to derive a more general effect estimate from these studies, and that the effect sizes used in our analysis represent the effect of being both stopped and stopped in an unfair, false, or dissatisfied manner.

13 We also include our sole block randomized experiment (Ratcliffe et al., 2011) as a matched design in this analysis.

## References

[cl21302-bib-0001] * Alderden, M. A. , Schuck, A. M. , Stephens, C. D. , Lavery, T. A. , Johnston, R. M. , & Rosenbaum, D. P. (2011). Gang hot spots policing in Chicago: An evaluation of the deployment operations center process. University of Illinois‐Chicago.

[cl21302-bib-0002] Baćak, V. , & Apel, R. (2020). The thin blue line of health: Police contact and wellbeing in Europe. Social Science & Medicine, 267, 112404. 10.1016/j.socscimed.2019.112404 31345610

[cl21302-bib-0003] Baćak, V. , & Apel, R. (2021). Police fairness and legitimacy across the post‐communist divide in Europe. Law & Society Review, 55(3), 473–495.

[cl21302-bib-0004] Baćak, V. , & Nowotny, K. M. (2020). Race and the association between police stops and depression among young adults: A research note. Race and Justice, 10(3), 363–375.

[cl21302-bib-0005] Boydstun, J. E. (1975). San Diego field interrogation: Final report. Police Foundation.

[cl21302-bib-0006] Cohen, J. , & Ludwig, J. (2003). Policing crime guns. In J. Ludwig , & P. J. Cook (Eds.), Evaluating gun policy: Effects on crime and violence (pp. 217–239). Brookings Institution Press.

[cl21302-bib-0007] Dennison, C. R. , & Finkeldey, J. G. (2021). Self‐reported experiences and consequences of unfair treatment by police. Criminology, 59(2), 254–290.

[cl21302-bib-0008] Friedman, W. , Lurigio, A. J. , Greenleaf, R. , & Albertson, S. (2004). Encounters between police officers and youths: The social costs of disrespect. Journal of Crime and Justice, 27(2), 1–25.

[cl21302-bib-0009] Geller, A. (2017). *Policing America's children: Police contact and consequences among teens in fragile families* (CRCW Working Paper WP18‐02‐FF). Center for Research on Child Wellbeing.

[cl21302-bib-0010] Geller, A. , Fagan, J. , Tyler, T. , & Link, B. G. (2014). Aggressive policing and the mental health of young urban men. American Journal of Public Health, 104(12), 2321–2327. 10.2105/ajph.2014.302046 25322310 PMC4232139

[cl21302-bib-0011] Harris, J. W. , & Jones, M. S. (2020). Shaping youths' perceptions and attitudes toward the police: Differences in direct and vicarious encounters with police. Journal of criminal justice, 67, 101674.

[cl21302-bib-0012] Hirschtick, J. (2017). *Associations between police encounters and mental health status in Chicago* [Doctoral Dissertation, University of Illinois at Chicago].

[cl21302-bib-0013] Hirschtick, J. L. , Homan, S. M. , Rauscher, G. , Rubin, L. H. , Johnson, T. P. , Peterson, C. E. , & Persky, V. W. (2020). Persistent and aggressive interactions with the police: Potential mental health implications. Epidemiology and Psychiatric Sciences, 29, e19. 10.1017/S2045796019000015 PMC8061162 30714560

[cl21302-bib-0014] * Hofer, M. S. , Womack, S. R. , & Wilson, M. N. (2020). An examination of the influence of procedurally just strategies on legal cynicism among urban youth experiencing police contact. Journal of Community Psychology, 48(1), 104–123. 31523832 10.1002/jcop.22242

[cl21302-bib-0015] Jackson, D. B. , Testa, A. , Fix, R. L. , & Mendelson, T. (2021). Adolescent police stops, self‐harm, and attempted suicide: Findings from the UK millennium cohort study, 2012‒2019. American Journal of Public Health, 111(10), 1885–1893. 34554817 10.2105/AJPH.2021.306434 PMC8561199

[cl21302-bib-0016] * Jackson, D. B. , Testa, A. , & Vaughn, M. G. (2020). Low self‐control and legal cynicism among at‐risk youth: An investigation into direct and vicarious police contact. Journal of Research in Crime and Delinquency, 57(6), 741–783.

[cl21302-bib-0017] Jackson, D. B. , Testa, A. , Vaughn, M. G. , & Semenza, D. C. (2020). Police stops and sleep behaviors among at‐risk youth. Sleep Health, 6(4), 435–441. 32305306 10.1016/j.sleh.2020.02.006

[cl21302-bib-0018] * Kochel, T. R. , & Nouri, S. (2021). Drivers of perceived safety: Do they differ in contexts where violence and police saturation feel ‘normal’? Journal of Crime and Justice, 44(5), 515–534.

[cl21302-bib-0019] Lee, J. S. , Tajima, E. A. , Herrenkohl, T. I. , & Hong, S. (2017). Effects of formal and informal deviant labels in adolescence on crime in adulthood. Social Work Research, 41(2), 97–110.

[cl21302-bib-0020] * Lerman, A. E. , & Weaver, V. (2014). Staying out of sight? Concentrated policing and local political action. The Annals of the American Academy of Political and Social Science, 651(1), 202–219.

[cl21302-bib-0021] Lewis, M. W. , & Wu, L. (2021). Exposure to community violence versus overpolicing and PTSD among African American university students. Journal Of Human Behavior in the Social Environment, 31(8), 1026–1039.

[cl21302-bib-0022] MacDonald, J. , Fagan, J. , & Geller, A. (2016). The effects of local police surges on crime and arrests in New York city. PLoS One, 11, e0157223. 27310252 10.1371/journal.pone.0157223 PMC4911104

[cl21302-bib-0023] McCandless, R. , Feist, A. , Allan, J. , & Morgan, N. (2016). Do initiatives involving substantial increases in stop and search reduce crime? Assessing the impact of operation BLUNT 2. Home Office.

[cl21302-bib-0024] McFarland, M. J. , Geller, A. , & McFarland, C. (2019). Police contact and health among urban adolescents: The role of perceived injustice. Social Science & Medicine, 238, 112487. 31445303 10.1016/j.socscimed.2019.112487

[cl21302-bib-0025] McGarrell, E. F. , Chermak, S. M. and Weiss, A. (2000). *Reducing firearms violence through directed police patrol*: *Final report on the evaluation of the Indianapolis Police Department's directed patrol project*. National Institute of Justice, Final Report to the United States Department of Justice, grant No. 95‐IJ‐CX‐0019.

[cl21302-bib-0026] McGarrell, E. F. , Chermak, S. , & Weiss, A. (2002). *Reducing gun violence: Evaluation of the Indianapolis Police Department's directed patrol project*. US Department of Justice, Office of Justice Programs, National Institute of Justice.

[cl21302-bib-0027] Murray, K. (2014). *The proactive turn*: *Stop and search in Scotland* [PhD Thesis, University of Edinburgh].

[cl21302-bib-0028] Murray, K. , McVie, S. , Farren, D. , Herlitz, L. , Hough, M. , & Norris, P. (2021). Procedural justice, compliance with the law and police stop‐and‐search: A study of young people in England and Scotland. Policing and Society, 31, 263–282. 10.1080/10439463.2020.1711756

[cl21302-bib-0179] Paternoster, R. , & Iovanni, L. (1989). The Labeling perspective and delinquency: An elaboration of the theory and an assessment of the evidence. Justice Quarterly, 6(3), 359–394. 10.1080/07418828900090261

[cl21302-bib-0029] Ratcliffe, J. H. , Taniguchi, T. , Groff, E. R. , & Wood, J. D. (2011). The Philadelphia foot patrol experiment: A randomized controlled trial of police patrol effectiveness in violent crime hotspots. Criminology, 49(3), 795–831. 10.1111/j.1745-9125.2011.00240.x

[cl21302-bib-0030] Rosenbaum, D. P. , Schuck, A. M. , Costello, S. K. , Hawkins, D. F. , & Ring, M. K. (2005). Attitudes toward the police: The effects of direct and vicarious experience. Police Quarterly, 8(3), 343–365.

[cl21302-bib-0180] Sánchez‐Meca, J. , Marín‐Martínez, F. , & Chacón‐Moscoso, S. (2003). Effect‐size indices for dichotomized outcomes in meta‐analysis. Psychological Methods, 8(4), 448. 14664682 10.1037/1082-989X.8.4.448

[cl21302-bib-0031] Sherman, L. W. , & Rogan, D. P. (1995). Effects of gun seizures on gun violence: hot spots patrol in Kansas City. Justice Quarterly, 12(4), 673–693.

[cl21302-bib-0032] Sherman, L. W. , Shaw, J. , & Rogan, D. (1995). The Kansas City gun experiment. In Research in brief. National Institute of Justice: Research in brief.

[cl21302-bib-0033] Singer, L. (2013). London riots: Searching for a stop. Policing, 7(1), 32–41.

[cl21302-bib-0034] Slocum, L. A. , Ann Wiley, S. , & Esbensen, F.‐A. (2016). The importance of being satisfied: A longitudinal exploration of police contact, procedural injustice, and subsequent delinquency. Criminal Justice and Behavior, 43(1), 7–26.

[cl21302-bib-0035] Sundaresh, R. , Yi, Y. , Roy, B. , Riley, C. , Wildeman, C. , & Wang, E. A. (2020). Exposure to the US criminal legal system and well‐being: A 2018 cross‐sectional study. American Journal of Public Health, 110(S1), S116–S122. 31967880 10.2105/AJPH.2019.305414 PMC6987921

[cl21302-bib-0036] Swaner, R. , & Brisman, A. (2014). Legal cynicism among civically‐engaged youth. Varstvoslovje: Journal of Criminal Justice & Security, 16(4), 492–517.

[cl21302-bib-0037] Testa, A. , Jackson, D. B. , & Semenza, D. (2021). Unfair police treatment and sleep problems among a national sample of adults. Journal of Sleep Research, 30(6), e13353. 33870581 10.1111/jsr.13353

[cl21302-bib-0038] Turney, K. (2021). Depressive symptoms among adolescents exposed to personal and vicarious police contact. Society and Mental Health, 11(2), 113–133.

[cl21302-bib-0039] Tyler, T. R. , Fagan, J. , & Geller, A. (2014). Street stops and police legitimacy: Teachable moments in young urban men's legal socialization: street stops and police legitimacy. Journal of Empirical Legal Studies, 11(4), 751–785.

[cl21302-bib-0181] Weisburd, D. , & Eck, J. E. (2004). What can police do to reduce crime, disorder, and fear. The ANNALS of the American Academy of Political and Social Science, 593(1), 42–65. 10.1177/0002716203262548

[cl21302-bib-0040] Weisburd, D. , Wooditch, A. , Weisburd, S. , & Yang, S.‐M. (2016). Do stop, question, and frisk practices deter crime? Criminology & Public Policy, 15(1), 31–56. 10.1111/1745-9133.12172

[cl21302-bib-0041] Wheelock, D. , Stroshine, M. S. , & O'Hear, M. (2019). Disentangling the relationship between race and attitudes toward the police: Police contact, perceptions of safety, and procedural justice. Crime & Delinquency, 65(7), 941–968.

[cl21302-bib-0042] Wiley, S. A. , & Esbensen, F.‐A. (2016). The effect of police contact: Does official intervention result in deviance amplification? Crime & Delinquency, 62(3), 283–307.

[cl21302-bib-0043] Wiley, S. A. , Slocum, L. A. , & Esbensen, F. A. (2013). The unintended consequences of being stopped or arrested: An exploration of the labeling mechanisms through which police contact leads to subsequent delinquency. Criminology, 51(4), 927–966.

[cl21302-bib-0182] Braakmann, N. (2022). Does stop and search reduce crime? Evidence from street‐level data and a surge in operations following a high‐profile crime. Journal of the Royal Statistical Society, Series A, 185(3), 1370–1397.

[cl21302-bib-0044] Bradford, B. (2017). Stop and search and police legitimacy. Routledge.

[cl21302-bib-0045] Bryant, K. M. , Collins, G. M. , & White, M. D. (2015). Shawnee, Kansas, Smart Policing Initiative: Reducing Crime and Automobile Collisions Through Data‐driven Approaches to Crime and Traffic Safety (DDACTS). CNA.

[cl21302-bib-0183] Del Toro, J. , Lloyd, T. , Buchanan, K. S. , Robins, S. J. , Bencharit, L. Z. , Smiedt, M. G. , Reddy, K. S. , Pouget, E. R. , Kerrison, E. M. , & Goff, P. A. (2019). The criminogenic and psychological effects of police stops on adolescent black and Latino boys. *Proceedings of the National Academy of Sciences*, 116(17), 8261–8268. 10.1073/pnas.1808976116 PMC6486703 30962370

[cl21302-bib-0046] DeVylder, J. E. , Frey, J. J. , Cogburn, C. D. , Wilcox, H. C. , Sharpe, T. L. , Oh, H. Y. , Nam, B. , & Link, B. G. (2017). Elevated prevalence of suicide attempts among victims of police violence in the USA. Journal of Urban Health, 94(5), 629–636. 28534243 10.1007/s11524-017-0160-3 PMC5610123

[cl21302-bib-0047] DeVylder, J. E. , Oh, H. Y. , Nam, B. , Sharpe, T. L. , Lehmann, M. , & Link, B. G. (2017). Prevalence, demographic variation and psychological correlates of exposure to police victimisation in four US cities. Epidemiology and Psychiatric Sciences, 26(5), 466–477. 27834166 10.1017/S2045796016000810 PMC6998899

[cl21302-bib-0048] Foster, K. , Jones, M. S. , & Pierce, H. (2022). Race and ethnicity differences in police contact and perceptions of and attitudes toward the police among youth. Criminal Justice and Behavior, 49(5), 660–680.

[cl21302-bib-0049] Hoover, L. , Wells, W. , Zhang, Y. , Ren, L. , & Zhao, J. (2016). Houston enhanced action patrol: examining the effects of differential deployment lengths with a switched replication design. Justice Quarterly, 33(3), 538–563.

[cl21302-bib-0050] Jackson, D. B. , Del Toro, J. , Semenza, D. C. , Testa, A. , & Vaughn, M. G. (2021). Unpacking racial/ethnic disparities in emotional distress among adolescents during witnessed police stops. Journal of Adolescent Health, 69(2), 248–254. 10.1016/j.jadohealth.2021.02.021 33814280

[cl21302-bib-0051] Jackson, D. B. , & Testa, A. (2022). Police stops and adolescent sleep problems: findings from the UK millennium cohort study. Journal of Sleep Research, 31, e13585. 35289002 10.1111/jsr.13585 PMC9786844

[cl21302-bib-0052] Kennedy, L. , Caplan, J. , & Piza, E. (2015). A multi‐jurisdictional test of risk terrain modeling and a place‐based evaluation of environmental risk‐based patrol deployment strategies. Rutgers University.

[cl21302-bib-0053] Lehrer, S. F. , & Lepage, L. P. (2020). How do NYPD officers respond to terror threats? Economica, 87(347), 638–661.

[cl21302-bib-0054] McFarland, M. J. , Taylor, J. , & McFarland, C. A. S. (2018). Weighed down by discriminatory policing: perceived unfair treatment and black‐white disparities in waist circumference. SSM‐Population Health, 5, 210–217. 30094316 10.1016/j.ssmph.2018.07.002 PMC6072653

[cl21302-bib-0055] Miller, J. , Bland, N. , Quinton, P. , & Willis, C. F. (2000). The impact of stops and searches on crime and the community (No. 127). Home Office, Policing and Reducing Crime Unit, Research, Development and Statistics Directorate.

[cl21302-bib-0056] Ostrom, E. , & Whitaker, G. (1973). Does local community control of police make a difference? Some preliminary findings. American Journal of Political Science, 17, 48–76.

[cl21302-bib-0057] Rosenfeld, R. , Deckard, M. J. , & Blackburn, E. (2014). The effects of directed patrol and self‐initiated enforcement on firearm violence: A randomized controlled study of hot spot policing. Criminology, 52(3), 428–449.

[cl21302-bib-0184] Rosenfeld, R. , & Fornango, R. (2014). The impact of police stops on precinct robbery and burglary rates in New York City, 2003–2010. Justice Quarterly, 31(1), 96–122. 10.1080/07418825.2012.712152

[cl21302-bib-0185] Rosenfeld, R. , & Fornango, R. (2017). The relationship between crime and stop, question, and frisk rates in New York City neighborhoods. Justice Quarterly, 34(6), 931–951. 10.1080/07418825.2016.1275748

[cl21302-bib-0058] Sargeant, E. , Davoren, N. , & Murphy, K. (2021). The defiant and the compliant: how does procedural justice theory explain ethnic minority group postures toward police? Policing and Society, 31(3), 283–303.

[cl21302-bib-0059] Sewell, A. A. , & Jefferson, K. A. (2016). Collateral damage: the health effects of invasive police encounters in New York city. Journal of Urban Health, 93(1), 42–67. 26780583 10.1007/s11524-015-0016-7 PMC4824697

[cl21302-bib-0060] Shaw, J. W. (1995). Community policing against guns: Public opinion of the Kansas city gun experiment. Justice Quarterly, 12(4), 695–710.

[cl21302-bib-0061] Testa, A. , Turney, K. , Jackson, D. B. , & Jaynes, C. M. (2022). Police contact and future orientation from adolescence to young adulthood: Findings from the pathways to desistance study. Criminology, 60(2), 263–290.

[cl21302-bib-0186] Tiratelli, M. , Quinton, P. , & Bradford, B. (2018). Does stop and search deter crime? Evidence from ten years of London‐wide data. The British Journal of Criminology, 58(5), 1212–1231. 10.1093/bjc/azx085

[cl21302-bib-0062] Turney, K. , Testa, A. , & Jackson, D. B. (2022). Police stops and the erosion of positive future orientation among urban adolescents. Journal of Adolescent Health, 71, 180–186. 10.1016/j.jadohealth.2022.02.015 35537889

[cl21302-bib-0063] Villaveces, A. (2000). Effect of a ban on carrying firearms on homicide rates in 2 Colombian cities. Journal of the American Medical Association, 283(9), 1205–1209. 10703790 10.1001/jama.283.9.1205

[cl21302-bib-0064] Anderson, J. W. (2000). Innovative program reels in serious traffic offenders. Police Chief, 67(7), 48–52.

[cl21302-bib-0065] Bannon, J. D. , & Nichols, J. F. (1972). S.T.R.E.S.S.: Zero visibility policing [operations, techniques and results achieved by a police patrol unit in Detroit, Mich., using the acronym STRESS‐‐Stop the robberies, enjoy safe streets]. Police Chief, 39(6), 32–34.

[cl21302-bib-0066] Brown, D. (1997). *PACE ten years on: A review of research*: *A research and statistics directorate report*. Home Office Research, Development and Statistics Directorate.

[cl21302-bib-0067] Brown, S. (2020). *There's a first time for everything*: *First police contact and its effect on offending* [Doctoral Dissertation, Florida State University].

[cl21302-bib-0068] Budz, D. (2001, September). *Combating residential burglary*: *A case study*. Paper presented at the Sixth Annual International CPTED Conference, Brisbane, Australia.

[cl21302-bib-0069] Craig, M. O. (2018). *Stop, question, and (cognitive) dissonance*: *Social control agents and justifications for racial and ethnic disproportionality in vehicle stops* [Doctoral dissertation].

[cl21302-bib-0070] Cummings, K. M. , & Coogan, K. (1992). Organizing communities to prevent the sale of tobacco products to minors. International Quarterly of Community Health Education, 13(1), 77–86. 10.2190/Y9G7-LD6A-F3HE-LM31 20841232

[cl21302-bib-0071] Duru, H. , & Akbas, H. (2021). Measuring hot spots policing in non‐research settings. International Journal of Law, Crime and Justice, 65, 100468.

[cl21302-bib-0072] Бондарь, А. Г. (2020). Актуальные проблемы в деятельности специализированных подразделений органов внутренних дел по вопросам противодействия коррупционной преступности. Проблемы экономики и юридической практики, 16(2), 303–307.

[cl21302-bib-0073] Garrett, R. L. (2001). Changing behavior begins with data: Collecting traffic stop data is a means to identify profiling and stop it in its tracks. Law Enforcement Technology, 28(4), 100–108.

[cl21302-bib-0074] Greenleaf, R. , Flexon, J. , & Lurigio, A. (2007). *November student attitudes toward the police: Focusing on the importance of social bonds and officer treatment*. Paper presented at the annual meeting of the American Society of Criminology.

[cl21302-bib-0075] Gumbhir, V. K. (2004). *Final report on the Eugene police department's vehicle stop data (2002*–*2003), condensed report*. University of Oregon, Submitted to the Eugene Police Department, Eugene, OR.

[cl21302-bib-0076] Gumbhir, V. (2008, November). *But is it racial profiling? Identifying evidence of pretext stops in vehicle stop data*. Paper presented at the annual meeting of the ASC Annual Meeting, St. Louis Adam's Mark, St. Louis, MO.

[cl21302-bib-0077] Hallsworth, S. , McGuire, M. , & Hingwan, K. (2006). *Examining proportionality and disproportionality in the exercise of stop and search in the City of London Police: A report for the City of London Police*. London Metropolitan University.

[cl21302-bib-0078] Hanink, P. A. (2018). *Toward a phenomenology of racialized police violence* [Doctoral Dissertation, UC Irvine].

[cl21302-bib-0079] Irlbeck, D. , & Ryan, V. (2008). *Duration of vehicle stop, duration of vehicle search: Do they vary by race/ethnicity of driver?* Paper presented at the annual meeting of the ASC Annual Meeting, St. Louis Adam's Mark.

[cl21302-bib-0080] Jahn, J. (2020). *Mass incarceration and aggressive policing: Contextual and direct determinants of population health* [Doctoral dissertation, Harvard University].

[cl21302-bib-0081] Johnson, B. D. (1972). *Police enforcement of drug laws*. Paper presented at the American Sociological Association, New York.

[cl21302-bib-0082] Lamberth, J. C. (2005). *Racial profiling data analysis study: Final report for the San Antonio Police Department*. Lamberth Consulting.

[cl21302-bib-0083] Lurigio, A. J. , Flexon, J. L. , & Greenleaf, R. G. (2008). Antecedents to gang membership: Attachments, beliefs, and street encounters with the police. Journal of Gang Research, 15(4), 15–34.

[cl21302-bib-0084] Massey, S. G. , & Kauffman, R. A. (2014). *Who gets to feel safe? Crime and law enforcement in a small city in upstate New York*. Paper presented at the 122nd American Psychological Association Annual Convention, Washington, DC.

[cl21302-bib-0085] Mors, T. (2008). *Best practices for police relative to recognizing extremists*. Papers presented at the American Society of Criminology annual meeting.

[cl21302-bib-0086] van Ooyen, D. (1982). New countermeasures against drinking drivers. In *Proceedings International Council on Alcohol, Drugs and Traffic Safety Conference* (Vol. 1982, pp. 142–151). International Council on Alcohol, Drugs and Traffic Safety.

[cl21302-bib-0087] Petrunik, M. , & Manyoni, J. R. (1991). *Race, socioeconomic conditions, and crime in Canadian cities: An exploratory study*. Paper presented at the Society for the Study of Social Problems.

[cl21302-bib-0088] Reitzel, J. (2007, November). *Law enforcement strategies and the line between racially and non‐racially targeted policing*. Paper presented at the American Society of Criminology Annual Meeting, Atlanta, GA.

[cl21302-bib-0089] Strandberg, K. W. (1999). Racial profiling. Law Enforcement Technology, 26(6), 62–66.

[cl21302-bib-0090] Weiss, A. , & McGarrell, E. (1996). The impact of increased traffic enforcement on crime. Paper presented at the Annual American Society of Criminology Conference, Chicago.

[cl21302-bib-0091] Yardimci, S. (2009). Kuşatma altında gündelik hayat: Özel güvenlik, kent ve yönetimsellik. Toplum ve Bilim, 115, 226–260.

[cl21302-bib-0092] American Civil Liberties Union . (2010). Bailey, et al., v. City of Philadelphia, et al. Eastern District of Pennsylvania.

[cl21302-bib-0093] Baker, A. L. , & Goldstein, J. (2012). 2 opinions on stop‐and‐frisk report. *New York Times*. http://www.nytimes.com/2012/05/10/nyregion/police-stop-and-frisk-tactic-had-lower-gun-recovery-rate-in-2011.html?_r=1%26ref=nyregion

[cl21302-bib-0094] Borenstein, M. , Hedges, L. V. , Higgins, J. P. T. , & Rothstein, H. R. (2010). A basic introduction to fixed‐effect and random‐effects models for meta‐analysis. Research Synthesis Methods, 1(2), 97–111. 10.1002/jrsm.12 26061376

[cl21302-bib-0095] Brunson, R. K. , & Weitzer, R. (2009). Police relations with black and white youths in different urban neighborhoods. Urban Affairs Review, 44(6), 858–885. 10.1177/1078087408326973

[cl21302-bib-0096] Beccaria, C. (1986). On crimes and punishments (H. Paolucci, Trans.). Macmillan (Originally published 1764).

[cl21302-bib-0097] Bentham, J. (1988). An introduction to the principles of morals and legislation. Prometheus Books (Originally published 1789).

[cl21302-bib-0098] Braga, A. A. , Brunson, R. K. , & Drakulich, K. M. (2019). Race, place, and effective policing. Annual Review of Sociology, 45(1), 535–555. 10.1146/annurev-soc-073018-022541

[cl21302-bib-0099] Braga, A. A. , Turchan, B. , Papachristos, A. V. , & Hureau, D. M. (2019). Hot spots policing of small geographic areas effects on crime. Campbell Systematic Reviews, 15(3), e1046. 10.1002/cl2.1046 PMC8356500 37133274

[cl21302-bib-0100] Braga, A. A. , & Weisburd, D. L. (2020). Does hot spots policing have meaningful impacts on crime? Findings from an alternative approach to estimating effect sizes from place‐based program evaluations. Journal of Quantitative Criminology, 38, 1–22. 10.1007/s10940-020-09481-7

[cl21302-bib-0101] Bushway, S. D. , & Apel, R. J. (2010). Instrumental variables in criminology and criminal justice. In A. R. Piquero , & D. Weisburd (Eds.), Handbook of quantitative criminology (pp. 595–612). Springer.

[cl21302-bib-0102] Campbell, D. T. , & Stanley, J. (1966). Experimental and quasi‐experimental designs for research. Rand McNally.

[cl21302-bib-0103] Cohen, J. (1992). A power primer. Psychological Bulletin, 112(1), 155–159. 19565683 10.1037//0033-2909.112.1.155

[cl21302-bib-0104] Cook, T. , & Campbell, D. T. (1979). Quasi‐experimentation: Design and analysis issues for field settings. Houghton Mifflin.

[cl21302-bib-0105] D'Onfrio, M. (2019). ‘Stop and frisk’ to remain in Philly police arsenal, Ross tells council. *The Philadelphia Tribune*. https://www.phillytrib.com/news/local_news/stop-and-frisk-to-remain-in-philly-police-arsenal-ross-tells-council/article_f6d6b9b3-296f-54fd-8bd8-eb64a5635892.html

[cl21302-bib-0106] Durlauf, S. N. , & Nagin, D. S. (2011). Imprisonment and crime. Criminology & Public Policy, 10(1), 13–54. 10.1111/j.1745-9133.2010.00680.x

[cl21302-bib-0107] Duval, S. , & Tweedie, R. (2000). A nonparametric “trim and fill” method of accounting for publication bias in meta‐analysis. Journal of the American Statistical Association, 95(449), 89–98. 10.2307/2669529

[cl21302-bib-0108] Egger, M. , Smith, G. D. , Schneider, M. , & Minder, C. (1997). Bias in meta‐analysis detected by a simple, graphical test. BMJ, 315(7109), 629–634. 9310563 10.1136/bmj.315.7109.629 PMC2127453

[cl21302-bib-0109] Fagan, J. , & Davies, G. (2000). Street stops and broken Windows: Terry, race, and disorder in New York city. Fordham Urban Law Journal, 28(2), 457–504.

[cl21302-bib-0110] Fisher, Z. , & Tipton, E. (2015). *Robumeta*: *An r‐package for robust variance estimation in meta‐analysis*. http://arxiv.org/abs/1503.02220

[cl21302-bib-0111] Floyd v. City of New York, No. 08 Civ. 1034 (S.D.N.Y. 2013).

[cl21302-bib-0112] Geller, A. , & Fagan, J. (2010). Pot as pretext: Marijuana, race, and the new disorder in New York city street policing. Journal of Empirical Legal Studies, 7(4), 591–633. 10.1111/j.1740-1461.2010.01190.x

[cl21302-bib-0113] Gelman, A. , Fagan, J. , & Kiss, A. (2007). An analysis of the New York city police department's “stop‐and‐frisk” policy in the context of claims of racial bias. Journal of the American Statistical Association, 102(479), 813–823. 10.1198/016214506000001040

[cl21302-bib-0114] Hedges, L. V. (1981). Distribution theory for Glass's estimator of effect size and related estimators. Journal of Educational Statistics, 6(2), 107–128.

[cl21302-bib-0115] Higgins, J. P. , López‐López, J. A. , & Aloe, A. M. (2020). Meta‐regression. In C. H. Schmid , T. Stijnen , & I. White (Eds.), Handbook of meta‐analysis (1st ed., pp. 129–150). Chapman and Hall/CRC. 10.1201/9781315119403

[cl21302-bib-0116] Higgins, J. P. T. , & Thompson, S. G. (2002). Quantifying heterogeneity in a meta‐analysis. Statistics in Medicine, 21(11), 1539–1558. 10.1002/sim.1186 12111919

[cl21302-bib-0117] Higginson, A. , Eggins, E. , Mazerolle, L. , & Stanko, E. (2015). *The Global Policing Database* [Database and Protocol]. http://www.gpd.uq.edu.au

[cl21302-bib-0118] Hinkle, J. C. , Weisburd, D. , Telep, C. W. , & Petersen, K. (2020). Problem‐oriented policing for reducing crime and disorder: An updated systematic review and meta‐analysis. Campbell Systematic Reviews, 16(2), e1089. 10.1002/cl2.1089 PMC8356283 37133256

[cl21302-bib-0119] Illinois v. Wardlow, 528 U.S. 119 (2000).

[cl21302-bib-0120] Johnson, S. D. , Tilley, N. , & Bowers, K. J. (2015). Introducing Emmie: An evidence rating scale to encourage mixed‐method crime prevention synthesis reviews. Journal of Experimental Criminology, 11(3), 459–473. 10.1007/s11292-015-9238-7

[cl21302-bib-0121] Jones‐Brown, D. , Gill, J. , & Trone, J. (2010). Stop, question, & frisk policing practices in New York City: A primer. John Jay College of Criminal Justice.

[cl21302-bib-0122] Koper, C. S. , & Mayo‐Wilson, E. (2006). Police crackdowns on illegal gun carrying: A systematic review of their impact on gun crime. Journal of Experimental Criminology, 2(2), 227–261. 10.1007/s11292-006-9005-x

[cl21302-bib-0123] Koper, C. S. , & Mayo‐Wilson, E. (2012). Police strategies to reduce illegal possession and carrying of firearms: Effects on gun crime. Campbell Systematic Reviews, 8(1), 1–53.

[cl21302-bib-0124] Lachman, P. , La Vigne, N. , & Matthews, A. (2012). Examining law enforcement use of pedestrian stops and searches. In N. La Vigne , P. Lachman , A. Matthews , & S. R. Neusteter (Eds.), Key issues in the police use of pedestrian stops and searches: discussion papers from an urban institute roundtable. Urban Institute.

[cl21302-bib-0125] Lemert, E. M. (1951). Social pathology. McGraw‐Hill.

[cl21302-bib-0126] Lennon, G. (2013). Suspicionless stop and search—Lessons from the Netherlands. Criminal Law Review, 20(2), 178–203.

[cl21302-bib-0127] Lennon, G. (2015). Precautionary tales: Suspicionless counter‐terrorism stop and search. Criminology & Criminal Justice, 15(1), 44–62.

[cl21302-bib-0128] Lennon, G. , & Murray, K. (2018). Under‐regulated and unaccountable? explaining variation in stop and search rates in Scotland, England and Wales. Policing and Society, 28(2), 157–174. 10.1080/10439463.2016.1163359

[cl21302-bib-0129] Levine, H. G. , & Small, D. P. (2008). Marijuana arrest crusade: Racial bias and police policy in New York City, 1997–2007. New York Civil Liberties Union.

[cl21302-bib-0130] Lipsey, M. W. (2003). Those confounded moderators in meta‐analysis: Good, bad, and ugly. The Annals of the American Academy of Political and Social Science, 587(1), 69–81. 10.1177/0002716202250791

[cl21302-bib-0131] Lipsey, M. W. , Puzio, K. , Yun, C. , Hebert, M. A. , Steinka‐Fry, K. , Cole, M. W. , & Busick, M. D. (2012). *Translating the statistical representation of the effects of education interventions into more readily interpretable forms*. National Center for Special Education Research.

[cl21302-bib-0132] Lipsey, M. W. , & Wilson, D. B. (2001). Practical meta‐analysis. Sage Publications, Inc.

[cl21302-bib-0133] Lum, C. , Koper, C. S. , Wilson, D. B. , Stoltz, M. , Goodier, M. , Eggins, E. , Higginson, A. , & Mazerolle, L. (2020). Body‐worn cameras' effects on police officers and citizen behavior: A systematic review. Campbell Systematic Reviews, 16(3), e1112. 10.1002/cl2.1112 PMC8356344 37131919

[cl21302-bib-0134] MacDonald, J. , & Braga, A. A. (2019). Did post‐Floyd et al. reforms reduce racial disparities in NYPD stop, question, and frisk practices? An exploratory analysis using external and internal benchmarks. Justice Quarterly, 36(5), 954–983.

[cl21302-bib-0135] MacDonald, J. M. , & Lattimore, P. K. (2010). Count models in criminology. In A. R. Piquero, & D. Weisburd (Eds.), Handbook of quantitative criminology (pp. 683–698). Springer.

[cl21302-bib-0136] Mazerolle, L. , Antrobus, E. , Bennett, S. , & Tyler, T. R. (2013). Shaping citizen perceptions of police legitimacy: A randomized field trial of procedural justice. Criminology, 51(1), 33–63. 10.1111/j.1745-9125.2012.00289.x

[cl21302-bib-0137] Mazerolle, L. , Cherney, A. , Eggins, E. , Higginson, A. , Hine, L. , & Belton, E. (2020). PROTOCOL: Multiagency programmes with police as a partner for reducing radicalisation to violence. Campbell Systematic Reviews, 16(3), e1110. 10.1002/cl2.1110 PMC8356287 37133270

[cl21302-bib-0138] McNeil, B. (2020). Stop‐and‐frisk in New York, Philadelphia, and Chicago: Slowly approaching an uneasy synthesis or running out of time to justify its freight. Widener Commonwealth Law Review, 29(1), 69–104.

[cl21302-bib-0139] Miller, J. , & D'Souza, A. (2015). Indirect effects of police searches on community attitudes to the police: Resentment or reassurance? British Journal of Criminology, 56(3), 456–478. 10.1093/bjc/azv068

[cl21302-bib-0140] Miller, J. , Gounev, P. , Pap, A. L. , Wagman, D. , Balogi, A. , Bezlov, T. , Simonovits, B. , & Vargha, L. (2008). Racism and police stops: Adapting us and British debates to continental Europe. European Journal of Criminology, 5(2), 161–191. 10.1177/1477370807087641

[cl21302-bib-0141] Nagin, D. S. (2013). Deterrence in the twenty‐first century. Crime and Justice, 42(1), 199–263.

[cl21302-bib-0142] No, D. J. , Amin, M. , Bhutani, T. , & Wu, J. J. (2018). A systematic review of active comparator controlled clinical trials in patients with moderate‐to‐severe psoriasis. Journal of Dermatological Treatment, 29(5), 467–474. 29103334 10.1080/09546634.2017.1402116

[cl21302-bib-0143] R Core Team . (2022). R: A language and environment for statistical computing. R Foundation for Statistical Computing. https://www.Rproject.org/

[cl21302-bib-0144] Ridgeway, G. (2007). Analysis of racial disparities in the New York Police Department's stop, question, and frisk practices. RAND Corporation.

[cl21302-bib-0145] Rosenfeld, R. , & Fornango, R. (2014). The impact of police stops on precinct robbery and burglary rates in New York city, 2003‐2010. Justice Quarterly, 31(1), 96–122. 10.1080/07418825.2012.712152

[cl21302-bib-0146] Saul, J. (2016). America has a stop‐and‐frisk problem. Just look at Philadelphia. *Newsweek*. http://www.newsweek.com/2016/06/10/stop-and-frisk-philadelphia-crisis-reform-police-460951.html

[cl21302-bib-0147] Schur, E. M. (1973). Radical nonintervention: Rethinking the delinquency problem. Prentice Hall.

[cl21302-bib-0148] Shadish, W. R. , Cook, T. D. , & Campbell, D. T. (2002). Experimental and quasi‐experimental designs for generalized causal inference. Houghton Mifflin.

[cl21302-bib-0149] Sherman, L. W. (1990). Police crackdowns: Initial and residual deterrence. Crime and Justice, 12, 1–48. 10.1086/449163

[cl21302-bib-0150] Sherman, L. W. (1993). Defiance, deterrence, and irrelevance: A theory of the criminal sanction. Journal of Research in Crime and Delinquency, 30(4), 445–473.

[cl21302-bib-0151] Skogan, W. G. , & Frydl, K. (Eds.). (2004). Fairness and effectiveness in policing: The evidence. National Academies Press.

[cl21302-bib-0152] Smith, D. C. , & Purtell, R. (2008). *Does stop and frisk stop crime?* Paper presented at the Annual Research Conference of the Association of Public Policy and Management, Los Angeles, CA.

[cl21302-bib-0153] Stafford, M. C. , & Warr, M. (1993). A reconceptualization of general and specific deterrence. Journal of research in crime and delinquency, 30(2), 123–135.

[cl21302-bib-0154] Stein, D. J. , Phillips, K. A. , Bolton, D. , Fulford, K. W. M. , Sadler, J. Z. , & Kendler, K. S. (2010). What is a mental/psychiatric disorder? From DSM‐IV to DSM‐V. Psychological Medicine, 40(11), 1759–1765. 20624327 10.1017/S0033291709992261 PMC3101504

[cl21302-bib-0155] Sterne, J. A. , Hernán, M. A. , Reeves, B. C. , Savović, J. , Berkman, N. D. , Viswanathan, M. , Henry, D. , Altman, D. G. , Ansari, M. T. , Boutron, I. , Carpenter, J. R. , Chan, A. W. , Churchill, R. , Deeks, J. J. , Hróbjartsson, A. , Kirkham, J. , Jüni, P. , Loke, Y. K. , Pigott, T. D. , … Higgins, J. P. (2016). ROBINS‐I: A tool for assessing risk of bias in non‐randomised studies of interventions. BMJ (London), 355, i4919. 10.1136/bmj.i4919 PMC5062054 27733354

[cl21302-bib-0156] Sterne, J. A. C. , Savović, J. , Page, M. J. , Elbers, R. G. , Blencowe, N. S. , Boutron, I. , Cates, C. J. , Cheng, H. Y. , Corbett, M. S. , Eldridge, S. M. , Emberson, J. R. , Hern n, M. A. , Hernán, M. A. , Hopewell, S. , Hróbjartsson, A. , Junqueira, D. R. , Jüni, P. , Kirkham, J. J. , Lasserson, T. , … Higgins, J. P. T. (2019). RoB 2: A revised tool for assessing risk of bias in randomised trials. BMJ (London), 366, l4898. 10.1136/bmj.l4898 31462531

[cl21302-bib-0157] Tanner‐Smith, E. E. , Tipton, E. , & Polanin, J. R. (2016). Handling complex meta‐analytic data structures using robust variance estimates: A tutorial in R. Journal of Developmental and Life‐Course Criminology, 2(1), 85–112. 10.1007/s40865-016-0026-5

[cl21302-bib-0158] Tantry, T. P. , Karanth, H. , Shetty, P. K. , & Kadam, D. (2021). Self‐learning software tools for data analysis in meta‐analysis. Korean Journal of Anesthesiology, 74(5), 459–461. 33677944 10.4097/kja.21080 PMC8497909

[cl21302-bib-0159] Telep, C. W. , Weisburd, D. , Gill, C. E. , Vitter, Z. , & Teichman, D. (2014). Displacement of crime and diffusion of crime control benefits in large‐scale geographic areas: A systematic review. Journal of Experimental Criminology, 10(4), 515–548.

[cl21302-bib-0160] Terkel, A. (2013). Ray Kelly on stop and frisk: ‘no question’ violent crime will rise if program is stopped. The Huffington Post. https://www.huffpost.com/entry/ray-kelly-stop-and-frisk_n_3776035

[cl21302-bib-0161] Telep, C. W. , & Weisburd, D. (2012). What is known about the effectiveness of police practices in reducing crime and disorder? Police Quarterly, 15, 331–357.

[cl21302-bib-0162] Terry v. Ohio, 392 U.S. 1 (1968).

[cl21302-bib-0163] Thompson, M. , & Kahn, K. B. (2016). Mental health, race, and police contact: intersections of risk and trust in the police. Policing: An International Journal of Police Strategies & Management, 39(4), 807–819.

[cl21302-bib-0164] Tiratelli, M. , Quinton, P. , & Bradford, B. (2018). Does stop and search deter crime? Evidence from ten years of London‐wide data. The British Journal of Criminology, 58(5), 1212–1231. 10.1093/bjc/azx085

[cl21302-bib-0165] Viechtbauer, W. (2010). Conducting meta‐analyses in R with the metafor package. Journal of Statistical Software, 36(3), 1–48. http://www.jstatsoft.org/v36/i03/

[cl21302-bib-0166] Wallace, B. C. , Small, K. , Brodley, C. E. , Lau, J. , & Trikalinos, T. A. (2012). *Deploying an interactive machine learning system in an evidence‐based practice center*: *Abstrackr*. Proceedings of the 2nd ACM SIGHIT International Health Informatics Symposium, 819–824.

[cl21302-bib-0167] Weisburd, D. , Lum, C. M. , & Petrosino, A. (2001). Does research design affect study outcomes in criminal justice. The Annals of the American Academy of Political and Social Science, 578(1), 50–70. 10.1177/000271620157800104

[cl21302-bib-0168] Weisburd, D. , & Majmundar, M. K. (Eds.). (2018). Proactive policing: Effects on crime and communities. National Academies of Sciences.

[cl21302-bib-0169] Weisburd, D. , Majmundar, M. K. , Aden, H. , Braga, A. , Bueermann, J. , Cook, P. J. , & Lum, C. (2019). Proactive policing: A summary of the report of The National Academies of Sciences, Engineering, and Medicine. Asian Journal of Criminology, 14(2), 145–177.

[cl21302-bib-0170] Weisburd, D. , Petersen, K. , Zastrow, T. , Davis, R. , Mazerolle, L. , & Eggins, E. (2021). Protocol: Police stops to reduce crime: A systematic review. Campbell Systematic Reviews, 17(2), e1166. 10.1002/cl2.1166 PMC8356359 37051172

[cl21302-bib-0171] Weisburd, D. , Telep, C. W. , & Lawton, B. A. (2014). Could innovations in policing have contributed to the New York city crime drop even in a period of declining police strength?: The case of stop, question and frisk as a hot spots policing strategy. Justice Quarterly, 31(1), 129–153. 10.1080/07418825.2012.754920

[cl21302-bib-0172] Weisburd, D. , Telep, C. W. , Vovak, H. , Zastrow, T. , Braga, A. A. , & Turchan, B. (2022). Reforming the police through procedural justice training: A multicity randomized trial at crime hot spots. *Proceedings of the National Academy of Sciences*, *119*(14), e2118780119. 10.1073/pnas.2118780119 PMC9168920 35344441

[cl21302-bib-0173] Weisburd, D. , Wilson, D. B. , Wooditch, A. , & Britt, C. (2022). Advanced statistics in criminology and criminal justice (5th ed.). Springer International.

[cl21302-bib-0174] White, M. D. , & Fradella, H. F. (2016). Stop and frisk: The use and abuse of a controversial policing tactic. NYU Press.

[cl21302-bib-0175] Wilson, D. B. (2017). Formulas used by the” practical meta‐analysis effect size calculator. Practical Meta‐Analysis. mason.gmu.edu/%7Edwilsonb/downloads/esformulas.pdf

[cl21302-bib-0176] Wilson, D. B. (2022). The relative incident rate ratio effect size for count‐based impact evaluations: When an odds ratio is not an odds ratio. Journal of Quantitative Criminology, 38(2), 323–341.

[cl21302-bib-0177] Wilson, D. B. , Feder, L. , & Olaghere, A. (2021). Court‐mandated interventions for individuals convicted of domestic violence: An updated Campbell systematic review. Campbell Systematic Reviews, 17(1), e1151. 10.1002/cl2.1151 PMC8356297 37133255

