# Supplementary material for: Police stops to reduce crime: A systematic review and meta‐analysis
Source: Campbell Syst Rev. 2023 Jan 10;19(1):e1302. doi: 10.1002/cl2.1302 (PMC9831287; doi:10.1002/cl2.1302)
Supplement: Supplementary file 1 — Supporting information. [file CL2-19-e1302-s001.docx]

Appendix A: GPD Systematic Search Strategy^[[1]](#footnote-1)^

### Search Terms

To ensure optimum sensitivity and specificity, the GPD search strategy utilises a combination of free-text and controlled vocabulary search terms. Because controlled vocabularies and search capabilities vary across databases, the exact combination of search terms and field codes are adapted to each database. Final search syntax for each location will be reported in the final review.

The free-text search terms for the GPD are provided in Table 1 and are grouped by substantive (i.e., some form of policing) and evaluation terminology. Although the search strategy may vary slightly across search locations, it follows a number of general rules:

- Search terms are combined into search strings using Boolean operators “AND” and “OR”. Specifically, terms within each category are combined with “OR”, and categories will be combined with “AND”. For example: (police OR policing OR “law#enforcement”) AND (analy* OR ANCOVA OR ANOVA OR …).
- Compound terms (e.g., law enforcement) are considered single terms in search strings by using quotation marks (i.e., “law*enforcement”) to ensure that the database searches for the entire term rather than separate words.
- Wild cards and truncation codes are used for search terms with multiple iterations from a stem word (e.g., evaluation, evaluate) or spelling variations (e.g., evaluat* or randomi#e).
- If a database has a controlled vocabulary term that is equivalent to “POLICE”, the term is combined in a search string that includes both the policing and evaluation free-text search terms. This approach ensures that the search retrieves documents that do not use policing terms in the title/abstract but have been indexed as being related to policing in the database. An example of this approach is the following search string: (((SU: “POLICE”) OR (TI,AB,KW: police OR policing OR “law*enforcement”)) AND (TI,AB,KW: intervention* OR evaluat* OR compar* OR …)).
- For search locations with limited search functionality, a broad search that uses only the policing free-text terms is implemented.
- Multidisciplinary database searches are limited to relevant disciplines (e.g., include social sciences but exclude physical sciences).
- Search results are refined to exclude specific types of documents that are not suitable for systematic reviews (e.g., newspapers, front/back matter, book reviews).

#### Table A1. Free-text search terms for the GPD systematic search

| **Policing Search Terms** | **Evaluation Search Terms** | | | |
| --- | --- | --- | --- | --- |
| police  policing  “law*enforcement”  constab*  detective*  sheriff* | analy*  ANCOVA  ANOVA  “ABAB design”  “AB design”  baseline  causa*  “chi#square”  coefficient*  “comparison condition*”  “comparison group*”  “control condition*”  “control group*”  correlat*  covariat*  “cross#section*” | data  effect*  efficacy  eval*  experiment*  hypothes*  impact*  intervent*  interview*  longitudinal  MANCOVA  MANOVA  “matched group”  measure*  “meta-analy*”  “odds#ratio* | outcome*  paramet*  “post-test”  posttest  “post test”  predict*  “pre-test”  pretest  program*  “propensity score*”  quantitative  “quasi#experiment*”  questionnaire*  random*  RCT  regress* | result*  “risk#ratio*”  sampl*  “standard deviation*”  statistic*  studies  study  survey*  “systematic review*”  “t#test*”  “time#series”  treatment*  variable*  variance |

### Search Locations

To reduce publication and discipline bias, the GPD search strategy adopts an international scope and involves searching for literature across a number of disciplines (e.g., criminology, law, political science, public health, sociology, social science and social work). The search captures a comprehensive range of published (i.e., journal articles, book chapters, books) and unpublished literature (e.g., working papers, governmental reports, technical reports, conference proceedings, dissertations) by implementing a search strategy across bibliographic/academic, grey literature, and dissertation databases or repositories.

It is noted that there is substantial overlap of the content coverage between many of the databases. Therefore, the *Optimal Searching of Indexing Databases* (OSID) computer program (Neville & Higginson, 2014) has been used to analyse the content crossover for all databases that have accessible content coverage lists. OSID analyses the content coverage and creates a search location solution that provides the most comprehensive coverage via the least number of databases. Another advantage of using OSID when designing a search strategy is the reduction in the number of duplicates that would need to be removed prior to the screening phase. Databases with >10 unique titles are searched in full, whereas databases with ≤10 unique titles were searched only the unique titles and any non-serial content (e.g., reports, conference proceedings). Where a modified search of a database would be more labour-intensive than a full search and export results, a full search of the database is conducted. The final search locations and solutions are reported in Table 2.

#### Table A2. GPD search locations and protocol (January 1^st^ 1950 – December 2019)

| **INDEXED & ACADEMIC DATABASES** |  | **CONTENT COVERAGE FED INTO OSID?** | **FULL OR MODIFIED SEARCH?** | **SEARCH MODIFICATIONS** |
| --- | --- | --- | --- | --- |
| **ProQuest** | Criminal Justice | Yes | Full | None. |
|  | Dissertation and Theses Database Global | Not Available | Modified | Social Sciences subset. |
|  | Political Science | Yes | Full | None. |
|  | Periodical Archive Online | Yes | Full | None. |
|  | Research Library | Yes | Modified | Social Sciences subset. |
|  | Social Science Journals | Yes | Full | None. |
|  | Sociology | Yes | Modified | Search 2 unique journal titles and non-serial content only. |
|  | Applied Social Sciences Index and Abstracts | Yes | Full | None. |
|  | International Bibliography of the Social Sciences | Yes | Full | None. |
|  | Public Affairs Information Service | Yes | Full | None. |
|  | Social Services Abstracts | Yes | Modified | Search 5 unique journal titles and non-serial content only. |
|  | Sociological Abstracts | Yes | Full | None. |
|  | Worldwide Political Sciences Abstracts | Yes | Modified | Search 9 unique journal titles and non-serial content only. |
| **EBSCO** | Academic Search Premier | Yes | Full | None. |
|  | Criminal Justice Abstracts | Yes | Full | None. |
|  | EconLit | Yes | Full | None. |
|  | MEDLINE with Full-Text | Yes | Full | None. |
|  | Social Sciences Full-Text | Yes | Full | None. |
| **OVID** | International Political Science Abstracts | Not Available | Full | None. |
|  | PsycARTICLES | Yes | Modified | Search 4 unique journal titles only. |
|  | PsycEXTRA | Not Available | Full | None. |
|  | PsycINFO | Yes | Full | None. |
|  | Social Work Abstracts | Not Available | Full | None. |
| **Web of Science** | Current Contents Connect – Social and Behavioural Sciences Edition | Yes | Modified | Search 1 unique journal title and non-serial content only. |
|  | Book Citation Index (Social Sciences and Humanities) | Not Available | Full | None. |
|  | Conference Proceedings Citation Index (Social Sciences and Humanities) | Not Available | Full | None. |
|  | Social Science Citation Index | Yes | Full | None. |
| **Informit** | Australian Attorney General Information Service | Yes | Full | None. |
|  | Australian Criminology Database (CINCH) | Yes | Full | None. |
|  | Australian Federal Police Database | Yes | Full | None. |
|  | Australian Public Affairs Full-Text | Yes | Full | None. |
|  | DRUG | Yes | Full | None. |
|  | Health & Society Database | Yes | Modified | Search unique journal titles and non-serial content only. |
|  | Humanities and Social Sciences Collection | Yes | Full | None. |
| **Gale-Cengage** | Expanded Academic ASAP | Yes | Full | None. |
| **STANDALONE & OPEN ACCESS DATABASES** | Cambridge Journals Online | Yes | Modified | Search 4 unique journal titles in Law and Political Science collections and full search of Social Studies collection. |
|  | Directory of Open Access Journals | Yes | Full | None. |
|  | HeinOnline | Yes | Modified | Law Journals Online collection only. |
|  | JSTOR | Yes | Modified | Search unique titles across the Law, Political Science, Public Health, Public Policy, Social Work and Sociology collections only. The Criminal Justice collection had no unique content and so will be excluded from the search. Only 10% of content in this database have abstracts and a full-text search returns >250,000 results because of inability to construct complex search strings. Therefore, a modified search of the unique titles across these collections will be more pragmatic than a full search of the database. |
|  | Oxford Scholarship Online | Yes | Full | None. |
|  | Sage Journals Online and Archive (Sage Premier) | Yes | Modified | Search 5 unique journal titles and non-serial content only. |
|  | ScienceDirect | Yes | Full | None. |
|  | SCOPUS | Yes | Full | None. |
|  | SpringerLink | Yes | Full | Although this database has low uniqueness when combined with the full set of databases, a full search using only the policing search terms will be more pragmatic than a modified search on unique titles because of the restricted search functionality of this database. |
|  | Taylor & Francis Online | Yes | Modified | Although this database has low uniqueness when combined with the full set of databases, a full search using only the policing search terms will be more pragmatic than a modified search on unique titles because of the restricted search functionality of this database. |
|  | Wiley Online Library | Yes | Full | None. |
|  | California Commission on Peace Officer Standards & Training Library | No | Full | None. |
|  | Cochrane Library | No | Full | None. |
|  | CrimeSolutions.gov | No | Full | None. |
|  | Database of Abstracts of Reviews of Effectiveness (DARE) | No | Full | None. |
|  | FBI – The Fault (Reports and Publications) | No | Full | None. |
|  | Evidence-Based Policing Matrix | No | Full | None. |
|  | International Initiative for Impact Evaluation Database (3ie) | No | Full | None. |
|  | National Criminal Justice Reference Service | No | Full | None. |
|  | Safety Lit Database | No | Full | None. |
|  | Australian Institute of Criminology | No | Full | None. |
|  | Bureau of Police Research and Development (India) | No | Full | None. |
|  | Canadian Police Research Catalogue | No | Full | None. |
|  | Centre for Problem-Oriented Policing | No | Full | None. |
|  | College of Policing (including POLKA and Crime Reduction Toolkit) | No | Full | None. |
|  | European Police College (CEPOL) | No | Full | None. |
|  | Evidence for Policy and Practice Information and Coordinating Centre | No | Full | None. |
|  | National Research Institute of Police Science (Japanese) | No | Full | None. |
|  | Office of Community Oriented Policing Services | No | Full | None. |
|  | Police Executive Research Forum (US) | No | Full | None. |
|  | Police Foundation (US) | No | Full | None. |
|  | Tasmania Institute of Law Enforcement Studies (Australia) | No | Full | None. |
|  | Policing Online Information System (POLIS, Europe) | No | Full | None. |
|  | Scottish Institute for Policing Research | No | Full | None. |
|  | Centre of Excellence in Policing and Security (Australian, now archived) | No | Full | None. |

Appendix B: GPD Systematic Compilation Strategy

### Inclusion Criteria

Each record captured by the GPD systematic search must satisfy all inclusion criteria to be included in the GPD: timeframe, intervention and research design. There are no restrictions applied to the types of outcomes, participants, settings or languages considered eligible for inclusion in the GPD.

#### Types of interventions

Each document must contain an impact evaluation of a policing intervention. Policing interventions are defined as some kind of a strategy, program, technique, approach, activity, campaign, training, directive, or funding/organisational change that involves police in some way (other agencies or organisations can be involved). Police involvement is broadly defined as:

- Police initiation, development or leadership
- Police are recipients of the intervention or the intervention is related, focused or targeted to police practices
- Delivery or implementation of the intervention by police

#### Types of study designs

The GPD includes quantitative impact evaluations of policing interventions that utilise randomised experimental (e.g., RCTs) or quasi-experimental evaluation designs with a valid comparison group that does not receive the intervention. The GPD includes designs where the comparison group receives ‘business-as-usual’ policing, no intervention or an alternative intervention (treatment-treatment designs).

The specific list of research designs included in the GPD are as follows:

- Systematic reviews with or without meta-analyses
- Cross-over designs
- Cost-benefit analyses
- Regression discontinuity designs
- Designs using multivariate controls (e.g., multiple regression)
- Matched control group designs with or without pre-intervention baseline measures (propensity or statistically matched)
- Unmatched control group designs with pre-post intervention measures which allow for difference-in-difference analysis
- Unmatched control group designs without pre-intervention measures where the control group has face validity
- Short interrupted time-series designs with control group (less than 25 pre- and 25 post-intervention observations)
- Long interrupted time-series designs with or without a control group (≥25 pre- and post-intervention observations)
- Raw unadjusted correlational designs where the variation in the level of the intervention is compared to the variation in the level of the outcome

The GPD excludes single group designs with pre- and post-intervention measures as these designs are highly subject to bias and threats to internal validity.

### Systematic Screening

To establish eligibility, records captured by the GPD search progress through a series of systematic stages which are summarised in Figure C1, with additional detail provided in the following subsections. All research staff working on the GPD undergo standardised training before beginning work within any of the stages detailed below. Staff then complete short training simulations to enable an assessment of their understanding of the GPD protocols and highlight any areas for additional training. In addition, random samples of each staff’s work are regularly cross-checked to ensure adherence to protocols. Disagreements about screening decisions between staff are mediated by either the project manager or GPD chief investigators.

#### Title and abstract screening

After removing duplicates, the title and abstract of records captured by the GPD systematic search is screened by trained research staff to identify potentially eligible research that satisfies the following criteria:

- Document is dated between 1950 – present
- Document is unique (i.e., not a duplicate)
- Document is about police or policing
- Document is an eligible document type (e.g., not a book review)

Records are excluded if the answer to any one of the criteria is unambiguously ‘No’, and will be classified as potentially eligible otherwise. Records classified as eligible at the title and abstract screening stage progress to full-text document retrieval and screening stages.

#### Full-text eligibility screening

Wherever possible, a full-text electronic version of an eligible record is imported into *SysReview* (review management software; Neville & Higginson, 2014). For records without an electronic version, a hardcopy of the record is located to enable full-text eligibility screening. The full-text of each document is screened to identify studies that satisfy the following criteria:

- Document is dated between 1950 – present
- Document is unique
- Document reports a quantitative statistical comparison
- Document reports on policing evaluation
- Document reports in a quantitative impact evaluation of a policing intervention
- Evaluation uses an eligible research design

***Figure B1.*** GPD systematic compilation process

Appendix C: additional Search strategies

**EBSCO Search**

**Timeframe:** 1/1/2020 – 12/31/2021

**Conducted:** 3/18/2022

**Total Results:** 272

**Databases:**

- Criminal Justice Abstracts
- National Criminal Justice Reference Service Abstracts
- SocINDEX with Full Text

**Search String:**

(TI(police OR policing OR "law*enforcement" OR constab* OR detective* OR

sheriff*) OR AB(police OR policing OR "law*enforcement" OR constab* OR detective* OR sheriff*)) AND (TI(analy* OR ANCOVA OR ANOVA OR "ABAB design" OR "AB design" OR baseline OR causa* OR "chi#square" OR coefficient* OR "comparison condition*" OR "comparison group*" OR "control condition*" OR "control group*" OR correlat* OR covariat* OR "cross#section*" OR data OR effect* OR efficacy OR eval* OR experiment* OR hypothes* OR impact* OR intervent* OR interview* OR longitudinal OR MANCOVA OR MANOVA OR "matched group" OR measure* OR "meta-analy*" OR "odds#ratio*" OR outcome* OR paramet* OR "post-test" OR posttest OR "post test" OR predict* OR "pre-test" OR pretest OR program* OR "propensity score*" OR quantitative OR "quasi#experiment*" OR questionnaire* OR random* OR RCT OR regress* OR result* OR "risk#ratio*" OR sampl* OR "standard deviation*" OR statistic* OR studies OR study OR survey* OR "systematic review*" OR "t#test*" OR "time#series" OR treatment* OR variable* OR variance) OR AB(analy* OR ANCOVA OR ANOVA OR "ABAB design" OR "AB design" OR baseline OR causa* OR "chi#square" OR coefficient* OR "comparison condition*" OR "comparison group*" OR "control condition*" OR "control group*" OR correlat* OR covariat* OR "cross#section*" OR data OR effect* OR efficacy OR eval* OR experiment* OR hypothes* OR impact* OR intervent* OR interview* OR longitudinal OR MANCOVA OR MANOVA OR "matched group" OR measure* OR "meta-analy*" OR "odds#ratio*" OR outcome* OR paramet* OR "post-test" OR posttest OR "post test" OR predict* OR "pre-test" OR pretest OR program* OR "propensity score*" OR quantitative OR "quasi#experiment*" OR questionnaire* OR random* OR RCT OR regress* OR result* OR "risk#ratio*" OR sampl* OR "standard deviation*" OR statistic* OR studies OR study OR survey* OR "systematic review*" OR "t#test*" OR "time#series" OR treatment* OR variable* OR variance)) AND (TI(stop* OR SQF OR frisk OR search* OR "street pop*" OR "street check*" OR "street-check*") OR AB(stop* OR SQF OR frisk OR search* OR "street pop*" OR "street check*" OR "streetcheck*"))

**ProQuest Search**

**Timeframe:** 1/1/2020 – 12/31/2021

**Conducted:** 3/18/2022

**Total Results**: 307

**Databases:**

- Criminal Justice Database
- PAIS Index
- Policy File Index
- ProQuest Dissertations and Theses Global
- Sociological Abstracts
- Sociology Database

**Search String:**

((TI(police OR policing OR "law*enforcement" OR constab* OR detective* OR

sheriff*) OR AB(police OR policing OR "law*enforcement" OR constab* OR detective* OR sheriff*)) AND (TI(analy* OR ANCOVA OR ANOVA OR "ABAB design" OR "AB design" OR baseline OR causa* OR "chi#square" OR coefficient* OR "comparison condition*" OR "comparison group*" OR "control condition*" OR "control group*" OR correlat* OR covariat* OR "cross#section*" OR data OR effect* OR efficacy OR eval* OR experiment* OR hypothes* OR impact* OR intervent* OR interview* OR longitudinal OR MANCOVA OR MANOVA OR "matched group" OR measure* OR "meta-analy*" OR "odds#ratio*" OR outcome* OR paramet* OR "post-test" OR posttest OR "post test" OR predict* OR "pre-test" OR pretest OR program* OR "propensity score*" OR quantitative OR "quasi#experiment*" OR questionnaire* OR random* OR RCT OR regress* OR result* OR "risk#ratio*" OR sampl* OR "standard deviation*" OR statistic* OR studies OR study OR survey* OR "systematic review*" OR "t#test*" OR "time#series" OR treatment* OR variable* OR variance) OR AB(analy* OR ANCOVA OR ANOVA OR "ABAB design" OR "AB design" OR baseline OR causa* OR "chi#square" OR coefficient* OR "comparison condition*" OR "comparison group*" OR "control condition*" OR "control group*" OR correlat* OR covariat* OR "cross#section*" OR data OR effect* OR efficacy OR eval* OR experiment* OR hypothes* OR impact* OR intervent* OR interview* OR longitudinal OR MANCOVA OR MANOVA OR "matched group" OR measure* OR "meta-analy*" OR "odds#ratio*" OR outcome* OR paramet* OR "post-test" OR posttest OR "post test" OR predict* OR "pre-test" OR pretest OR program* OR "propensity score*" OR quantitative OR "quasi#experiment*" OR questionnaire* OR random* OR RCT OR regress* OR result* OR "risk#ratio*" OR sampl* OR "standard deviation*" OR statistic* OR studies OR study OR survey* OR "systematic review*" OR "t#test*" OR "time#series" OR treatment* OR variable* OR variance)) AND (TI(stop* OR SQF OR frisk OR search* OR "street pop*" OR "street check*" OR "street-check*") OR AB(stop* OR SQF OR frisk OR search* OR "street pop*" OR "street check*" OR "streetcheck*")))

**Limiters:** Excluded trade journals and magazines

**CiNii Articles Search**

**Timeframe:** 1970-2021

**Conducted:** 3/10/2022

**Notes:** No title, abstract, or subject limiters used (did not appear to be available)

**Total Results:** 48

**Search String:**

- (stop* OR SQF OR frisk OR search* OR "street pop*" OR "street check*" OR "street-check*") AND (police OR policing OR "law*enforcement" OR constab* OR detective* OR sheriff*)

**DBpia Search**

**Timeframe:** No date limiters available (by default 2000-2022)

**Conducted:** 3/10/2022

**Notes:** No title, abstract, or subject limiters used (shorter search string the only one that would return results)

**Total Results:** 15

**Search String:**

- (stop* OR frisk) AND (police OR policing)

**Index Islamicus Search**

**Timeframe:** 1970-2021

**Conducted:** 3/10/2022

**Notes:** No title, abstract or subject limiters used

**Total Results:** 4

**Search String:**

- (stop* OR SQF OR frisk OR search* OR "street pop*" OR "street check*" OR "street-check*") AND (police OR policing OR "law*enforcement" OR constab* OR detective* OR sheriff*)

**Middle Eastern and Central Asian Studies Search**

**Timeframe:** 1970-2021

**Conducted:** 3/10/2022

**Notes:** Search limited to title or abstract fields

**Total Results:** 146

**Search String**

- (stop* OR SQF OR frisk OR search* OR "street pop*" OR "street check*" OR "street-check*") AND (police OR policing OR "law*enforcement" OR constab* OR detective* OR sheriff*)

**Historical Abstracts Search**

**Timeframe:** 1970-2021

**Conducted:** 3/10/2022

**Notes:** Search limited to title or abstract fields

**Total Results:** 182

**Search String**

- (stop* OR SQF OR frisk OR search* OR "street pop*" OR "street check*" OR "street-check*") AND (police OR policing OR "law*enforcement" OR constab* OR detective* OR sheriff*)

**Journals with 2019-2021 Volumes Reviewed**

*Cambridge Journal of Evidence-Based Policing, Criminology, Criminology and Public Policy, Justice Quarterly, Journal of Research in Crime and Delinquency, Journal of Criminal Justice, Police Quarterly, Policing, Police Practice and Research, British Journal of Criminology, Journal of Quantitative Criminology, Crime and Delinquency, Journal of Criminal Law and Criminology, Policing and Society.*

Appendix D: pedestrian stops coding sheet

**Reference Information**

1. Document ID: __ __ __ __

2. Study author(s): ____________________

3a. Are multiple publications/reports associated with this intervention?

1. Yes

2. No

3b. If yes, list secondary/additional study author(s):___________

3c. Study title(s) associated with intervention: _______________________

4a. Primary publication type(s): ______

1. Book

2. Book chapter

3. Journal article (peer reviewed)

4. Thesis or doctoral dissertation

5. Government report (state/local)

6. Government report (federal)

7. Police department report

8. Technical report

9. Conference paper

10. Award submission

11. Other (specify)

4b. Specify (Other)_____________________

5. Publication date (year): ______________

6a. Journal Name: ____________________

6b. Journal Volume: _______________

6c. Journal Issue: ____________

7. Date range of research (i.e., time span covered by research from pre to post-intervention follow-up):

Start: ____________

Finish: ____________

8. Source(s) of funding for study: ___________________

9. Country of publication: ___________________

10. Date coded: ___________

11. Coder’s Initials: __ __ __

**Describing the Intervention(s)**

12a. Were pedestrian stops the only strategy applied during the intervention?

1. Yes

2. No

3. Unclear

12b. Which of the following best describes the type of intervention? (Select all that apply)

1. Police crackdown using pedestrian stops as a major component

2. Hot spot policing intervention using pedestrian stops as a major component

3. Directed patrol intervention using pedestrian stops as a major component

4. Random preventative patrol intervention using pedestrian stops as a major component

5. Disorder policing intervention using pedestrian stops as a major component

6. Problem-oriented policing intervention using pedestrian stops as a major component

7. Other (specify)

12c. Specify (Other) _____________

13a. What was the target of the pedestrian stops? (Select all that apply)

1. Weapons
2. Violent crime generally
3. Drugs
4. Gangs
5. General crime at specific places

6. N/A (no specific target)

7. Other (specify)

13b. Specify (Other) _______________

14a. What other enforcement actions took place during the intervention? (Select all that apply)______

1. Search warrants/raids

2. Disorder enforcement

3. POP approaches

4. Misdemeanor arrests

5. Felony arrests

6. Traffic stops/vehicle checks

7. No enforcement activities noted

8. Other

14b. Specify (Other) ___________

15. Describe the nature of the intervention, including the rationale for using stops and the specific activities that officers were told to engage in (if provided). *For individual-level studies, describe how stops were defined.* _________________________________________________________________________________________________________________________________________________________________________________________________________________________

16a. Which of the following best describe the dosage of the police intervention?

(check all that apply)

1. Officers from patrol assigned to conduct stops full-time

2. Officers from patrol assigned to conduct stops part-time

3. Officers from special unit assigned to conduct stops full-time

4. Officers from special unit assigned to conduct stops part-time

5. Officers from patrol conduct stops during downtime (e.g., between calls)

6. Officers from special unit conduct stops during downtime

7. Other (specify)

16b. Specify (Other) ___________________

16c. Briefly describe the intensity/dosage of the police intervention (i.e. the amount of street stops and/or searches that were conducted).

________________________________________________________________

________________________________________________________________

________________________________________________________________

16d. Please provide a “baseline”, for example per officer, per shift etc.

________________________________________________________________

________________________________________________________________

17a. At what level of the police department was the response implemented? _____

1. Entire department/all officers involved

2. Certain precincts/districts involved

3. Special unit (e.g., hot spots unit) involved

4. Select few officers in specific area involved

5. Other (specify)

6. N/A (not mentioned)

17b. Specify (Other)___________________

18a. At what type of crime or disorder is the intervention directed? (select all that apply)

1. All offense types

2. Property offenses

3. Violent offenses

4. Drug offenses

5. Weapons offenses

6. Sexual offenses

7. Disorder offenses

8. Other (specify)

18b. Specify (Other ___________________________________

19a. At what geographic area was the pedestrian stops intervention targeted? _______

1. Micro place (e.g., hot spot)

2. Meso area (e.g., neighborhoods/police beats)

3. Large area (e.g., entire city)

4. Other (specify)

19b. Specify (Other) ___________________________

20a. Was the demographic (i.e. racial/ethnic and gender) composition of the targeted area discussed?

1. Yes

2. No

20b. If yes, briefly describe the information provided and the page number is it provided on. _________________________

21a. Was the socioeconomic status of the targeted area discussed?

1. Yes

2. No

21b. If yes, briefly describe the information provided and the page number it is provided on.

__________________________

22a. What was the nature of the comparison or control condition?

1. Standard policing

2. No treatment

3. Alternative policing intervention

4. Other (specify)

22b. Specify (Other) ______________________

22c. If an alternative policing intervention was employed in the control/comparison area, which of the following best describes the type of intervention? (Select all that apply)

1. Hot spot policing intervention

2. Directed patrol intervention

3. Random preventative patrol intervention

4. Problem-oriented policing intervention

5. Community policing intervention

6. Other (specify)

22d. Specify (Other) ___________________________

23. Briefly describe the nature of the comparison or control condition

______________________________________________________

______________________________________________________

24a. Was the demographic (i.e. racial/ethnic and gender) composition of the control/comparison area discussed?

1. Yes

2. No

24b. If yes, briefly describe the information provided and the page number is it provided on. _________________________

25a. Was the socioeconomic status of the control/comparison area discussed?

1. Yes

2. No

25b. If yes, briefly describe the information provided and the page number it is provided on.

__________________________

25c. Was the demographic or socioeconomic information for the full sample discussed?

__________________________

25d. If yes, briefly describe the information provided and the page number it is on

___________________________

***Implementation of Intervention***

26. What did the evaluation indicate about the implementation of the response? _____

1. There were no reported implementation issues

2. There were minor implementation issues

3. There were more substantial implementation issues

4. There were major implementation issues/the project was not implemented as planned

5. Unclear/no process evaluation included

27. If the process evaluation indicated there were problems with implementation of the response, describe these problems:

__________________________________________________________________________________________________________________________________________________________________________________________________________________________________________

***Location of the intervention***

28. Country where study was conducted: __________________

29. City (and state/province, if applicable) where study was conducted: _________________

*The following questions refer to the area receiving treatment:*

30a. Geographic area receiving treatment (may differ from area originally targeted): ______

1. Micro place (street segments/blocks)

2. Neighborhood/police beat

3. Police district/precinct

4. Entire city

5. Other (specify)

30b. Specify (Other)___________________

31. What is the exact geographic area receiving treatment? ______________________________________________________________________________

32. Describe any information provided about the nature of the site selection.

_______________________________________________________________________________

*The following refer to the area not receiving treatment*

33a. Geographic area NOT receiving treatment: ______

1. Micro place (street segments/blocks)

2. Neighborhood/police beat

3. Police district/precinct

4. Entire city

5. Other (specify)

33b. Specify (Other)___________________

34. What is the exact geographic area not receiving treatment? ____________________________________________________________________________

35. Described any information provided about the nature of the control/comparison group selection

_____________________________________________________________________________

**Methodology/Research design:**

36a. Length of pre-intervention study period______

36b. Length of intervention study period_________

36c. Length of post-intervention study period______

37. Is there a secondary follow-up period for this intervention? (*Note that for each additional follow-up period, a separate coding sheet is required)*

1. Yes

2. No

38a. Type of study: _____

1. Randomized experiment

2. Block randomized experiment

3. Nonequivalent control group (quasi-experimental)

4. Other (specify)

38b. Specify (Other)___________________

39a. What process was used to assign study units to treatment or comparison conditions?

1. Simple random assignment
2. Random assignment from within blocks or pairs
3. Identification of matching areas or persons through regression analyses (e.g. propensity score matching)
4. Statistical tests of mean differences among demographic and other relevant variables
5. Comparison of descriptive statistics with no statistical test of differences across groups
6. Comparison to the rest of a jurisdiction or population that did not receive the treatment
7. Other (specify)

39b. Specify (Other)___________________

40a. Was multiple outcome measurement used?

1. Yes analyzed multiple time periods in a single analysis (e.g. statistical modelling with multiple dummy variables for time).

2. Yes conducted a time series statistical analysis

3. No (e.g. single pre/post observations)

4. Unclear

5. Other (Specify)

40b. Specify (other) ____________________________

40c. Explain any other measures that were taken to control for the influence of potentially confounding variables or to strengthen the internal validity of the study’s results. ____________________________________________________________________________________________________________________________________________________

41a. Were any sources of non-equivalence or bias reported or implied in the application of the intervention or its analysis (i.e. threats to internal validity)?

1. Yes

2. No

41b. If yes, what sources of nonequivalence or bias were identified? (check all that apply and explain)

1. Extraneous events or factors occurring during the intervention period; historical artifacts

2. Selection of treatment area based on high baseline crime rate

3. Measurement confounds (measure changes over time)

4. Differential attrition, breakdown of randomization, or contamination of control group

5. Pre-test analyses indicated nonequivalence between treatment and control groups

6. Statistical analyses failed to adjust for nonequivalence at baseline

7. Inappropriate statistical analysis for design

8. Any outcomes measured by reporters that did not have corresponding outcome measures in the results

9. Other threats to internal validity (specify)

41c. Explain any yes responses checked in 31b.

______________________________________________________________________________________________________________________________________________________________________________________________________

41d. If yes, did the researchers discuss the implications of the bias for their findings?

1. Yes

2. No

42. Did the researcher assess the quality of the data collected?

1. Yes

2. No

43a. Did the researcher(s) express any concerns over the quality of the data?

1. Yes

2. No

43b. If yes, explain ____________________________________________________________________________________________________________________________________________________________

***Outcomes reported*** *(Note that for each outcome, a separate coding sheet is required. Outcomes may include crime/disorder, police-citizen violence, police misbehaviour, community outcomes, or health-related outcomes)*

44. How many outcomes are reported in the study? _____

45. What is the specific outcome recorded on this coding sheet?

_______________________________________________________________

46. Was it the primary outcome of the study? _______

1. Yes

2. No

3. Can’t tell/researcher did not prioritize outcomes

47a. Was this initially intended as an outcome of the study? ______

1. Yes

2. No (explain)

3. Can’t tell

47b. If no, explain why:

__________________________________________________________________

__________________________________________________________________

__________________________________________________________________

***Dependent Variable***

48a. What type of data was used to measure the outcome covered on this coding sheet? ____

1. Official data

2. Researcher observations

3. Self-report surveys

4. Other (specify)

48b. Specify (Other)___________________

49a. If official data was used, what specific type(s) of data were used? (Select all that apply)

1. Calls for service (911 calls)

2. Arrests

3. Incident reports

4. Level of citizen complaints

5. Use of force reports

6. Official medical information

7. Other (specify)

8. N/A (official data not used)

49b. Specify (Other)___________________

50a. If researcher observations were used, what types of observations were taken? (Select all that apply)

1. Physical observations (e.g. observed urban blight, such as trash, graffiti)

2. Social observations (e.g. observed disorder, such as loitering, public drinking)

3. Other observations (specify)

4. N/A (researcher observations not used)

50b. Specify (Other)___________________

51a. If self-report surveys were used, who was surveyed? (Select all that apply)

1. Residents/community members

2. Business owners

3. Elected officials

4. Government/social service agencies

5. Other (specify)

6. N/A (self-report surveys not used)

51b. Specify (Other)___________________

**Effect size/Reports of statistical significance**

***Sample size***

52. Based on the unit of analysis for this outcome, what is the total sample size in the analysis? ________

53. What is the total sample size of the treatment group (group that receives the response)? _______

54. What is the total sample size of the control group (if applicable)? _____

55a. Was attrition a problem in the analysis for this outcome?

1. Yes

2. No

55b. If attrition was a problem, provide details (e. g. how many cases lost and why they were lost).

__________________________________________________________________________________________________________________________________________________________________________________________________________________________________________

56a. What do the sample sizes above refer to?

1. Crimes

2. People

3. Geographic areas

4. Other (specify)

56b. Specify (other) ________________

57a. Did the study report the number of stops or searches conducted?

1. Yes

2. No/unclear

57b. If yes, how many stops were conducted for the treatment group? ________

57c. If yes, how many searches were conducted for the treatment group? _______

57d. If yes, how many stops were conducted for the control group? _________

57e. If yes, how many searches were conducted for the control group? _________

58a. Was demographic information provided for the individuals stopped/searched?

1. Yes

2. No

58b. If yes, indicate the page numbers on which these data are provided ________

59a. Did the study report the number of seizures (weapon or drug) that occurred?

1. Yes

2. No

59b. If yes, how many weapon seizures occurred in the treatment group? __________

59c. If yes, how many drug seizures occurred in the treatment group? ____________

59d. If yes, how many weapon seizures occurred in the control group? ____________

59e. If yes, how many drug seizures occurred in the control group? ______________

***Effect Size Data***

60. Raw difference favors (i.e. shows more success for):

1. Treatment group

2. Control group

3. Neither (exactly equal)

9. Cannot tell (or statistically insignificant report only)

61a. Did a test of statistical significance indicate statistically significant differences between either the control and treatment groups or the pre and post tested treatment group for the current outcome? ____

1. Yes

2. No

3. N/A (no testing completed/reported)

61b. If yes, what was the level of statistical significance for the current outcome

1. .01
2. .05
3. .1
4. N/A (no p-value reported)

61c. Type of significance test used for the current outcome

1. One-tailed
2. Two-tailed

9. Cannot tell (unclear from text or not reported)

62a. Was an effect size reported?

1. Yes

2. No

62b. What type of effect size was reported? _______________

63. If yes, what was the effect size? ______

64. If yes, page number where effect size data is found ________

65. If no, is there data available to calculate an effect size?

1. Yes

2. No

66a. Type of data effect size can be calculated from (Select all that apply):

1. Means and standard deviations

2. *t*-value or *F*-value

3. Chi-square (df=1)

4. Frequencies or proportions

5. Incident rate ratio, odds ratio, or risk ratio

6. Other (specify)

66b. Specify (other) _________

*Pre-post Study Counts*

67a. Pre-period number of events for current outcome in target area _______

67b. During intervention-period number of events for current outcome in target area ______

67c. Post-period number of events for current outcome in target area ______

67d. Pre-period number of events for current outcome in comparison area _______

67e. During intervention-period number of events for current outcome in comparison area _____

67f. Post-period number of events for current outcome in comparison area ______

67g. Did the evaluation control for validity by using multivariate methods (i.e. regression) to assess the impact of the program?

1. Yes

2. No

67h. If yes, did this analysis find that the intervention reduced the outcome at a statistically significant level?

1. Yes

2. No

3. N/A

*Means and Standard Deviations*

68a. Pre-period treatment group mean. _____

68b. During intervention-period treatment group mean_____

68c. Post-period treatment group mean_____

68d. Pre-period control group mean. _____

68e. During intervention-period control group mean_____

68f. Post-period control group mean_____

69a. Pre-period treatment group standard deviation. _____

69b. During intervention-period treatment group standard deviation. ____

69c. Post-period treatment group standard deviation. _____

69d. Pre-period control group standard deviation. _____

69e. During intervention-period control group standard deviation. _____

69f. Post-period control group standard deviation. ______

*Proportions or frequencies*

70a. *n* of treatment group with a successful outcome. _____

70b. *n* of control group with a successful outcome. _____

71a. Proportion of treatment group with a successful outcome. _____

71b. Proportion of control group with a successful outcome. _____

*Significance Tests*

72a. *t*-value _____

72b. *F*-value _____

72c. Chi-square value (*df*=1) _____

72d. IRR value _______

72e. OR value _______

72f. RR (relative risk) value _______

*Calculated Effect Size*

73a. Effect size ______

73b. Standard error of effect size _____

**Conclusions made by the author(s)**

*Note that the following questions refer to conclusions about the effectiveness of the intervention. Desirable changes could be increases or decreases in the level of an outcome*

74. Conclusion about the impact of the intervention on crime/disorder? _____

1. Authors conclude intervention associated with crime decline

2. Authors conclude intervention not associated with a crime decline

3. Mixed across crime/disorder type

4. Unclear/no conclusion stated by authors

75. Conclusion about the impact of the intervention on violence in police/citizen encounters? _____

1. Authors conclude intervention associated with decline in violence

2. Authors conclude intervention not associated with decline in violence

3. Mixed evidence/no change

4. Unclear/no conclusion stated by authors

76. Conclusion about the impact of the intervention on officer misbehavior? ________

1. Authors conclude intervention associated with decline in misbehavior

2. Authors conclude intervention not associated with decline in misbehavior

3. Mixed evidence/no change

4. Unclear/no conclusion stated by authors

77. Conclusion about the impact of the intervention on community outcomes? ________

1. Authors conclude intervention associated with improvements in community outcomes

2. Authors conclude intervention not associated with improvement in community outcomes

3. Mixed evidence across outcome type

4. Unclear/no conclusion stated by authors

78. Did the assessment find evidence of a geographic displacement of crime? ______

1. Yes

2. No

3. No statistical test but authors claim evidence of displacement

4. No statistical test but authors claim no evidence of displacement

5. Mixed across crime/disorder type

6. Not tested

79a. Did the assessment find evidence of other non-geographic types of displacement of crime? _____

1. Yes

2. No

3. No statistical test but authors claim evidence of displacement

4. No statistical test but authors claim no evidence of displacement

5. Mixed across crime/disorder type

6. Not tested

79b. If yes, specify what types of displacement were found

______________________________________________________________________________________________________________________________________________________

80. Additional notes about conclusions:

____________________________________________________________________________________________________________________________________________________________

81. Additional notes about study:

____________________________________________________________________________________________________________________________________________________________

Appendix E: narrative summaries of included studies

**Alderden et al. (2011)**

Alderden et al. (2011) evaluated whether Chicago’s decline in violent crime from 2000-2007 was the result of the Chicago Police Department’s (CPD) Deployment Operations Center (DOC) process. The DOC analyzed crime and intelligence data to identify violent crime hot spots, which guided decisions for strategically deploying specialized units to the hot spot areas in order to suppress gang, drug, and gun crime. Alderden et al. (2011) qualitatively evaluated how CPD personnel understood and implemented the DOC process and quantitatively examined whether the DOC process impacted crime reduction in Chicago. Homicides, gang-related homicides, gun-related homicides, drive-by shootings, aggravated battery with a gun, and violent crimes committed outdoors were the violent crime indicators Alderden et al. (2011) analyzed to assess the impact of the DOC process on reducing violent crime in 281 police beats, which notably were larger than the typical hot spot defined in other criminological literature. Alderden et al. (2011) found that CPD successfully implemented the DOC process as it was designed. However, the DOC logic model was not associated with significant reductions in violent crime. Alderden et al. (2011) suggested these findings resulted from frequent movement of treatment deployment areas and crime was more easily suppressed in the smaller deployment areas, perhaps because resources were diffused in larger deployment areas resulting in less targeted crime prevention.

**Baćak & Apel (2020)**

Baćak and Apel (2020) examined the relationship between the health and wellbeing of individuals with and without law enforcement contact over the past two years. Baćak and Apel (2020) analyzed a nationally representative sample of data from 26 countries participating in the 2010 round of the European Social Survey to examine these health outcomes. They found that participants reporting that they experienced some form of police contact (such as being approached, stopped, or otherwise contacted) within the last two years were associated with poorer health outcomes, especially when participants reported their experience with the police as unsatisfactory.

**Baćak & Apel (2021)**

Baćak and Apel (2021) compared police-initiated contact and contact satisfaction between post-communist and democratic European regions to examine how policing and political history affected police-public interactions long-term. Using data from the 2010 European Social Survey, in which 26 European countries participated, Baćak and Apel (2021) found that police-initiated contact and satisfaction with those contacts reported in the prior two years were higher in established democracies compared to post-communist countries. Respondents living in post-communist countries reported significantly lower prevalence of and satisfaction with police contact. Baćak and Apel (2021) also found that the associations between police contact and attitudes about police fairness and legitimacy were stronger in post-communist countries. Baćak and Apel (2021) concluded that history and context can help explain how police-public interactions shape public opinion about police. However, the authors warned that causal relationships could not be determined given the exploratory and cross-sectional nature of the study.

**Baćak & Nowotny (2020)**

Baćak and Nowotny (2020) examined young adults aged 18-26 who had or had not been stopped by the police in their lifetime and their associated depressive symptoms using the National Longitudinal Survey of Adolescent to Adult Health. Baćak and Nowotny (2020) found that Black and White adults that had been stopped by police (with the exception of minor traffic violations) had higher levels of depressive symptoms compared to those who had never been stopped. Even when controlling for prior depressive symptoms, Baćak and Nowotny (2020) found that the number of police stops someone experienced was associated with more depressive symptoms in their nationally representative sample, with data collected through three waves in 1994-1995, 1996, and 2001-2002. However, Black adults being stopped by police were associated with higher levels of depressive symptoms when compared to White adults that had experienced a police stop in the full multivariate model.

**Boydstun (1975)**

The San Diego Police Department (SDPD) Field Interrogation Project, sponsored by the Police Foundation, examined the efficacy of the SDPD’s field interrogation program. The SDPD defined a field interrogation as a patrol officer-initiated contact (including stopping, questioning, and sometimes searching) with a citizen when the officer had a reasonable suspicion that a crime had been committed or was about to occur. Researchers studied the impact that (1) the continuation of field interrogation practices, (2) the implementation of field interrogation practices by specially trained officers to ease police-community relations, and (3) the cessation of all field interrogation activities had on reported suppressible crimes, total arrest rates, and police-community relations. The suppressible crimes the SDPD believed could be influenced by patrol activities included robbery, burglary, grand theft, petty theft, auto theft, assault/battery, sex crimes, and malicious mischief/disturbances. The experiment lasted for nine months to compare the outcomes in the control area (with field interrogations policing as usual), the field interrogation area (patrolled by specially trained officers), and the no-field interrogation area (in which all field interrogations were suspended for the experimental period). The number of monthly suppressible crimes increased in the no-field interrogation area during the treatment period but declined when field interrogations resumed. The number of monthly suppressible crimes did not change significantly in the control area where field interrogations were conducted as usual or in the field interrogation area where specially trained officers conducted field interrogations. The authors concluded that some field interrogation activity, as opposed to none, provided a deterrent effect on suppressible crimes. Additionally, Boydstun (1975) reported that the level of field interrogation activities did not have a significant influence on the number of total arrests in the study areas nor on police-community relations. However, those stopped in the specially trained officer area for a field interrogation reacted more favorably than those who were stopped in the control areas where patrol officers conducting field interrogations had no special training.

**Cohen & Ludwig (2004)**

Cohen and Ludwig (2004) examined the effect of directed police patrols on illegal gun carrying and violent crime using data from the Pittsburgh Police Department’s 1998 firearm suppression patrol (FSP) program. The FSP patrol officers proactively searched for illegally carried guns and initiated contact with citizens mainly using traffic stops and stop and search activities with pedestrians. During the intervention period, Pittsburgh police patrols were deployed Wednesday through Saturday in high-crime areas. Cohen and Ludwig (2004) compared crime rates between the treatment and control areas, where police officers conducted business as usual, before and after the FSP program was launched to reduce gun crimes. Cohen and Ludwig (2004) found that the number of citizen-reported shots fired were reduced as much as 34% and hospital-treated gunshot injuries decreased by 71% in the target neighborhoods. Therefore, Cohen and Ludwig (2004) concluded that Pittsburgh’s FSP program appeared to have successfully reduced gun crime.

**Dennison & Finkeldey (2020)**

Dennison and Finkeldey (2020) used data from the National Longitudinal Study of Adolescent to Adult Health to examine the predicators and consequences of self-reported experiences of unfair treatment by police, and whether unfair treatment was associated with depression, self-efficacy, suicide ideation, and drug use. Dennison and Finkeldey (2020) measured unfair police treatment using a survey response that asked whether the respondent had ever been unfairly stopped, searched, or questioned by police. Dennison and Finkeldey (2020) found that respondents who reported more depressive symptoms, lower self-efficacy, suicide ideation, and drug use were associated with experiences of perceived unfair treatment by police. Further, Dennison and Finkeldey (2020) found that unfair police treatment appeared to negatively impact respondents’ well-being in adulthood. Dennison and Finkeldey (2020) reported that racial minorities were more likely to report unfair treatment by police. However, some consequences of unfair treatment were heightened among White, compared with Black, respondents, though the impact on depressive symptoms and self-efficacy were equally reported across racial groups. Dennison and Finkeldey (2020) attributed these disparities in consequences of unfair police treatment across racial groups to unfair police contact being an unfortunate but normal life-course event for Black respondents.

**Friedman et al. (2004)**

A total of 891 students from 18 public high schools in Chicago completed surveys in 2000 about their firsthand and vicarious encounters with police officers. Friedman et al. (2004) were interested in how youth experienced encounters with police, how they perceived police behaviors, and how these perceptions shaped youths’ attitudes toward police and feelings of safety in their neighborhoods. More specifically, Friedman et al. (2004) studied youths’ perception of police treatment during their encounters with police, how those perceptions impacted their fear of police, perceptions of safety, and a willingness to help police, as well as how vicarious encounters with police affected youths’ perceptions of the police and their communities. Over half (58%) of youth reported being stopped by police within the last year, with more than 60% reporting that police treated them disrespectfully during those stops. Friedman et al. (2004) found that students who reported disrespectful firsthand or vicarious interactions with police were less likely to trust and respect the police and less likely to report that police were fair or cared about their community. Overall, the majority of students reported that police had mistreated them and a significant portion of the sample reporting feeling fearful of the police and held negative attitudes toward them.

**Geller (2017)**

Using data from the Fragile Families and Child Wellbeing Survey (FFCWS), Geller (2017) measured the quantity, quality, and health implication of police contact among urban teens. Police contact was defined as being stopped by police directly or vicariously, meaning youth witnessed or heard of someone they knew being stopped by police. FFCWS surveyed nearly 5,000 children born in 20 large U.S. cities and their families over the first 15 years of the youths’ lives. FFCWS oversampled non-marital births to gather a socioeconomically disadvantaged sample composed of mostly Black and Hispanic families. At age 15, participating youth were asked whether they had ever been stopped by the police or knew of someone who had. Geller (2017) examined the health consequences, particularly symptoms of anxiety and post-traumatic stress disorder (PTSD), of teens coming into contact with the police during their lifetimes. To assess for changes in youths’ mental health, researchers used primary caregivers’ reports of the youth’s anxious and depressed behaviors at year nine. Twenty-three percent of youth reported being stopped by police and 75% reported vicarious police contact. Geller (2017) found that youths’ experiences with police were significantly associated with adverse mental health indicators. Specifically, youth that reported being stopped by police also reported greater levels of anxiety. Geller (2017) also found that anxiety and PTSD were related to police contact, and the intrusion of police stops also negatively affected youths’ mental health. Geller (2017) concluded that these findings suggested that police contact could impact health disparities among youth.

**Geller et al. (2014)**

Between 2012 and 2013, Geller et al. (2014) conducted a telephone survey of men aged 18 to 26 in New York City to examine young men’s experience with police and the potential impact police stops had on their mental health. Of the 1261 young men surveyed, 85% reported being stopped at least once in their lives, while almost half (46%) reported being stopped by police once in the last 12 months. Geller et al. (2014) found that respondents that reported higher levels of police contact, especially more intrusive contact, also reported experiencing higher levels of trauma and anxiety symptoms associated with their police encounters.

**Harris & Jones (2020)**

Harris and Jones (2020) used data from the Fragile Families and Child Wellbeing Survey (FFCWS) to investigate how police stops may have affected urban youths’ perceptions and attitudes about the police. Measured at age 15, 26.8% of youth reported ever being stopped by police, while 50.2% reported experiencing vicarious police contact, meaning they witnessed or heard of someone they knew being stopped by the police. Harris and Jones (2020) found that youth that reported direct or vicarious stops by age 15 also tended to report lower levels of respect for, and confidence in, police. Further, Harris and Jones (2020) found that both types of stops had a positive relationship with perceptions of procedural justice, but this positive outcome was mitigated when police were intrusive during stops. Harris and Jones (2020) concluded that direct and vicarious stops may also curb negative attitudes toward police in addition to the literature suggesting that direct and vicarious police stops may contribute to negative attitudes about the police.

**Hirschtick et al. (2020)**

Between 2015 and 2016, Hirschtick et al. (2020) surveyed 1543 adults, primarily from low-income communities of color in Chicago, using the Sinai Community Health Survey 2.0. Hirschtick et al. (2020) administered this cross-sectional multistage probability health survey to investigate whether frequent (i.e., persistent police exposure, defined as a high number of lifetime stops) or negative interactions with police (i.e., aggressive police exposure, defined as police threatening or using force against the respondent during their most recent police stop) were associated with the presence of post-traumatic stress disorder (PTSD) or depressive symptoms. Hirschtick et al. (2020) found that 19% of women reported a high number (more than 3) of lifetime police stops, whereas 24% of men reported a high number (more than 15) of lifetime police stops. Hirschtick et al. (2020) also found that men who had persistent police exposure had three times greater odds of current PTSD symptoms, while women who had persistent police exposure had two times greater odds of current PTSD symptoms. Current depressive symptoms were not significantly associated with either persistent nor aggressive police exposure for men or women.

**Hofer et al. (2019)**

Hofer et al. (2019) used data from the age 15 assessment of the Fragile Families and Child Wellbeing Study (FFCWS) to examine the relationship between youth experiencing police contact and legal cynicism, controlling for demographics, delinquency, neighborhood context, and stop outcome. Youth that did not report having any direct or vicarious contact with police were excluded from the sample, meaning that youth with direct police experience were compared with youth that had experienced vicarious police contact. Direct police contact was defined as ever being stopped by the police, while vicarious police contact was defined as ever having seen someone stopped by police in their neighborhood or at school. Legal cynicism was measured using a scale that was adopted from questions used in the Pathways to Desistance Study (Harris et al., 2009). Approximately five percent of the youth sample experienced only direct police contact, while 62% of youth experienced only vicarious contact (with 33% reportedly experiencing both direct and vicarious police contact). Hofer et al. (2019) found that youth that had experienced direct or both direct and vicarious contact with police had higher levels of legal cynicism when compared to youth that only experienced vicarious police contact. Police contact that involved harsh language and frisking, and other situational factors, were also associated with higher levels of legal cynicism.

**Jackson et al. (2021)**

Jackson et al. (2021) used data from the 2012 to 2019 waves of the UK Millennium Cohort Study (MSC), which follows a nationally representative birth cohort of children born between September 2000 and January 2002, to examine the consequences of police-initiated encounters on youths’ mental health. At year 14 of data collection, youths were asked if they had ever been stopped and questioned by police. Jackson et al. (2021) found that youth that had experienced police stops in their lifetime by age 14 also reported significantly higher rates of self-harm (i.e., a 52% increase in the rate of self-harm within the past year) and a 125% increase in the odds of attempted suicide by age 17. Jackson et al. (2021) also found that mental distress mediated the association between police stops, self-harm, and suicide attempts, in that approximately 18% to 40% of associations between stops and outcomes were the result of mental distress.

**Jackson et al. (2020a)**

Using data from the Fragile Families and Child Wellbeing Study (FFCWS) that tracks at-risk, urban-born youth, Jackson et al. (2020) examined how low self-control and legal cynicism were associated in samples of youth with and without experiences of direct and vicarious police stops by age 15. Vicarious police contact was assessed by asking youth whether they had seen someone stopped by police in their neighborhood or at school, or whether they knew someone who had been stopped by police. Jackson et al. (2020) were interested in whether youths that reported lower levels of self-control more likely to have higher levels of legal cynicism, no matter their direct or vicarious stop history, and whether the perceptions of police-youth encounters of youth with vicarious police contact and youth with direct police contact confounded the association between low self-control and legal cynicism. Jackson et al. (2020) found that low self-control was positively associated with legal cynicism, and yielded the largest impact on legal cynicism compared to all other variables, across samples of youth with and without experiences of direct or vicarious police stops. Jackson et al. (2020) further found that the association between low self-control and legal cynicism was strongly related to youths’ perceptions of these stops.

**​​Jackson et al. (2020b)**

Jackson et al. (2020) investigated the relationship between youth being exposed to police stops and sleep behaviors, and whether stigma and indicators of post-traumatic stress mediated this relationship. Jackson et al. (2020) used data from the 2014 to 2017 wave of the Fragile Families and Child Wellbeing Study (FFCWS) when respondents were aged 15 on average. Vicarious police stops were measured by asking youth whether they had ever seen someone being stopped by police in their neighborhood or at school, or if they knew of anyone who had been stopped by police. Jackson et al. (2020) found that any police exposure, direct or vicarious, was associated with sleep deprivation and low sleep quality among youth. Moreover, Jackson et al. (2020) found that youth that reported they experienced direct police stops that were intrusive (e.g., involving frisking, searching, harsh language) had particularly disruptive sleep patterns. However, the association between intrusive direct police stops and poor sleep quality was non-significant when stigma and post-traumatic stress were added to the analysis.

**Kochel & Nouri (2021)**

Kochel and Nouri (2021) suggested that effective strategies to promote residents’ feelings of safety may depend on the level of violence present in their neighborhoods. For example, individuals living in high-crime areas may interpret environmental cues about their safety and police presence differently if violence is a part of their daily lives. Kochel and Nouri (2021) analyzed 820 household surveys of residents from high-, moderate-, and low-violence areas to assess residents’ perceptions of safety in their neighborhoods in order to suggest strategies that may promote feelings of safety across areas with varying levels of violence. Residents were asked about their perceptions of police fairness, measured in terms of procedural justice and frequency of police misconduct, as well as police effectiveness, experience with police (asking respondents whether they had been stopped in the last six months), collective efficacy, neighborhood violence, presence of guns, and respondent demographics. Collective efficacy, or the ability of neighbors to exercise social cohesion and informal social control to curb violence and enhance local safety, was measured based on residents’ perceptions of social cohesion and informal social control in their neighborhoods. Kochel and Nouri (2021) found that residents’ feelings of safety varied based on neighborhood context: the highest levels of collective efficacy were found in low-violence areas; residents in moderate-violence areas that heard gunfire reported the largest decline in feelings of safety; and, the highest proportion of residents that reported being stopped by police within the last six months lived in the high-violence areas. However, Kochel and Nouri (2021) found that residents across all types of neighborhoods reported that collective efficacy was an important factor that affected their feelings of safety.

**Lee et al. (2017)**

Lee et al. (2017) examined adolescents’ experiences with the formal legal system, false stops by police, and reflected parental perceptions as measures of labeling and whether these factors impacted the propensity for criminal behavior in adulthood. False stops were defined as instances in which police stopped youth for suspicion of a crime that they youth did not commit. Lee et al. (2017) analyzed data from 357 child, teen, and adult interviews collected from the Lehigh Longitudinal Study, which recruited individuals from child welfare caseloads and other high-risk group settings in Pennsylvania. Researchers found that criminal behaviors in adulthood were associated with adults who believed that their parents perceived them as deviant, but formal legal system involvement and false stops by the police were not associated with future criminality in adulthood.

**Lerman & Weaver (2014)**

Lerman and Weaver (2014) investigated how increased rates of stop-and-frisks in disadvantaged neighborhoods affected community engagement with the state. Researchers measured civic engagement using non-emergency 311 calls for service as a proxy for callers’ willingness to engage with their local government. Lerman and Weaver (2014) analyzed 311 requests from 2010 to 2011 in New York City. They found that higher rates of police stops were associated with higher rates of community engagement, yet when there were high levels of police officer intrusiveness (e.g., searches, use of force) associated with stops, then there were lower levels of civic engagement. In other words, the nature of the police contact may have mattered more than the extent or frequency of that police contact. The authors concluded that aggressive policing tactics in impoverished urban neighborhoods affected the willingness of residents to call on their local government to resolve local issues. Lerman and Weaver (2014) suggested that their findings demonstrated that when residents perceive police as fair, they may be more likely to engage with their local government to help them resolve issues in their homes and neighborhoods.

**Lewis & Wu (2021)**

Lewis and Wu (2021) surveyed 301 African American students attending a historically Black university to examine whether either experiencing a police stop or community violence victimization was more predictive of the respondent experiencing post-traumatic stress disorder symptoms. Being victimized by community violence was defined as ever having been chased or ever having seen someone be chased by a person or group of people. Almost 71% of the sample reported being stopped by police and approximately 34% reported experiencing community violence. Respondents who had been stopped by police more than five times were associated with elevated PTSD scores, suggesting that the more police stops a respondent experienced, the higher the likelihood that they would develop PTSD symptoms. The authors concluded that experiencing police stops was a greater predictor of subsequent PTSD symptoms than directly or vicariously experiencing community violence, though both police stops and community violence predicted the development of PTSD symptoms in the sample.

**MacDonald et al. (2016)**

Macdonald et al. (2016) evaluated the effects of the New York Police Department’s (NYPD) Operation Impact on reported crimes and arrests from 2004 to 2012. Operation impact was designed to deploy extra police officers to high-crime areas, in which police officers were encouraged to make investigative stops. Police commanders, police crime analysts, and the Police Commissioner worked together to identify crime hot spots in their precincts to be designated impact zone areas where additional police academy graduates would be deployed. Macdonald et al. (2016) compared crime and arrest rates before and after the census block groups were designated as impact zones, to other census block groups that were outside of the designated impact zones but in the same respective NYPD precincts. Macdonald et al. (2016) found that Operation Impact had a small but statistically significant association with total crime reduction in the impact zones. Specifically, declines in total reported crimes, increases in total reported arrests, and increases in investigative stops for suspected crimes were significantly associated with impact zones. However, only investigative stops made based on probable cause indicators of crime were associated with crime reductions, while the investigative stops based on suspicious behavior did not have an impact on crime. This finding suggested that most of the investigative stops did not affect decreases in crime. As a result, Macdonald et al. (2016) concluded that more focused investigative stops could have an impact on reducing crime.

**McCandless et al. (2016)**

Operation BLUNT 2, a Metropolitan Police initiative, aimed to reduce knife crime in London in 2008. McCandless et al. (2016) evaluated whether the activities of Operation BLUNT 2 contributed to a decrease in crime. Based on the rate of knife crimes, 32 London boroughs were divided into tiers. The most manpower and resources were deployed to the ten Tier 1 boroughs, followed by six Tier 2 boroughs, with the remaining 16 Tier 3 boroughs conducting policing business as usual (though there was a slight increase in stops). Consequently, Tier 1 boroughs experienced a tripling of weapons searches during the treatment period. McCandless et al. (2016) found no statistically significant decrease in crime at the borough level as a result of the increased rate of weapons searches carried out during Operation BLUNT 2. The authors suggested that crime may have been reduced as a result of the stop and search activities, but these effects may have gone undetected at the borough level since boroughs are such large geographic units of analysis.

**McFarland et al. (2019)**

McFarland et al. (2019) examined the relationship between police exposure and adolescents’ health, and whether this relationship is mediated by adolescents’ perceptions of procedural injustice occurring during police encounters. McFarland et al. (2019) used data taken from the Fragile Families and Child Wellbeing Study (FFCWS). Police contact was measured as direct or vicarious exposure to police in the respondent’s lifetime, assessed at age 15, while McFarland et al. (2019) used adolescents’ self- and caregiver-reports to assess health measures. Direct police contact was measured by asking respondents if they had ever been stopped by police, whereas vicarious police contact was measured by asking if they personally knew someone that had been stopped by police. Procedural injustice was measured by asking youth who had been directly or vicariously exposed to police encounters the degree to which the police explained why they had stopped the person, treated the person stopped with respect (i.e., dignity and courtesy), and respected the stopped person’s rights. McFarland et al. (2019) found that youth that had exposure to direct or vicarious police stops also reported poorer health outcomes, which perceived procedural injustice exacerbated, compared to youth that never experienced either type of police contact. Additionally, the relationship between police exposure and self-reported health was more pronounced for Black and Hispanic respondents when compared to White youth. The authors noted that police exposure was not related to caregiver-reported health, possibly demonstrating some bias in the self-reported health outcomes. However, the authors believed that they had mitigated any bias, in part because they had measured self-reported health outcomes with youth at year nine.

**McGarrell et al. (2002)**

The Indianapolis Police Department deployed direct patrols in two police districts, the East District and the North District, for 90 days in 1997 to suppress gun crime. Police in the two beats that composed the East District employed a general deterrence strategy that encouraged a high number of patrol officers to stop high rates of people to deter crime. Officers in the two beats that composed the North District employed a more targeted deterrence strategy in which fewer officers stopped fewer people but arrested three times as many people per 100 stops as compared to the East District. As a result of these strategies, homicide declined in both districts. However, the North District was also able to reduce gun crime, aggravated assault with a gun, and armed robbery using fewer resources than the East District due to their more targeted approach to stopping suspicious people. In fact, the East District’s more generalized deterrence strategy was not effective at reducing gun crimes besides possibly in instances of murder. The authors believed that the North District’s targeted approach was more successful because people most likely to commit gun crimes were wary of the increased police activity. Overall, McGarrell (2002) found that directed police patrol activities that included targeted stop activities reduced violent gun crime in Indianapolis, and the community generally accepted these programs given their positive outcomes.

**Murray (2014)**

Murray (2014) examined the effect of vehicle and pedestrian stop and search activities on crime in two distinct areas of Scotland. Murray analyzed the differences in stop and search approaches in Strathclyde, a proactive and thus high-stop area, with Grampian, which followed a reactive approach and thus was a low-stop area. Murray (2014) found that there was no causal relationship between stop and search levels and offending behavior despite the different approaches taken to stop and search activities.

**Murray et al. (2021)**

Murray et al. (2021) studied youth aged 12 to 15, who had been stopped and searched by police in two Scottish and two English cities to determine whether their stop and search experiences affected their trust in police, perceptions of police legitimacy, and law-abiding behavior. Murray et al. (2021) used data collected between 2014 to 2015 from a third iteration of a cross-sectional city-based survey of students’ experiences of crime called the International Self-Report Delinquency Survey (ISRD3). Murray et al. (2021) found that Scottish teens reported experiencing higher rates of stop and search encounters than their English counterparts. In fact, Scottish teens reported three times as many stop and search encounters than the teens in English cities. Scottish teens also held less favorable attitudes toward police and police legitimacy, as compared to English teens who reported experiencing lower rates of stop and search encounters. However, the authors suggested that any level of stop and search activity may result in lower levels of perceived police legitimacy, which may contribute to increased offending behaviors among adolescents.

**Ratcliffe et al. (2011)**

Ratcliffe et al. (2011) conducted a randomized controlled trial examining the effect of police foot patrol on violent crime hot spots in Philadelphia (PA). In collaboration between Temple University and the Philadelphia Police Department, 120 violent crime foot beats were assigned to either treatment or control conditions. Treatment officers patrolled in pairs conducting foot patrol from 10:00 p.m. to 2:00 a.m. Tuesday through Saturday of each week. The intervention lasted 12 weeks and researchers compared violent crime counts between the intervention period and the 12 weeks prior to the intervention period. Results indicated that treatment foot beats were associated with a 23% reduction in violent crime compared to control foot beats. Examination of potential displacement effects indicated a slight displacement of violent crime to buffer areas, but this increase was smaller in magnitude than the decrease in crime attributable to the intervention.

**Rosenbaum et al. (2005)**

Rosenbaum et al. (2005) measured attitudes before and after direct and vicarious contact with the police over the past year among Black, Hispanic, and White Chicago residents from 2001 to 2002. Researchers defined direct police contact as any police-initiated contact with the respondent within the past year. Vicarious police contact was defined as hearing about or knowing someone who had a direct police encounter. Attitudes toward police were assessed in terms of respondents’ perceptions of police responsiveness to community concerns, their ability to prevent crime, and their level of politeness. Rosenbaum et al. (2005) found that participants with simply direct police experience did not change their attitudes. However, Rosenbaum et al.’s (2005) findings suggested that it was not the exposure itself, but the nature of the police contact, that may be responsible for shaping attitudes. For example, negative citizen-initiated contacts were related to significant attitudinal changes that resulted in poor attitudes toward police. However, negative police-initiated contact encounters were not associated with any change in respondents’ attitude toward police. Researchers speculated this may be the result of the nature of the police contact or preconceived notions about the police. Respondents may have had low expectations for police-initiated contact and therefore their attitudes did not change. However, if citizens initiated police contact, most likely needing help, and they unexpectedly experienced a negative encounter, they may be more likely to recall those negative details when shaping a more negative outlook about police. Participants with vicarious police experience over the past year did show changes in attitudes, demonstrating that different types of police contact may be responsible for how the public’s attitudes about police are shaped.

**Sherman & Rogan (1995)**

Sherman and Rogan (1995) evaluated the Kansas City Gun Experiment, a police patrol program that lasted for 29 weeks from July 1992 to January 1993 in Kansas City, Missouri. The program aimed to reduce gun violence, including drive-by shootings and homicides, in beats experiencing rates of homicide 20 times the national average. The Kansas City Police Department deployed directed police patrols to the target area to increase the seizure rate of illegally carried guns to reduce the number of gun crimes in those areas. As a result of the intervention, police seizing guns rose by more than 65% in the target area, where gun crimes were reduced by 49%. The number of gun crimes and seizures did not change in a comparable beat several miles away, and no notable displacement of gun crimes occurred outside of the target beat. Drive-by shootings and homicides were also reduced in the target area but not in the comparison area, which actually experienced an increase in drive-by shootings. Community surveys of residents in the target area revealed that they had become less fearful of crime after the direct police patrol program. Sherman and Rogan (1995) found that the Kansas City Gun Experiment was successful in reducing gun crime.

**Singer (2013)**

Singer (2013) examined the relationship between police stops and young men’s attitudes toward policing and police officers in London. Singer (2012) examined the degree to which trust (i.e., the police serve their best interest), respect (i.e., police treat them with dignity), neutrality (i.e., police are not biased), and voice (i.e., police give them an opportunity to express their viewpoint) were perceived as being present for young men aged 16 to 30 years that were stopped or not stopped within the last 12 months. Singer (2012) used data from surveys of Black and Minority (BME) and White young men. Singer (2012) found that more BME respondents reported being stopped within the past year than White respondents. Most of the total sample across races, and whether they had been stopped or not, were positive about policing. However, those who were stopped were more likely to report not feeling confident in the police in their communities.

**Slocum et al. (2016)**

Slocum et al. (2015) investigated how being stopped or arrested, and any perceived procedural injustice that occurred during these encounters with police, affected youth’s propensity for future delinquency. Slocum et al. (2015) used data from the second National Evaluation of the Gang Resistance Education and Training (G.R.E.A.T.) program (2006-2013) that aimed to prevent youth gang involvement and violence. Participants at the beginning of the evaluation were students from 31 middle schools across seven U.S. cities. Slocum et al. (2015) found that the nature of the stop, in terms of the youth perceiving that the police encounter was procedurally just, was more important in predicting future delinquency than the act of being stopped by police itself. Specifically, future delinquency was mitigated, though not eliminated, when police contact was perceived favorably.

**Sundaresh et al. (2020)**

Sundaresh et al. (2020) examined the relationship between being exposed to the U.S. criminal legal system (i.e., being exposed to police stops, arrests, and incarceration) and well-being (i.e., physical, mental, social, spiritual, and overall life evaluation). Sundaresh et al. (2020) used data from 2,815 participants who had responded to the 2018 Family History of Incarceration Survey that studied the personal and family incarceration experience of a nationally representative sample. Sundaresh et al. (2020) found that exposure to the criminal legal system was related to lower overall well-being, with longer or multiple instances of exposure being associated with lower well-being, compared to participants who reported they had not been exposed to the criminal legal system in the United States. Further, participants who reported that they had been stopped by police experienced low well-being similar to the participants who had reported being incarcerated multiple times.

**Swaner & Brisman (2014)**

Swaner and Brisman (2014) surveyed 133 non-delinquent youth to understand their perceptions of fairness of the law, confidence in the court system, and attitudes toward police officers (i.e., measures of legal cynicism). Non-delinquent youths (in grades eight through 12) were sampled from a voluntary program at a courthouse located in a socially and economically disadvantaged community (i.e., Red Hook) with high crime rates in Brooklyn, New York. Youth were asked if they had been stopped by the police within the last 12 months. Swaner and Brisman (2014) found that legal cynicism was prevalent among the youth in this non-delinquent sample. Youth reported feelings of discrimination and unfair enforcement of the law. Further, when youth reported a recent negative experience with police, the experience tended to negatively affected their attitudes toward police, whereas a recent positive encounter with police did not affect their attitudes.

**Testa et al. (2021)**

Testa et al. (2021) used data from wave V (2016-2018) of the National Longitudinal Study of Adolescent to Adult Health to examine 12,057 participants’ relationship between experiences of unfair police treatment and sleep behavior. Unfair police treatment was measured by asking whether the respondent had ever been unfairly stopped, searched, or questioned by the police in their lifetime. Testa et al. (2021) found that participants who reported ever experiencing unfair police treatment also tended to report negative sleep behaviors, such as sleeping fewer than six hours and having trouble sleeping. Testa et al. (2021) found that depressive symptoms and general health were responsible for a minority of these associations. Testa et al. (2021) concluded that experiencing unfair police treatment appeared to be associated with lower sleep quantity and quality in this sample.

**Turney (2021)**

Turney (2021) examined the relationship between adolescents’ experiencing depressive symptoms and their direct and vicarious exposure to police. Personal (i.e., direct) police contact was measured as reporting ever being stopped by police, while vicarious police contact was measured by reports of participants ever seeing someone, or knowing someone who had been, stopped by police. Turney (2021) drew data from 3,437 adolescents who had participated in the Fragile Families and Child Wellbeing Study (FFCWS) in 20 U.S. cities at age 15. Turney (2021) found that direct and vicarious police contact were associated with depressive symptoms compared to respondents who reported never experiencing any type of police contact. Further, police stops that were considered more intrusive (e.g., frisking, searching) were also positively associated with depressive symptoms. Finally, the relationship between any type of police contact and symptoms of depression was concentrated among girls and Black youth in the sample.

**Tyler et al. (2014)**

Tyler et al. (2014) investigated whether perceived police legitimacy influenced respondents to follow the law, whether police stops influenced police legitimacy, and whether police actions perceived as just or unjust during police encounters affected respondents’ attitudes toward police. The measure of legitimacy included assessing the respondents’ level of trust and confidence in the police, their perception of obligation to obey police, and shared normative values. Tyler et al.’s (2014) sample consisted of young males aged 18 to 26 in a dense urban area (i.e., New York City) who were most likely to have experienced police stops. Respondents were asked to reflect on the most salient stop they experienced within the last year. Researchers found that police legitimacy and frequent stops, especially if the stops were intrusive, were significant predictors of criminal behavior. Respondents reporting higher levels of police legitimacy tended to be associated with lower rates of self-reported criminal behavior. On the other hand, multiple experiences of being stopped by police were associated with feelings of unfairness related to the stop. Exposure to policing did not predict legitimacy, but the perceived fairness of the police exposure was correlated with police legitimacy, in that respondents reported lower levels of police legitimacy when they also perceived the stop as unfair.

**Weisburd et al. (2016)**

Weisburd et al. (2015) examined the impact of rates of police stop, question, and frisks (SQFs) on daily and weekly crime rates in New York City. Researchers used street segments as the unit of analysis at a microgeographic level since crime can be highly concentrated in small areas. Weisburd et al. (2015) used data geocoded at the microgeographic level of SQF incidents and all non-traffic related crime incident data that occurred in New York City from 2006 to 2011. Weisburd et al. (2015) found that SQFs modestly but significantly impacted crime rates at the microgeographic street segment level across weekly time periods.

**Wheelock et al. (2019)**

Wheelock et al. (2019) examined factors that could influence citizens’ satisfaction with police. Specifically, they investigated whether satisfaction with police differed across races/ethnicities, whether experiences with police changed these differences, and whether procedural justice could explain varying attitudes toward police across races. Researchers measured satisfaction with police by asking respondents how satisfied they were that the Milwaukee Police Department’s officers were addressing crime (violent, property, and traffic), responsive to public concerns, honest and exhibiting integrity, having a good attitude, and competent. Respondents were also asked if they had experienced Milwaukee police-initiated contact in the past year. The U.S. Census American Community Survey, the Wisconsin Incident-Based Reporting System, and the City of Milwaukee Police Satisfaction Survey of 2014 were used to understand respondents’ attitudes toward and experiences with the police. Wheelock et al. (2019) found that Black respondents were significantly less satisfied with police than White and Hispanic respondents and that contact with police did not account for these racial differences. They found instead that procedural justice was a significant predictor of police satisfaction, but perceptions of safety was the factor that most clearly accounted for racial differences in police satisfaction.

**Wiley & Esbensen (2016)**

Wiley and Esbensen (2016) investigated the effect of youth being stopped or arrested on future delinquency and attitudes using a labeling theory framework. Wiley and Esbensen (2016) used three waves of data from the second National Evaluation of the Gang Resistance Education and Training (G.R.E.A.T.) program (2006-2013) that aimed to prevent youth gang involvement and violence. Participants at the beginning of the evaluation were students from 31 middle schools across seven U.S. cities. Researchers compared youth that were stopped, arrested, or had no police contact at Time 2 and measured delinquency and attitudes at Time 3. Researchers found that youth who were stopped or arrested were also associated with future delinquency and exacerbated deviant attitudes.

**Wiley et al. (2013)**

Wiley et al. (2013) examined whether experiencing police contact influenced youth to subsequently offend using a labeling theory framework. Wiley et al. (2013) analyzed four waves of longitudinal data from 2,127 middle-school students in seven U.S. cities to compare youth with no police contact to youth who were stopped by police (and those who were arrested) within the last six months. The data analyzed derived from the second National Evaluation of the Gang Resistance Education and Training (G.R.E.A.T.) program that aimed to prevent youth gang involvement and violence. Wiley et al. (2013) found that youth who were stopped or arrested were associated with higher levels of future delinquency compared to youth with no police contact. They also found that social bonds, deviant identity formation, and delinquent peers mediated the relationship between that contact and subsequent offending.

1. Appendices B and C are taken directly from Higginson, A., Eggins, E., Mazerolle, L. and Stanko, E. (2015). *The Global Policing Database [Database and Protocol].* [↑](#footnote-ref-1)
